# TNF-α induces type I IFN signalling to suppress neurogenesis and recruit T cells

**DOI:** 10.1038/s41467-026-74104-x

**Published:** 2026-07-07

**Authors:** Tinne A. D. Nissen, Arishma Baig, Sahand Farmand, Daniel T. Rock, Sandra Shibu, Hyunah Lee, Lauren A. O’Neill, Vikki Houghton, Susan John, Linda S. Klavinskis, Sandrine Thuret

**Affiliations:** 1 Department of Infectious Diseases, School of Immunology & Microbial Sciences, King’s College London, London, UK; 2 Department of Basic and Clinical Neuroscience, Institute of Psychiatry, Psychology and Neuroscience, King’s College London, London, UK; 3 Peter Gorer Department of Immunobiology, King’s College London, London, UK

**Keywords:** Cellular neuroscience, Neural stem cells, Neurogenesis

## Abstract

Adult hippocampal neurogenesis is essential for learning, memory, and mood regulation, and its disruption is implicated in ageing, neurodegeneration, and mood disorders. However, the mechanisms linking inflammation to adult hippocampal neurogenesis impairment remain unclear. Here, we identify chronic tumour necrosis factor-alpha signalling as a key driver of neurogenic dysregulation via a previously unrecognised type I interferon autocrine/paracrine loop in human hippocampal progenitor cells. Using a female-derived human in vitro neurogenesis model, single-cell RNA sequencing, and functional T cell migration assays, we show that tumour necrosis factor-alpha induces a robust type I interferon response in hippocampal progenitor cells, promoting chemokine-mediated and CXC motif chemokine receptor 3-dependent T cell recruitment and suppressing neurogenesis. This inflammatory signalling cascade drives a fate switch in hippocampal progenitor cells from a neurogenic trajectory towards an immune-defensive phenotype, with critical implications for infectious and inflammatory disease pathogenesis. These findings uncover a key inflammatory checkpoint regulating human adult hippocampal neurogenesis and highlight potential therapeutic targets to restore neurogenesis in chronic inflammatory states.

## Abstract (web summary)

Here, the authors show that chronic TNF-α activates type I interferon signalling in human hippocampal stem cells, suppressing neurogenesis and promoting T-cell recruitment, thereby reprogramming cells toward an immune-reactive state.

## Introduction

Adult neurogenesis occurs in two main neurogenic niches in the mammalian brain: the subventricular zone (SVZ) lining the lateral ventricles and the subgranular zone (SGZ) of the dentate gyrus of the hippocampus^1^. Upon activation, hippocampal neural stem cells (NSCs) known as radial glia-like cells (RGLs) divide and differentiate into astrocytes or excitatory, granule cells, integrating into the granule cell layer. Initially discovered in rodents^2^, human adult hippocampal neurogenesis (AHN) has since been demonstrated using carbon dating^3^, postmortem tissue^4–7^, in vitro propagation of adult neural progenitor cells (NPCs)^8^, single nucleus RNA sequencing^9^, and spatial transcriptomics^10^. AHN in rodents enhances several cognitive functions, including memory and learning^11^, the development of individuality^12^, and mood regulation^13^.

Perturbations in AHN observed in ageing, viral infection, neurodegenerative diseases, and mood disorders are thought to contribute to cognitive decline and associated symptoms, given AHN’s role in memory and mood regulation^14–17^. Experimental enhancement of AHN reduces depressive-like behaviours^18^ and rescues memory and emotional deficits in Alzheimer’s disease (AD) mice^19^. Viral infections, including Zika virus, Herpes Simplex Virus type 1 (HSV-1), and SARS-CoV-2, have been shown to impair AHN in humans and mice by directly infecting NSCs or by inducing neuroinflammatory responses^16,17,20–22^. Emerging evidence suggests that inflammatory cytokines, elevated in AD^23^, viral infection^16,22^, ageing^24^, and in some individuals with mood disorders such as depression^25^, may underlie AHN deficits. In particular, the proinflammatory cytokine TNF-α has been implicated in both AD^26,27^ and depression^28,29^, and is known to negatively regulate AHN^30,31^. These observations support a model in which inflammation-driven suppression of AHN contributes to shared clinical phenotypes across disorders. Targeting this axis may represent a viable neuroprotective strategy^15^. Defining the molecular mechanisms by which inflammatory mediators, such as TNF-α, influence hippocampal NSC fate is crucial for developing interventions preserving AHN and its associated cognitive functions in inflammatory conditions.

Chronic TNF-α-mediated inflammation was recently shown to drive a functional switch in rodent olfactory NSCs, redirecting them from neuroregeneration toward immune recruitment^32^. These NSCs actively recruited inflammatory cells, including T cells, at the expense of their neurogenic capacity. We hypothesised that a similar switch could occur in human hippocampal progenitor cells (HPCs), contributing to impaired AHN and T cell infiltration observed in ageing and neurodegenerative disease, where TNF-α levels are persistently elevated^33–35^. Using an established in vitro model of human hippocampal neurogenesis^36–41^, we found that chronic TNF-α exposure suppressed neurogenesis and induced robust CXCL10 secretion. Single-cell RNA (scRNA) sequencing revealed that TNF-α activated type I IFN signalling via autocrine/paracrine signalling, a mechanism not previously described in central nervous system (CNS)-resident cells. This signalling cascade drove the secretion of chemokines that mediated CXCR3-dependent T cell chemotaxis and contributed to the anti-neurogenic effects of chronic TNF-α. Together, our findings support a model in which chronic TNF-α reprogrammes human HPCs from a neurogenic to a proinflammatory state, orchestrated by TNF-α-induced type I IFN signalling.

## Results

### TNF-α-driven functional and phenotypic reprogramming of HPCs

**Fig. 1:**
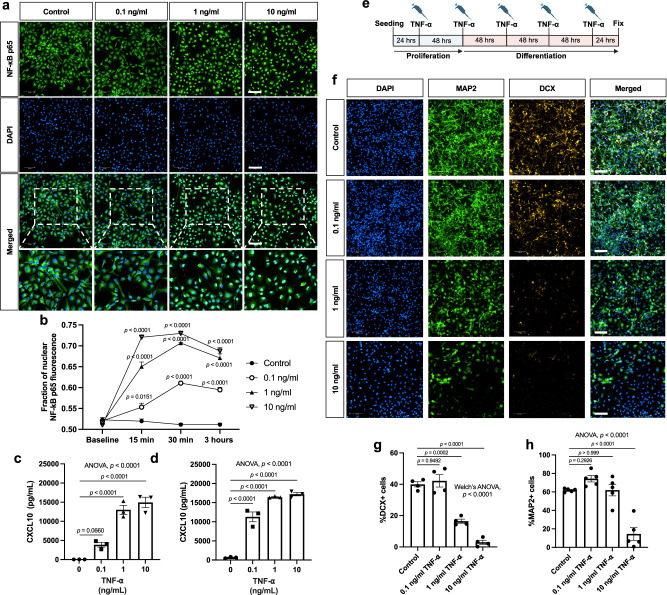
TNF-α drives immune signalling and decreases neurogenesis in human hippocampal progenitor cells. **a** Representative immunocytochemistry images of three independent experiments with similar results showing the expression of NF-κB p65 on HPCs treated ± 0.1 ng/ml, 1 ng/ml, or 10 ng/ml TNF-α for 15 min. Scale bar, 100 µm. **b** Quantification of the fraction of nuclear NF-κB p65 fluorescence intensity relative to total cellular fluorescence. Data represent mean ± SEM from *n* = 3 independent experiments. Statistical analysis: one-way ANOVA followed by Bonferroni multiple comparisons test. **c**, **d** Quantification of CXCL10 levels in culture supernatants measured by ELISA. **c**, HPCs under proliferation conditions treated for 48 h ±0.1, 1, or 10 ng/ml TNF-α. **d** HPCs under differentiation conditions treated for 7 days ±0.1, 1, or 10 ng/ml TNF-α. Data represent mean ± SEM from *n* = 3 independent experiments. Statistical analysis: one-way ANOVA followed by Bonferroni multiple comparisons test. **e** Schematic overview of the chronic TNF-α treatment regime for investigating its effect on neuronal differentiation. HPCs were treated under proliferation conditions for 48 h ±0.1, 1, or 10 ng/ml TNF-α before inducing differentiation for seven- days under treatment ± 0.1, 1, or 10 ng/ml TNF-α every 48 h. Created in BioRender. Nissen, T. (2026) https://BioRender.com/8yr92qo. **f** Representative images showing the expression of MAP2 (green) and DCX (orange) on seven days differentiated HPCs treated chronically ±0.1, 1, or 10 ng/ml TNF-α. Scale bar represents 100 µm. **g**, **h** Quantification of the percentage of DCX+ cells and MAP2+ cells, respectively, based on **f**. Data represent mean ± SEM. **g**, Data based on *n* = 4 independent experiments. Statistical analysis: two-sided Welch’s ANOVA followed by Games-Howell post hoc test for pairwise comparisons. **h** Data based on *n* = 5 independent experiments. Statistical analysis: one-way ANOVA followed by Bonferroni multiple comparisons test. Source data are provided as a Source Data file. HPCs human hippocampal progenitor cells, TNF-α tumour necrosis factor alpha, NF-κB nuclear factor kappa B, CXCL10 C-X-C motif chemokine ligand 10, MAP2 microtubule-associated protein 2, DCX doublecortin, SEM standard error of the mean.

To investigate if TNF-α reprogrammes human HPCs, we first confirmed that our cellular model expressed the TNF-α receptors TNFR1 and TNFR2 (Supplementary Fig. 1). To model chronic neuroinflammatory TNF-α exposure, we treated proliferating and differentiating HPCs with 0.1–10 ng/ml TNF-α, which is within or below the concentration range used in prior studies evaluating the effect of TNF-α on neural stem/progenitor cells^31,42–46^. TNF-α stimulation of HPCs induced rapid nuclear translocation of NF-κB p65, peaking at 30 min and remaining elevated for up to 3 h (Fig. 1a, b), consistent with sustained activation of a canonical proinflammatory pathway implicated in the regulation of neurogenesis^47^. To assess the functional relevance of TNF-α signalling in HPCs and their progeny, we evaluated the secretion of the NF-κB-regulated cytokine IL-6 and a panel of NF-κB-regulated chemokines. Levels were measured in the supernatant at 24 and 48 h after TNF-α treatment of the HPCs, as well as 24 h, 48 h, and seven days of TNF-α treatment in differentiating HPCs (Supplementary Fig. 2). Among the chemokines analysed (Supplementary Figs. 3–5), CXCL10 exhibited the most robust induction in response to TNF-α stimulation (Fig. 1c, d). To examine the effects of chronic TNF-α on neuronal differentiation, HPCs were pre-treated ± TNF-α under proliferative conditions for 48 h, followed by seven days of differentiation with continued TNF-α exposure every 48 h (Fig. 1e). TNF-α dose-dependently reduced the proportion of doublecortin (DCX)+ neuroblasts (Fig. 1g), while only the highest dose (10 ng/ml) decreased the percentage of cells expressing the neuronal marker microtubule-associated protein 2 (MAP2) (Fig. 1h). In parallel, TNF-α reduced total neurite length in both populations, indicating impaired morphological maturation (Supplementary Fig. 6). As TNFR1 signalling can promote apoptosis, we examined whether TNF-α induced apoptotic cell death in HPCs. Acute TNF-α treatment (24 h) did not affect the number of DAPI^+^ proliferating HPCs, whereas chronic exposure to 10 ng/ml TNF-α during differentiation reduced the number of DAPI^+^ cells. However, neither acute TNF-α treatment of proliferating HPCs nor chronic TNF-α exposure during differentiation increased the proportion of cells positive for nuclear cleaved caspase-3 (CC3) (Supplementary Fig. 7b, d). Thus, the reduced number of DAPI^+^ cells following chronic exposure to 10 ng/ml TNF-α during differentiation may reflect reduced proliferation, caspase-independent cell death, or early loss of apoptotic cells before fixation. Collectively, these findings suggest that chronic TNF-α functionally and phenotypically reprogrammes HPCs.

### Chronic TNF-α disrupts neuronal differentiation and upregulates a transcriptional type I IFN signature

**Fig. 2:**
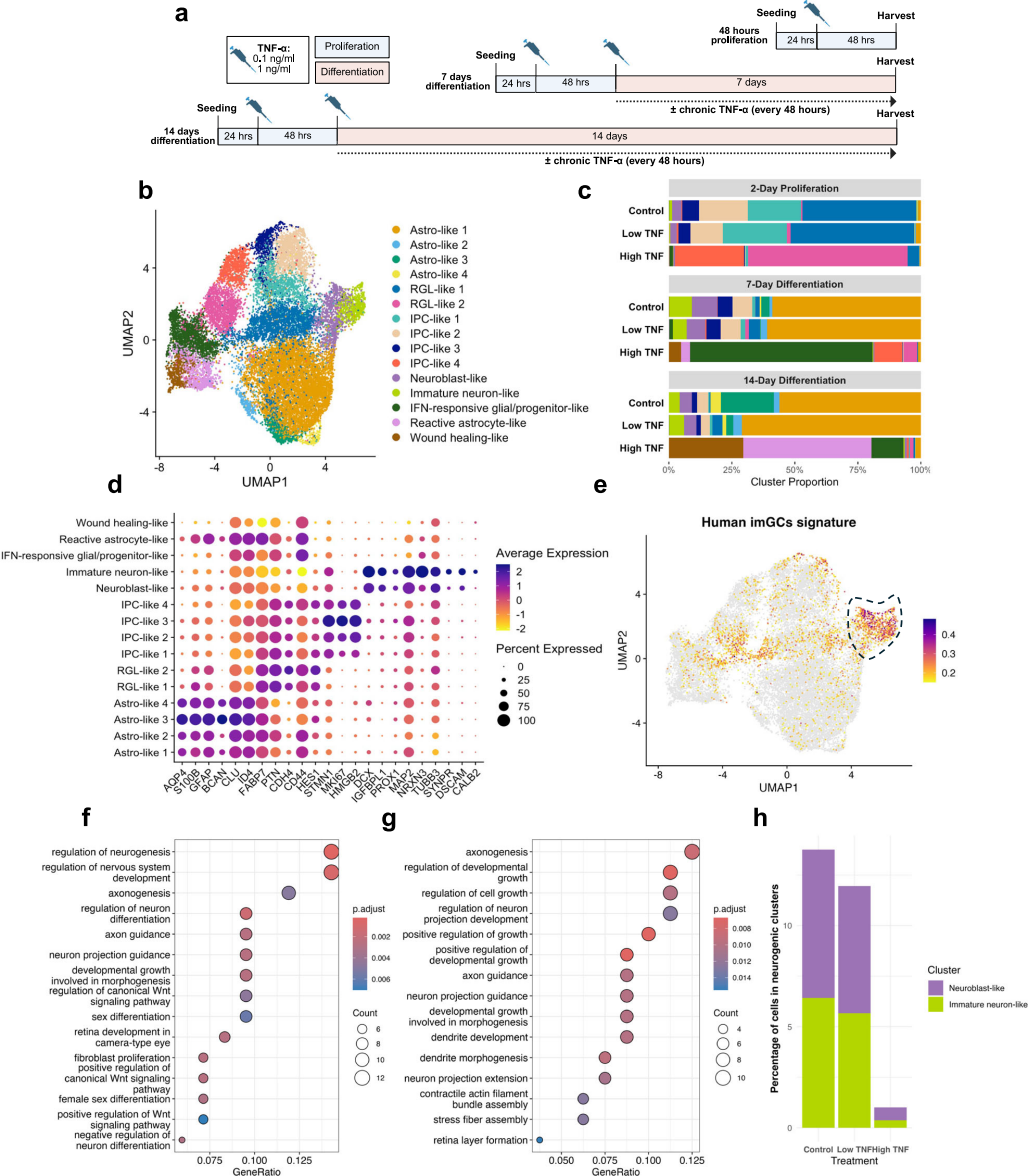
Chronic TNF-α impairs neuronal differentiation. **a** Schematic overview of the experimental design. Created in BioRender. Nissen, T. (2026) https://BioRender.com/r83i113. **b** UMAP plot showing transcriptional clustering of single cells. Colours indicate the 15 cell clusters identified. **c** Stacked bar graphs showing the frequencies of cell clusters shown in (**b**) across the time and treatment groups. Cluster proportions are calculated from the single library per condition/timepoint. The colouring of clusters is consistent between (**b** and **c)**.** d** Dot plot showing the expression of canonical marker genes for expected cell types across the cell clusters. **e** UMAP plot showing each cell scored against a human immature granule cells (imGCs) signature^9^. The outlined area highlights the overlap of the signature with the neuroblast-like and immature neuron-like clusters. **f**, **g** Dot plots showing Gene Ontology Biological Process enrichment based on the top 100 genes positively defining the neuroblast-like cluster and the immature neuron-like cluster, respectively. Enrichment analysis was performed using enrichGO (clusterProfiler) with Benjamini-Hochberg adjustment for multiple comparisons. **h** Bar plots show the percentage of cells belonging to the neuroblast-like cluster (purple) and immature neuron-like cluster (lime) within differentiation samples from 7 and 14 days by treatment condition: control, low TNF (0.1 ng/ml), and high TNF (1 ng/ml). Cluster proportions are calculated from the single library per condition/timepoint. scRNA-seq was performed with *n* = 1 10x library per condition/timepoint. Source data are provided as a Source Data file. TNF-α tumour necrosis factor alpha, UMAP uniform manifold approximation and projection, imGCs immature granule cells.

To elucidate the molecular mechanisms underlying the effects of TNF-α, we performed scRNA sequencing of HPCs exposed for 48 h to low (0.1 ng/ml) or high (1 ng/ml) TNF-α, as well as their progeny after 7 and 14 days of differentiation under chronic TNF-α exposure (Fig. 2a). These concentrations were selected because they were most likely to be pathophysiologically relevant and were the doses found to have relevant biological effects in our assays (Fig. 1c, g). After quality control, 22,133 cells were identified across 15 clusters, including astrocyte-like clusters, radial glial-like (RGL)/NSC-like clusters, intermediate progenitor cell (IPC)-like clusters, a neuroblast-like cluster, an immature neuron-like cluster, an IFN-responsive glial/progenitor-like cluster, a reactive astrocyte-like cluster, and a wound-healing-like cluster (Fig. 2b–d).

The neurogenic identity of neuroblast-like and the immature neuron-like cluster was confirmed by overlap with a human immature granule cell signature^9^ and by enrichment of neuronal biological processes and cellular compartment gene ontology (GO) terms (Fig. 2e–g and Supplementary Fig. 8). Critically, HPCs differentiated in the presence of high-dose TNF-α showed a marked reduction in cells within neurogenic clusters (Fig. 2h), indicating disrupted neurogenesis. Given the potential for TNFR1-mediated cell death, we assessed TNFR1 receptor expression and apoptosis/programmed cell death signatures. *TNFRSF1A* was detected across all clusters but least frequently detected in the neuroblast-like and immature neuron-like clusters (11.2% and 7.5%) (Supplementary Fig. 9), and apoptosis/programmed cell death signature scores were not increased with high-dose TNF-α-treated cells or in high-dose TNF-α-specific clusters (Supplementary Fig. 10a–d). As TNF-α-TNFR1 signalling can also induce regulated necroptosis via receptor-interacting serine/threonine-protein kinase 1 (RIPK1), receptor-interacting serine/threonine-protein kinase 3 (RIPK3) and mixed lineage kinase domain-like pseudokinase (MLKL), we examined the expression of these core necroptotic mediator genes (Supplementary Fig. 10e, f). *RIPK1* was broadly expressed, whereas *MLKL* was detected in few cells and *RIPK3* was absent across clusters, arguing against canonical RIPK1/RIPK3-dependent necroptosis.

**Fig. 3:**
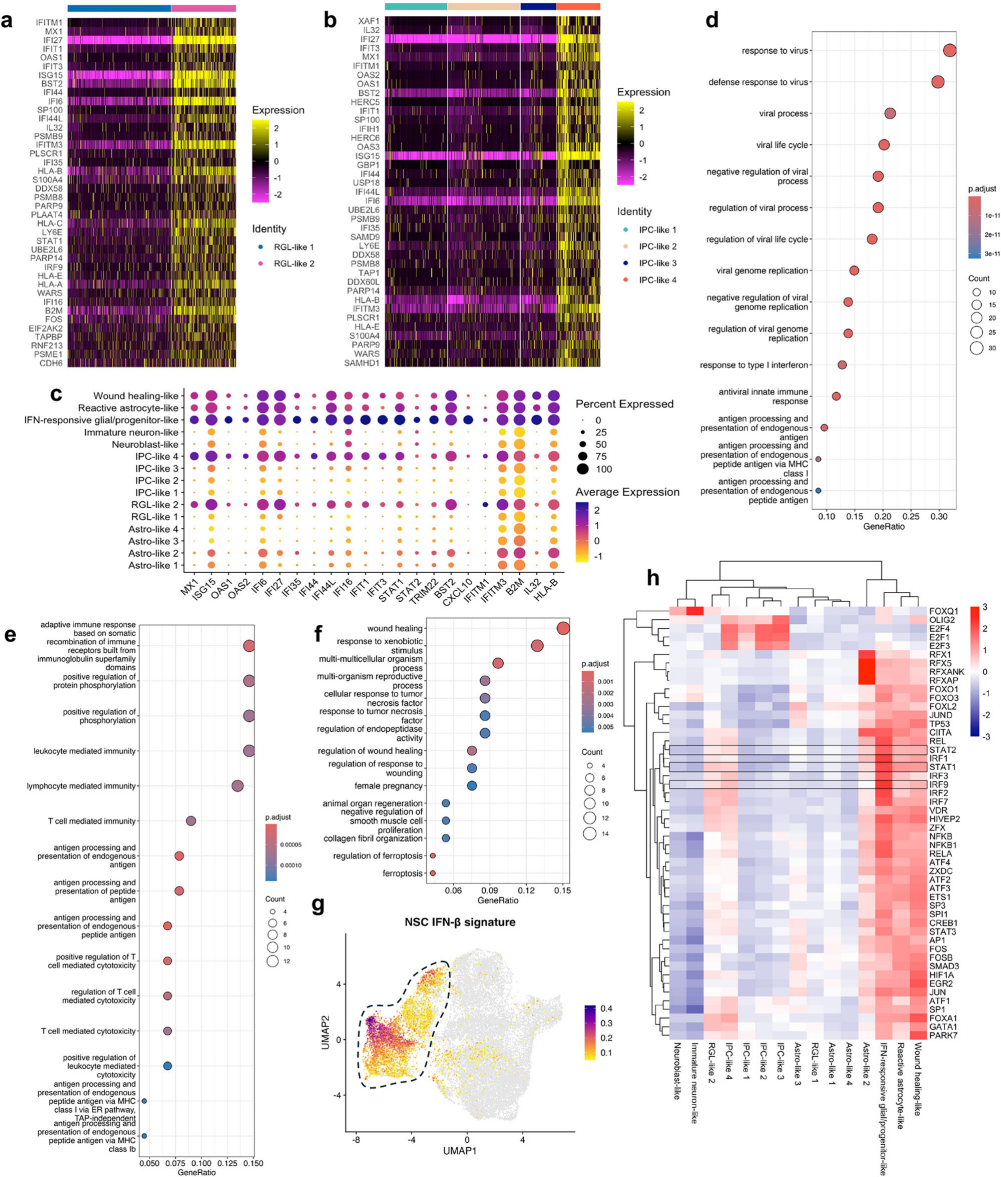
Chronic TNF-α drives the upregulation of type I IFN genes. **a** Heatmap of top 40 upregulated genes based on log fold change in the RGL-like 2 cluster as compared to the RGL-like 1 cluster. **b** Heatmap of top 40 upregulated genes based on log fold change in the IPC-like 4 cluster as compared to the IPC-like 1, IPC-like 2, and IPC-like 3 clusters. **c** Dot plot showing the expression of interferon response genes across the cell clusters. **d**, **e**, **f** Dot plots showing Gene Ontology Biological Process enrichment based on the top 100 genes positively defining the IFN-responsive glial/progenitor-like cluster, the reactive astrocyte-like cluster, and the wound healing-like cluster, respectively. Enrichment analysis was performed using enrichGO (clusterProfiler) with Benjamini-Hochberg adjustment for multiple comparisons. **g** UMAP plot showing each cell scored against an NSC IFN-β transcriptional signature. The outlined area highlights the overlap of the signature with the clusters specific to treatment with high TNF-α: the RGL-like 2, IPC-like 4, IFN-responsive glial/progenitor-like, reactive astrocyte-like, and wound healing-like clusters. **h** Heatmap showing the mean activity per cluster of the top 50 most variable transcription factors. STAT1, STAT2 and IRF9 are highlighted. scRNA-seq was performed with *n* = 1 10x library per condition/timepoint. Source data are provided as a Source Data file. TNF-α tumour necrosis factor alpha, IFN interferon, RGL radial glia-like, IPC intermediate progenitor cell, NSC neural stem cell, UMAP uniform manifold approximation and projection, STAT1 signal transducer and activator of transcription 1, STAT2 signal transducer and activator of transcription 2.

While control and low-dose TNF-α treated cells clustered together, high-dose TNF-α treated cells formed distinct clusters (Supplementary Fig. 11a). The RGL-like 2 cluster and the IPC-like 4 cluster, primarily composed of HPCs treated with high-dose TNF-α, exhibited strong upregulation of type I interferon (IFN) response genes including *ISG15*, *IFI27*, and *IFI6* (Fig. 3a, b). GO enrichment analysis highlighted “response to type I IFNs” and phagocytic vesicle components (Supplementary Fig. 11c, d; Supplementary Fig. 12). The type I IFN signature was detected across clusters specific to treatment with high-dose TNF-α (Fig. 3c). Although type I IFN signalling can sensitise cells to apoptosis^48^, we did not observe enrichment of apoptosis/programmed cell death signatures in clusters with a type I IFN signature (Supplementary Fig. 10).

HPCs differentiated in the presence of high-dose TNF-α clustered separately from HPCs differentiated under control and low dose TNF-α treatment. They split into three clusters: an IFN-responsive glial-progenitor-like cluster, a reactive astrocyte-like cluster, and a wound healing-like cluster. The IFN-responsive glial-progenitor-like cluster expressed *CLU*, *ID4*, *PTN*, and *CD44*, suggesting a glial/progenitor-like transcriptional phenotype and displayed strong upregulation of type I IFN-response genes (*ISG15*, *MX1*, *STAT1)*. Similarly, the reactive astrocyte-like cluster expressed *CLU*, *ID4*, *PTN*, and *CD44*, together with astrocytic lineage markers (*GFAP*, *S100B, AQP4*) and inflammatory/reactive astrocyte-associated genes (*TIMP1*, *CP, CRYAB)*^49^ suggesting a reactive astrocyte-like phenotype. Finally, the wound-healing-like cluster did not express canonical astrocytic markers but expressed genes implicated in wound healing (*MYLK*, *HBEGF*, *CD151*) which have recently been found in dedifferentiated and transcriptionally reprogrammed astrocytes following CNS injury^50^. GO-term analysis of these clusters indicated a shift toward aberrant, immune-reactive states with activation of a type I IFN-mediated antiviral programme (Fig. 3d–f). These findings indicate that high-dose TNF-α drives differentiating HPCs away from neurogenic and homoeostatic astrocytic transcriptional identities and toward reactive, IFN-responsive glial-like states.

Given that TNF-α does not directly induce type I IFN response genes, we hypothesised that a type I IFN autocrine/paracrine loop mediated the interferon-stimulated gene upregulation. HPCs and their progeny expressed the machinery to respond to type I IFNs (Supplementary Fig. 13), and cells from high-dose TNF-α-specific clusters strongly overlapped with an NSC-specific IFN-β response signature^51^ (Fig. 3g). Transcription factor activity analysis predicted STAT1, STAT2, and IRF9 activity in these clusters, consistent with IFN signalling (Fig. 3h). Together, these findings demonstrate that chronic TNF-α exposure induces type I IFN response genes in HPCs, suppresses neurogenesis, and promotes aberrant immune-reactive cellular states during differentiation.

### TNF-α drives type I IFN signalling through activation of IFNAR

**Fig. 4:**
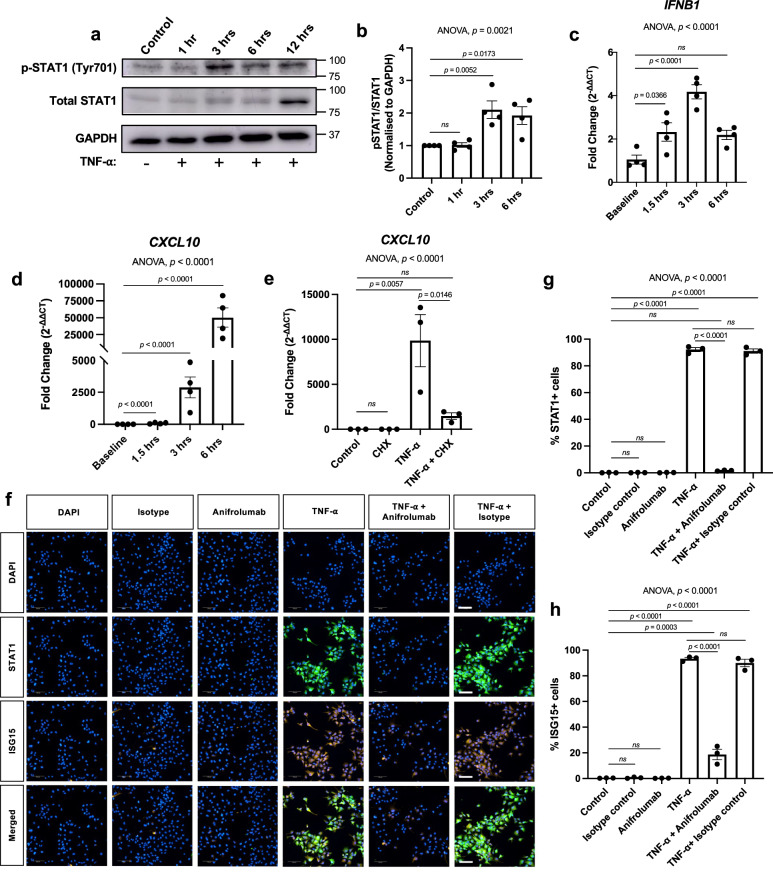
TNF-α drives type I IFN signalling in human hippocampal progenitor cells via autocrine/paracrine signalling through IFNAR. **a** Representative western blots of four independent experiments showing the activation of STAT1 in HPCs treated with 1 ng/ml TNF-α for 1 hr, 3 hrs, 6 hrs, and 12 hrs. Cell lysates were probed for phosphorylated STAT1 (p-STAT1 Tyr701), total STAT1, and GAPDH as a loading control. **b** Quantification of STAT1 activation in human hippocampal progenitor cells from (**a**). p-STAT1 and STAT1 intensities were normalised to GAPDH intensity before calculating the fold change p-STAT1/STAT1 relative to control. Data represent mean ± SEM from four independent experiments. Statistical analysis: one-way ANOVA followed by Bonferroni’s multiple comparisons. **c** RT-qPCR data showing the relative gene expression of *IFNB1* in response to treatment with 1 ng/ml TNF-α. Data is represented as mean ± SEM of four independent experiments. Statistical analysis: one-way ANOVA followed by Bonferroni’s multiple comparisons. **d** RT-qPCR data showing the relative gene expression of *CXCL10* in response to treatment with 1 ng/ml TNF-α. Data is represented as mean ± SEM of four independent experiments. Statistical analysis: one-way ANOVA followed by Bonferroni’s multiple comparisons. **e** RT-qPCR data showing the relative gene expression of *CXCL10* in HPCs treated for 6 h with 10 µg/ml CHX or DMSO ± 1 ng/ml TNF-α. Data is represented as mean ± SEM of three independent experiments. Statistical analysis: one-way ANOVA followed by Bonferroni’s multiple comparisons. **f** Representative images of three independent experiments with similar results showing the expression of STAT1 (green) and ISG15 (orange) in HPCs. Scale bar,100 µm. **g** Quantification of the percentage of STAT1+ cells based on (**f**). Data represent mean ± SEM of three independent experiments. Statistical analysis: one-way ANOVA followed by Bonferroni’s multiple comparisons. **h** Quantification of the percentage of ISG15+ cells based on (**f**). Data represent mean ± SEM of three independent experiments. Statistical analysis: one-way ANOVA followed by Bonferroni’s multiple comparisons. Source data are provided as a Source Data file. HPCs human hippocampal progenitor cells, TNF-α tumour necrosis factor alpha, IFN interferon, IFNAR interferon alpha receptor, STAT1 signal transducer and activator of transcription 1, GAPDH glyceraldehyde 3-phosphate dehydrogenase, RT–qPCR reverse transcription quantitative PCR, IFNB1 interferon beta 1, CXCL10 C-X-C motif chemokine ligand 10, CHX cycloheximide, DMSO dimethyl sulfoxide, ISG15 interferon-stimulated gene 15, SEM standard error of the mean.

As STAT1 negatively regulates human hippocampal neurogenesis^52^ and STAT1/STAT2 regulates the expression of type I IFN-regulated genes, we next assessed their activation in HPCs following TNF-α stimulation and investigated the underlying mechanism. Phosphorylation of both STAT1 and STAT2 was delayed, peaking at 3–6 h (STAT1: Fig. 4a, b; STAT2: Supplementary Figs. 14a, b; GAPDH stability in response to TNF-α: Supplementary Fig. 15), consistent with a mechanism of indirect activation. In line with an autocrine/paracrine loop, factors secreted downstream of TNF-α-mediated receptor engagement increased the percentage of ISG15+ HPCs (Supplementary Fig. 14c, d). TNF-α transiently induced *IFNB1* expression, peaking at 3 h (Fig. 4c), temporally correlating with STAT1/STAT2 activation, suggesting IFN-β as the likely mediator. TNF-α-induced *IFNB1* expression depended on NF-κB signalling (Supplementary Fig. 14e) but was independent of de novo protein synthesis (Supplementary Fig. 14f). While the cGAS-STING pathway has been implicated in TNF-α-induced *IFNB1* expression in other cell types^53^, the HPCs did not demonstrate *STING1* expression and expressed only minimal levels of *CGAS* (Supplementary Fig. 16a). Furthermore, they were functionally unresponsive to STING activation (Supplementary Fig. 16b, c), indicating that TNF-α-induced *IFNB1* expression occurs through a cGAS-STING-independent mechanism. As IRF1 is required for TNF-α–induced *IFNB1* expression in other cell types^54,55^, we examined its regulation in HPCs. TNF-α markedly upregulated *IRF1*, peaking at 3 h (Supplementary Fig. 17), preceding *IFNB1* induction and suggesting a potential regulatory role for IRF1 in HPC *IFNB1* induction. TNF-α-induced upregulation of the STAT1/STAT2-regulated chemokine CXCL10 has been found to depend on IFN-β^56^. Consistent with this, the delayed induction of *CXCL10* in response to TNF-α in HPCs required de novo protein synthesis, as shown by its sensitivity to cycloheximide treatment (Fig. 4d, e). To confirm that TNF-α induces type I IFN signalling via an autocrine/paracrine loop, we blocked the IFN-α/β receptor (IFNAR) using the antibody anifrolumab^57^. Anifrolumab fully abolished TNF-α-induced STAT1+ cell induction and significantly reduced ISG15+ cell upregulation (Fig. 4f–h). Similarly, Janus kinase (JAK) inhibition downstream of IFNAR using JAK Inhibitor I^58^ blocked TNF-α-induced ISG15 + HPC upregulation (Supplementary Fig. 14g, h). Finally, TNF-α upregulated the type I IFN-induced surface marker tetherin/BST-2 on HPCs (Supplementary Fig. 14i), which was recently shown to be elevated on dysfunctional NSCs in ageing neurogenic niches^33^. Collectively, these findings demonstrated that TNF-α drives autocrine/paracrine type I IFN signalling in HPCs via IFNAR and downstream JAKs likely mediated by IFN-β.

### TNF-α-conditioned media from HPCs and differentiated progeny drive CXCR3-dependent T cell chemotaxis

After elucidating the molecular mechanism of TNF-α-induced type I IFN signalling in HPCs, we next investigated its functional consequences. Recent studies highlight T cell infiltration into neurogenic niches in ageing and AD, with T cells found near NSCs^33,35,59–63^. We found high levels of the type I IFN-regulated chemokines CXCL10 and CXCL11 in the supernatant of HPCs and their differentiated progeny following TNF-α treatment. As both chemokines promote T cell chemotaxis via the CXCR3 receptor^64^, we hypothesised that their secretion could mediate CXCR3-dependent T cell recruitment. We established an in vitro transwell assay using primary human T cells from three healthy donors and validated that recombinant CXCL10 promotes CXCR3-dependent chemotaxis of both CD4+ and CD8 + T cells (Supplementary Figs. 18, 19).

**Fig. 5:**
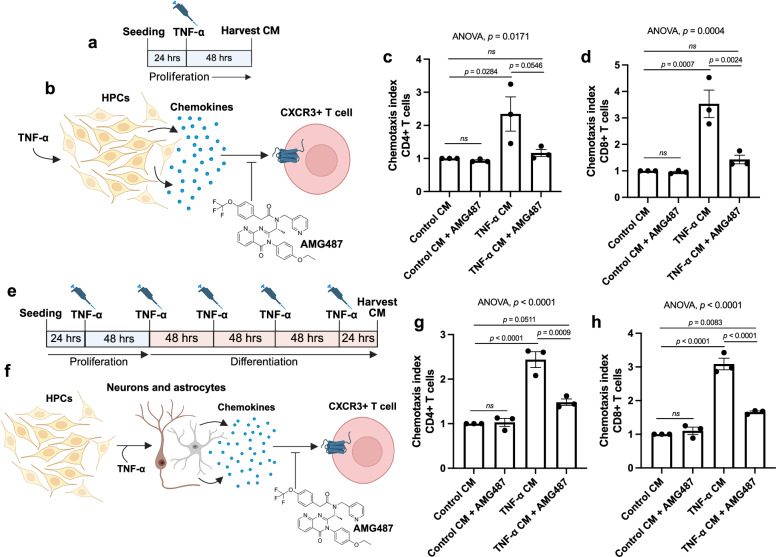
TNF-α-conditioned media from proliferating and differentiating HPCs drive CXCR3-dependent chemotaxis of primary, human T cells. **a** Schematic overview of the experimental design for generating conditioned media (CM) from HPCs treated for ± 1 ng/ml TNF-α for 48 hrs. Created in BioRender. Nissen, T (2026) https://BioRender.com/l84g395. **b** Chemokine secretion by HPCs in response to TNF-α stimulation assessed for its ability to induce CXCR3-dependent T cell chemotaxis. Created in BioRender. Nissen, T (2026) https://BioRender.com/96g3l83. **c**, **d** Quantification of the chemotactic response (chemotaxis index) of **c** CD4 + T cells and **d** CD8 + T cells treated ± 1 μM AMG487 or DMSO in response to control CM or TNF-α-CM from HPCs. Data is represented as mean ± SEM of three healthy donors. Statistical analysis: one-way ANOVA followed by Bonferroni’s multiple comparisons test. **e** Schematic overview of the experimental design for generating CM from differentiated HPCs treated chronically ±1 ng/ml TNF-α. Created in BioRender. Nissen, T (2026) https://BioRender.com/703pc4u. **f** Chemokine secretion by differentiated HPCs in response to chronic TNF-α stimulation assessed for its ability to induce CXCR3-dependent T cell chemotaxis. Created in BioRender. Nissen, T (2026) https://BioRender.com/jgooe2v. Quantification of the chemotactic response (chemotaxis index) of (**g**) CD4 + T cells and **h** CD8 + T cells treated ±1 μM AMG487 or DMSO in response to control CM or TNF-α-CM from differentiated HPCs. Data is represented as mean ± SEM of three healthy donors. Statistical analysis: one-way ANOVA followed by Bonferroni’s multiple comparisons test. Source data are provided as a Source Data file. HPCs human hippocampal progenitor cells, TNF-α tumour necrosis factor alpha, CM conditioned media, CXCR3 C-X-C motif chemokine receptor 3, SEM standard error of the mean, DMSO dimethyl sulfoxide.

We generated conditioned media (CM) from TNF-α–treated HPCs as well as from HPC-derived cells differentiated under chronic TNF-α exposure, and assessed its ability to induce CXCR3-dependent migration of activated primary human CD4+ and CD8 + T cells (Fig. 5a–h). CM from both TNF-α–treated HPCs and their differentiated progeny significantly enhanced the chemotactic index of CD4+ and CD8 + T cells. This effect was significantly blunted by pre-treatment of the T cells with a CXCR3 antagonist, indicating that the migratory response was primarily CXCR3-dependent. These findings indicate that TNF-α–treated HPC cultures (during proliferation and differentiation) release CXCR3 ligands at concentrations sufficient to promote T cell chemotaxis. To infer the cellular source of these CXCR3 ligands, we re-analysed the scRNA-seq dataset and found that *CXCL9* was not detected, whereas *CXCL10* and *CXCL11* were enriched in an IFN-responsive glial/progenitor-like cluster (Supplementary Fig. 20), consistent with prior evidence that reactive astrocyte states are major producers of CXCL10 in the inflamed CNS^65^.

**Fig. 6:**
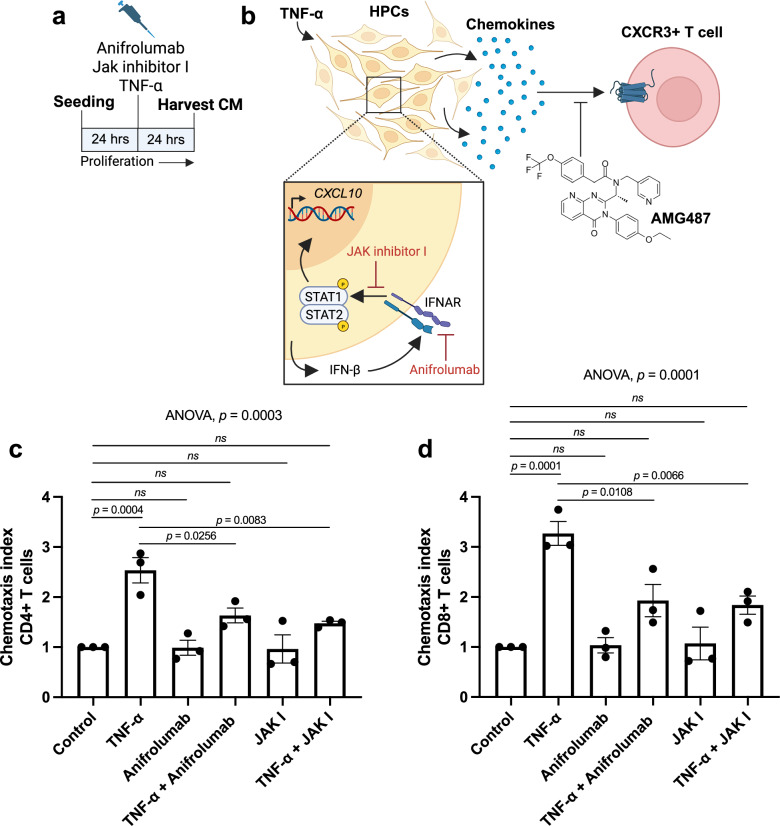
JAK and IFNAR signalling are indispensable for TNF-α-induced HPC chemokine secretion directing T cell chemotaxis. **a** Schematic overview of the experimental design for generating conditioned media (CM) from HPCs treated ± 1 ng/ml TNF-α, ±10 µg/ml anifrolumab (IFNAR antagonist), and ±0.16 nM JAK inhibitor I for 24 h. Created in BioRender. Nissen, T (2026) https://BioRender.com/p3voa5i. **b** The effect of IFNAR and JAK inhibition was evaluated for its ability to abrogate CXCR3-dependent T cell chemotaxis towards HPC TNF-α-CM. Created in BioRender. Nissen, T (2026) https://BioRender.com/na7h8qu. Quantification of the chemotactic response (chemotaxis index) of (**c**) CD4 + T cells and **d** CD8 + T cells in response to the CM generated as outlined in (**a**). Data is represented as mean ± SEM of three healthy donors. Statistical analysis: one-way ANOVA followed by Bonferroni’s multiple comparisons test. Source data are provided as a Source Data file. HPCs human hippocampal progenitor cells, TNF-α tumour necrosis factor alpha, CM conditioned media, IFNAR interferon alpha receptor, JAK Janus kinase, CXCR3 C-X-C motif chemokine receptor 3, SEM standard error of the mean.

As our data indicated TNF-α-induced *CXCL10* upregulation was mediated via the type I IFN autocrine/paracrine signalling loop, we tested whether IFNAR or JAK inhibition abrogated T cell chemotaxis in response to TNF-α-CM from HPCs (Fig. 6a, b). Pharmacological blockade of both IFNAR and JAK independently abrogated the chemotactic response of CD4 + T and CD8 + T cells towards chemokines secreted by TNF-α-stimulated HPCs (Fig. 6c, d). These results indicated that the secretion of CXCR3 ligands by HPCs in response to TNF-α was dependent on the autocrine/paracrine type I IFN signalling loop, consistent with the delayed and de novo protein synthesis-dependent upregulation of CXCL10 (Fig. 4d, e).

In addition to chemokine secretion, TNF-α upregulated the expression of adhesion molecules in HPCs implicated in T cell interactions including *ICAM1* and *VCAM1* (Supplementary Fig. 21). Notably, *ICAM1* expression was significantly increased after 3–6 h of TNF-α exposure and remained elevated on the HPC surface at 24 h post-treatment (Supplementary Fig. 22).

Together, these findings demonstrated that TNF-α stimulation of HPCs promoted CXCR3-dependent chemotaxis of primary human T cells in vitro through a type I IFN-mediated autocrine/paracrine loop. Furthermore, TNF-α enhanced HPC expression of adhesion molecules, potentially facilitating direct HPC–T cell interactions.

### Blocking IFNAR signalling rescues DCX⁺ neuroblast loss induced by chronic TNF-α

**Fig. 7:**
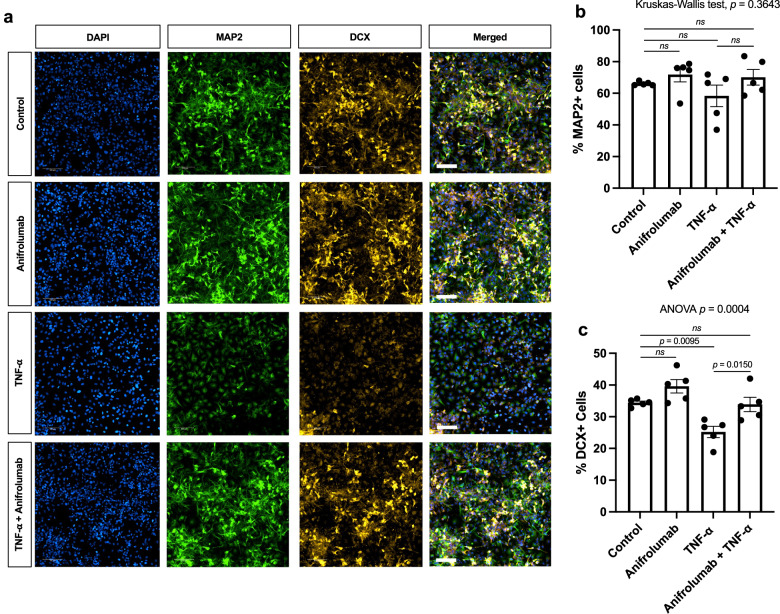
IFNAR antagonism rescues the chronic TNF-α-induced decrease in the percentage of DCX+ neuroblasts. **a** Representative images showing the expression of MAP2 (green) and DCX (orange) in seven days differentiated HPCs treated chronically ± 1 ng/ml TNF-α ±10 µg/ml anifrolumab. Scale bar represents 100 µm. **b**, **c** Quantification of the percentage of **b** MAP2+ cells and **c** DCX+ cells respectively based on (**a**). Data represent mean ± SEM from five independent experiments. Statistical analysis: **b** Kruskal-Wallis test followed by Dunn’s post hoc correction and **c** one-way ANOVA followed by Bonferroni’s multiple comparisons test. Source data are provided as a Source Data file. HPCs human hippocampal progenitor cells, TNF-α tumour necrosis factor alpha, IFNAR interferon alpha receptor, MAP2 microtubule-associated protein 2, DCX doublecortin, SEM standard error of the mean.

Given the prominent activation of STAT1/STAT2 downstream of TNF-α, and the established role of STAT1 as a negative regulator of hippocampal neurogenesis and cognition^52,66–68^, we hypothesised that chronic TNF-α may exert its antineurogenic effects via IFNAR-dependent type I interferon signalling. To test this, we administered the IFNAR-blocking antibody anifrolumab, which effectively suppressed TNF-α–induced ISG15 expression in differentiating HPCs (Supplementary Fig. 23), confirming inhibition of type I IFN signalling. As previously observed, chronic exposure to 1 ng/ml TNF-α selectively impaired the generation of DCX⁺ neuroblasts without affecting the proportion of MAP2⁺ neurons (Fig. 7a–c). Notably, IFNAR blockade fully rescued the loss of DCX⁺ neuroblasts, implicating autocrine/paracrine type I IFN signalling as a key mediator of TNF-α–driven neurogenic deficits. These findings are consistent with prior evidence that disrupting type I IFN pathways can restore hippocampal neurogenesis in ageing models^69^.

## Discussion

Emerging evidence implicates neuroinflammation, impaired AHN, and T cell accumulation within the hippocampal neurogenic niche as key features of ageing and neurodegenerative diseases^33–35^. However, the mechanistic links connecting these phenomena remain unclear. Here, we hypothesised that chronic TNF-α exposure functionally reprogrammes HPCs, diverting them from a neurogenic fate toward an immune-defensive phenotype, parallelling recent observations in olfactory NSCs^32^. This phenotypic switch may both impair AHN directly and promote T cell accumulation, which could further suppress neurogenesis. Together, these effects may contribute to cognitive deficits observed in ageing and neurodegeneration. Utilising a human in vitro model we demonstrated that chronic TNF-α suppresses hippocampal neurogenesis while promoting CXCL10 secretion. To investigate the molecular mechanisms underlying these observations, we performed scRNA sequencing, which unexpectedly revealed a type I IFN transcriptional signature in HPCs and their differentiating progeny. Mechanistically, we found that TNF-α engages a type I IFN signalling cascade in HPCs, driven by an autocrine/paracrine IFN-β. Moreover, this pathway suppressed neurogenesis and induced CXCR3 ligand secretion, promoting chemotaxis of activated primary human T cells. Our data uncovered a previously uncharacterised TNF-α-IFN-β-IFNAR-CXCL10 axis in HPCs and its role in reprogramming differentiating HPCs from a neurogenic to an immune-defensive state under chronic inflammatory conditions. Importantly, IFNAR antagonism restored TNF-α-induced neuroblasts loss and blocked T cell recruitment, identifying IFNAR signalling as a dual-action candidate therapeutic target for mitigating inflammation-induced neurogenic dysfunction.

TNF-α accumulation occurs in multiple pathophysiological contexts in which AHN is disrupted. During ageing, microglia adopt a chronic, low-grade inflammatory phenotype characterised by increased TNF-α expression^70,71^. CNS viral infections can similarly elevate TNF-α; for example, increased hippocampal TNF-α persists following West Nile virus infection, associated with reduced neurogenesis^22^. Activated microglia secrete TNF-α in chronic neurodegenerative diseases including AD^72^, Parkinson’s disease^73^, and multiple sclerosis^74^. Moreover, chronic peripheral inflammation can propagate to the brain, as demonstrated by increased hippocampal TNF-α and impaired neurogenesis during chronic intestinal inflammation^75^. Together, these findings highlight that CNS TNF-α accumulation is a common feature of diverse chronic inflammatory states, supporting the pathophysiological relevance of our chronic exposure model and identifying TNF-α as a potential convergent regulator of AHN across disease contexts.

Direct measurements of TNF-α in the human hippocampal interstitial space during neuroinflammatory conditions are lacking, which is a limitation when extrapolating in vitro dosing to the in vivo context. CSF TNF-α levels in neurodegenerative disease range from low to high pg/ml^76,77^. However, CSF represents a global compartment and may not reflect focal parenchymal concentrations in inflammatory niches. Activated microglia are a major source of TNF-α in the inflamed hippocampus^78,79^. In vitro studies of activated primary human microglia and human iPSC-derived microglia report TNF-α secretion ranging from high pg/ml to double-digit ng/ml depending on stimulus^80–88^. This suggests that ng/ml concentrations are pathophysiologically plausible in focal inflammatory microenvironments.

Of note, the TNF-α concentrations (0.1 and 1 ng/ml) used for the key experiments (Figs. 2–7) did not reduce total cell numbers or increase CC3+ apoptotic cells (Supplementary Fig. 7), suggesting that decreased neurogenesis and increased inflammatory signalling were unlikely to be driven by increased cell death. Consistent with this, scRNA-seq analysis showed that *TNFRSF1A* was detected across clusters, with the lowest fraction of *TNFRSF1A*+ cells in the neurogenic clusters (Supplementary Fig. 9). This pattern is consistent with TNF-α acting primarily on progenitor/early differentiation states, thereby biasing subsequent lineage decisions, and/or with paracrine type I IFN signalling from reactive glial cells driving the decreased neurogenesis. Moreover, apoptosis and programmed cell death signatures were not enriched in high-dose TNF-α-treated cells (Supplementary Fig. 10). Together, these observations argue against selective loss of neurogenic clusters via a TNFR1-high, apoptosis-prone subpopulation and support the conclusion that TNF-α reduces neurogenesis predominantly through transcriptional reprogramming. However, it remains possible that other CC3-independent mechanisms of cell death of differentiating neurogenic cells may be involved.

The antineurogenic effect of TNF-α we observed closely mirrors findings from primary NSCs and in vivo models^31,43,89,90^, reinforcing the validity of our human cellular system. Similarly, the TNF-α-induced impairment of neuronal morphological maturation^43,91^ and CXCL10 secretion align with previous reports in primary cells^45^. These parallels underscore the physiological relevance of our model and support its utility for mechanistic dissection of inflammation-driven neurogenic dysfunction. Future in vivo studies will be critical to extend these findings.

While type I IFN signalling is well characterised in infectious diseases^92^, its role in sterile inflammation remains less understood, but increasingly recognised^93,94^. Our findings identify a TNF-α-IFN-β-IFNAR axis in human HPCs, establishing TNF-α-driven type I IFN activation in a CNS-resident progenitor cell. Similar TNF-α-induced IFN-β responses have been reported in non-CNS cell types, requiring IRF1 and NF-κB^54–56,95^. Consistent with this, we showed that TNF-α induces *IFNB1* expression in HPCs via an NF-κB–dependent, protein synthesis–independent mechanism that may involve IRF1. Whether the TNF-α-IFN-β-IFNAR axis operates in CNS cell types beyond HPCs such as microglia, where IFN signatures have recently been identified across a broad range of sterile brain pathologies^96^ remains an important question for future investigations.

Given IFNAR’s ubiquitous expression^97^, HPC-derived IFN-β may act on surrounding niche cells, potentially amplifying local inflammatory crosstalk. To that end, our scRNA-seq data indicate that chronic TNF-α promotes a shift from homoeostatic astrocyte-like states toward inflammatory/reactive astroglial-like states, which may further amplify the niche inflammatory milieu through increased chemokine production (e.g., CXCL10), thereby reinforcing immune cell recruitment. This shift likely reflects transcriptional reprogramming, consistent with the extensive evidence that inflammatory cues such as TNF-α drive reactive astrocyte programmes^98^. Functionally, such a shift could further impair AHN by reducing homoeostatic astroglial support of neurogenesis. Similarly, IL-1β has been reported to reprogramme murine glial cells to adopt a proinflammatory, reactive state in the context of viral-driven inflammation, a process involving STAT3 signalling^99^. Consistent with this, our transcription factor analysis predicted STAT3 activity in high TNF-α-specific cell clusters (Fig. 3h), suggesting STAT3 may represent a shared regulatory node underlying cytokine-induced proinflammatory astrocyte states.

Although type I IFNs are traditionally considered as antiviral and neuroprotective, sustained or dysregulated signalling has been increasingly implicated in ageing^69,100^ and AD^101–103^. Elevated type I IFNs impair AHN^52,68,69^ and promote cognitive deficits^69,102,104^ both of which can be reversed through IFNAR antagonism^69,102^. These cognitive impairments are likely mediated, at least in part, by IFN-induced AHN dysfunction. Furthermore, type I IFNs can directly promote amyloidogenic, neurotoxic processes^105^. Our data support the pathogenic role for type I IFN signalling: notably, we found that TNF-α suppresses neurogenesis via type I IFN signalling and shifts differentiating progenitors towards an IFN-response/reactive astroglial-like state. This is consistent with recent evidence that Zika virus, which drives type I IFN signalling, promotes a molecular switch from neurogenesis towards astrogliogenesis^106^. Thus, type I IFNs may represent a converging downstream mechanism through which chronic inflammation impairs AHN and contributes to cognitive decline in ageing and neurodegeneration.

T cells infiltrate neurogenic niches in the context of ageing and AD^33–35,59–63,107^, and have been demonstrated to modulate brain ageing and neuroregeneration^108,109^. Understanding the mechanisms underlying their infiltration and interactions with brain resident cells is therefore critical. We found that TNF-α treatment induces HPC chemokine secretion promoting CXCR3-dependent chemotaxis of activated primary human T cells, suggesting hippocampal NSCs may directly recruit T cells during neuroinflammation, consistent with findings in murine olfactory NSCs^32^. This indicates a conserved mechanism across neurogenic niches and species, where chronic TNF-α reprogrammes NSCs towards immune cell recruitment. In addition to NSCs, microglial activation is reported to drive CXCL10-mediated CD8 + T cell recruitment and promote aging-related white matter degeneration^110^. Thus, multiple CNS cell types likely cooperate in recruiting T cells via CXCR3 ligands in ageing and neurodegeneration.

We demonstrated that IFNAR and JAK antagonism abolished T cell chemotaxis towards TNF-α-stimulated HPC-derived CM, demonstrating that autocrine/paracrine type I IFN signalling is the primary driver of CXCR3 ligand secretion. This mechanism parallels findings in a nephritis model, where TNFR2-induced IFN-β autocrine signalling promotes renal monocyte recruitment^55^. Of note, in the brain, type I IFN signalling in astrocytes promotes brain metastasis by enhancing monocytic myeloid cell recruitment^111^. Beyond promoting T cell chemotaxis, HPC-secreted CXCR3 ligands may exert direct deleterious effects within the neurogenic niche, as CXCR3 activation has been demonstrated to drive hippocampal neural hyperexcitability^103,112,113^.

As CXCL10 and CXCL11 are upregulated by IFN-γ^64^, IFN-γ-producing CD8 + T cells infiltrating ageing neurogenic niches^33^ may promote further CXCR3 ligand secretion from NSCs, potentially creating a feed-forward loop enhancing T cell recruitment. This may explain why CD8 + T cells have been found in close proximity to NSCs^33^. Increasing the number of CD8 + T cells in the hippocampus has been shown to decrease the number of proliferative progenitors and DCX+ newborn neurons^34^. This effect is likely mediated by IFN-γ secretion which has been shown to negatively affect NSCs^33^. Consistently, CXCL10 inhibition enhances neuroblast production^114^, possibly by reducing T cell infiltration. Thus, TNF-α may impair AHN both by driving antineurogenic type I IFN signalling in NSCs and recruiting CD8 + T cells that suppress neurogenesis via IFN-γ. Blocking adaptive immune cell infiltration into neurogenic niches may represent a therapeutic strategy for cognitive decline in ageing and neurodegeneration. Given the low abundance of NSCs, future in vivo studies are needed to determine their contribution to T cell recruitment in neuroinflammation.

The TNF-α-induced functional switch we observed in human HPCs —from a neurogenic to an immune responsive phenotype —may represent a protective adaptation to infection or injury. Since neurogenesis is energetically costly, redirecting cellular resources to mount an immune response could be advantageous for resolving the underlying pro-inflammatory challenge.

Collectively, our results provide insights into how chronic TNF-α changes the fate of human HPCs from neurogenesis towards immune functions, elucidating the molecular mechanism underlying this switch. Furthermore, this work offers an explanation for the emerging appearance of type I IFN signalling in ageing and neurodegeneration where TNF-α levels are elevated and highlights HPCs as potential direct participants in the progression of chronic inflammation.

## Methods

Experiments for this study complied with all relevant ethical regulations.

### Cellular model of hippocampal neurogenesis

We used the human hippocampal progenitor cell line (HPC0A07/03 A; ReNeuron LTD., Surrey, United Kingdom), derived from a first-trimester female foetus ethically approved under UK and US guidelines, as a cellular model for hippocampal neurogenesis as previously described^38,39,41,115^. The cell line was immortalised using the c*-mycER*^*TAM*^ gene to conditionally proliferate in presence of 100 nM 4-hydroxy-tamoxifen (4-OHT) (Sigma Aldrich, Cat No. H7904), 10 ng/mL human basic fibroblast growth factor (bFGF) (Peprotech, Cat No. 100-18B-500) and 20 ng/mL human epidermal growth factor (EGF) (Peprotech, Cat No. AF100-15–500) and spontaneously differentiate in their absence. The cells were cultured as previously described^116^ on laminin (Gibco, Cat No. 23017015) coated culture vessels (Nunclon, Denmark) at 37 °C, 5 % CO2.

### TNF-α-treatments

The cells were treated with 0.1, 1, or 10 ng/ml TNF-ɑ (Peprotech, Cat. No. 300-01 A) for the duration described in individual experiments.

### Immunocytochemistry

Cells were washed with PBS and fixed in 4% paraformaldehyde (in PBS) for 15 min at room temperature. Cells were permeabilised and blocked for 1 h at room temperature in blocking buffer: 0.3% Triton X-100 (Sigma, Cat No. X100) and 5% normal donkey serum (Bio-Rad, Cat No. C06SB) in PBS. For TNFR1 and TNFR2 staining, a non-permeabilising block (5% normal donkey serum in PBS) was used. Primary antibodies (Supplementary Table 1), diluted in blocking buffer, were applied overnight at 4 °C. The following day, cells were washed 3× with PBS and incubated with secondary antibodies (Supplementary Table 1) in blocking buffer for 2 h at room temperature, protected from light. 300 nM DAPI (Sigma, Cat No. 62248) in PBS was applied for 5 min at room temperature. Cells were then washed 4× with PBS and stored at 4 °C in PBS containing 0.05% sodium azide (Merck, Cat No. S2002).

### High-content imaging and analysis

Immunostained cells were imaged using the Opera Phenix Plus High-Content Screening System (PerkinElmer). Imaging was performed in a single focal plane using a 20× water-immersion objective. A minimum of 9 fields per well were acquired from 96-well plates and averaged to generate a mean per well. Channels were captured using the following excitation/emission settings: DAPI (ex 385 nm/em 425–475 nm), Alexa Fluor 488 (ex 488 nm/em 500–550 nm), and Alexa Fluor 555 (ex 561 nm/em 609–644 nm). A minimum of three technical replicates per condition were imaged and averaged for each biological replicate. Images were analysed using Harmony software (v4.9) (PerkinElmer). Briefly, cells were segmented using the *Find Nuclei* building block. Morphological parameters were calculated using *Calculate Morphological Properties*, and nuclei smaller than 50 μm^2^ or larger than 500 μm^2^ were excluded to remove apoptotic bodies and artefacts. Cells at image borders were also excluded. Cytoplasmic or nuclear signal intensity was quantified using *Find Cytoplasm*, *Find Morphological Properties*, and *Calculate Intensity Properties* building blocks. Positive cells were identified using the *Select Population* building block by applying a per-plate intensity threshold based on control wells. The percentage of positive cells was calculated using the *Define Results* building block.

### RNA extraction, cDNA synthesis, and RT-qPCR

HPCs (3.5 × 10⁵ cells per well) were seeded in 6-well plates coated with 20 μg/mL mouse laminin (Sigma, Cat No. L2020). Cells were treated the following day as indicated. For lysis, media were removed, wells were washed with PBS, and cells were lysed in 350 μL Buffer RLT Plus (Qiagen, Cat No. 74134) supplemented with 1:100 2-mercaptoethanol (Bio-Rad, Cat No. 1610710). Lysates were scraped, transferred to microcentrifuge tubes, vortexed, and stored at −80 °C. Total RNA was extracted using the RNeasy Plus Mini Kit (Qiagen, Cat No. 74134) following the manufacturer’s instructions, and RNA concentrations were measured using a DS-11FX+ (DeNovix). Extracted RNA (200 ng) was reverse transcribed using the High-Capacity cDNA Reverse Transcription Kit (Applied Biosystems, Cat No. 4368813) following the manufacturer’s protocol. The thermal cycler programme (Biometra Trio, Analytik Jena) used for cDNA synthesis is provided in Supplementary Table 2. qPCR reactions contained 5 μL cDNA (1:4 dilution in nuclease-free water), 10 μL SYBR Green Master Mix (PCR Biosystems, Cat No. PB20.11-05), 4 μL nuclease-free water, and 1 μL primer mix (10 pmol), in a total volume of 20 μL. Reactions were run in triplicate in 384-well plates (Applied Biosystems, Cat No. 4309849) on a QuantStudio 5 Real-Time PCR System (Applied Biosystems) using the cycling protocol described in Supplementary Table 3. Data were analysed with QuantStudio Design & Analysis Software (v1.5.1, Applied Biosystems), and relative gene expression was calculated using the 2^–ΔΔCt method with GAPDH as the reference gene. Primer sequences are provided in a Supplementary Data excel file.

### Western blotting

HPCs (3–6 × 10⁵ cells per well) were seeded in laminin-coated 6-well plates (Thermo Fisher Scientific, Cat No. 140675). Cells were treated the following day as indicated. Cells were washed with PBS and lysed in 150 μL 1 × Laemmli buffer (Bio-Rad, Cat. No. 1610747) diluted in PBS and supplemented with 355 mM β-mercaptoethanol (Bio-Rad, Cat. No. 1610710). Lysates were scraped, boiled for 5 min at 95 °C, and stored at –80 °C until analysis. Proteins were resolved by 10% SDS–PAGE and transferred to PVDF membranes (Thermo Fisher Scientific, Cat. No. 88518). Membranes were blocked for 1 h at room temperature in PBS containing 5% milk and 0.1% Tween-20, then washed 3× (10 min each) with PBS + 0.5% Tween-20. Primary antibodies (Supplementary Table 4) were applied overnight at 4 °C in blocking buffer. After washing, membranes were incubated with HRP-conjugated secondary antibodies for 1 h at room temperature, followed by 3× washes in PBS + 0.5% Tween-20. Detection was performed using Clarity Max ECL (Bio-Rad, Cat. No. 1705062) for STAT proteins and Clarity ECL (Bio-Rad, Cat. No. 1705061) for GAPDH, each for 4 min. Membranes were imaged on an ImageQuant 800 Imager (Amersham), and band intensities were quantified using Image Studio (v6.0.28, LI-COR BioSciences). Uncropped and unprocessed scans of blots are available in the Source Data file.

### Western blotting quantifications

Band intensities for phosphorylated and total STAT proteins, as well as GAPDH, were measured using Image Studio. Phosphorylated and total STAT signals were normalised to the corresponding GAPDH signal. The ratio of phosphorylated to total STAT protein was then calculated for each sample and expressed relative to control.

### Statistical analysis

Analyses were performed using GraphPad Prism (v10.4.1) and R (v4.3.2) with rstatix (v0.7.2). Independent experiments or donors were treated as the unit of replication for statistical testing. Normality was assessed by Q–Q plots and Shapiro–Wilk test, and variance homogeneity by Brown-Forsythe test. Results are reported as mean ± SEM, with statistical significance set at *p* < 0.05. The Source Data file contains the individual replicate values used to calculate summary statistics (mean ± SEM) and perform statistical testing. The specific test and sample size are stated in each figure legend. For normally distributed data, Student’s *t* test was used for two-group comparisons, and one-way ANOVA with Bonferroni’s post hoc test for >2 groups. All *t* tests were two-tailed. If variances were unequal, we applied Welch’s *t* test or Welch’s ANOVA with Games–Howell correction. Non-normally distributed data were analysed using the Kruskal–Wallis test with Dunn’s post hoc for multiple groups. No covariates were tested or adjusted for in the statistical analysis. Unless otherwise stated in the figure legends, measurements were obtained from distinct samples. Exact *P* values, test statistics (with degrees of freedom), effect sizes, and confidence intervals (where applicable) are reported in the Source Data file. Effect sizes were calculated as follows: for t-tests, η^2^ (reported by Prism as R^2^) was computed as η^2^ = t^2^/(t^2^ + df). For one-way ANOVA, ηp^2^ (reported by Prism as R^2^) was computed as SS_between/(SS_between + SS_within). For Welch’s ANOVA, ηp^2^ was computed from the Welch statistic as ηp^2^ = (DFn·W)/(DFn·W + DFd). For Kruskal–Wallis tests, η^2^(H) was computed as η^2^(H) = (H − k + 1)/(n − 1), where H is the Kruskal–Wallis statistic, k the number of groups, and n the total sample size.

### NF-kB nuclear translocation assay

HPCs (1.2 × 10^4^ cells per well) were seeded in laminin-coated 96-well plates. The following day cells were treated ± 10 ng/mL TNF-α and fixed after 0 min, 15 min, 30 min, and 3 h. Cells were immunostained for NF-κB p65 and counterstained with DAPI. High-content imaging was performed on an Opera Phenix (PerkinElmer), and images were analysed using Harmony software v4.9 (PerkinElmer). Nuclear and cytoplasmic NF-κB p65 signal intensities were measured, and the nuclear fraction of p65 fluorescence was calculated as described below:

$${Fraction}\,{of}\,{nuclear}\,{fluorescence}=\,\frac{{Nuclear}\,{intensity}}{{Nuclear}\,{intensity}+{cytosol}\,{intensity}}\,$$

### Cytokine and chemokine secretion analysis

HPCs (1.2 × 10⁵ cells) were seeded in laminin-coated 24-well plates (Thermo Fisher Scientific, Cat. No. 142475). The following day, the media was replaced with proliferation medium ± TNF-α. After 24 h and 48 h, supernatants were collected and stored at –80 °C. For differentiation, the cells cultured in proliferation media for 48 h were washed twice with differentiation medium for 30 min, then cultured in differentiation medium ± TNF-α. Supernatants were collected after 24 h, 48 h, and 7 d. IL-6 levels were quantified using a human IL-6 ELISA kit (BioLegend, Cat. No. 430504). A 13-plex panel of human pro-inflammatory chemokines (Supplementary Table 5) was analysed using a multiplex bead-based assay kit (BioLegend, Cat. No. 740985). Data was acquired using a FACSCanto II (BD Biosciences) flow cytometer and analysed using the LEGENDplex data analysis software suite (v8.0, BioLegend).

### Differentiation assay for immunocytochemistry

HPCs were seeded at 1.2 × 10^4^ cells per well in laminin-coated 96-well plates (Thermo Fisher Scientific, Cat. No. 140675) in proliferation medium containing 4-OHT, bFGF, and EGF. The following day, the medium was replaced with proliferation medium ± TNF-α. After 48 h, cells were washed twice with differentiation medium (no 4-OHT, bFGF, or EGF) for 30 min, then cultured in differentiation medium ± TNF-α for 7 d, with media changes every 48 h. In a subsequent experiment, cells were treated with ± 1 ng/mL TNF-α and ± 10 µg/mL anifrolumab.

### Neurite outgrowth analysis

Neurite outgrowth of DCX+ and MAP2+ cell populations was assessed using Harmony (v4.9) (PerkinElmer). Cells were identified as described in the High-Content Imaging and Analysis section. Neurites were detected using the ‘Find Neurites’ building block, and neurite morphology parameters, including the length of the longest neurite, were quantified.

### Cell culture and treatment for the single-cell RNA sequencing

#### Proliferation

HPCs were seeded into 20 μg/ml mouse laminin (Sigma, Cat. No. L2020) coated T175 flasks (Sigma, Cat. No. L2020) at 2.9 × 10^6^ cells for control or low TNF-α (0.1 ng/mL) conditions, and 5 × 10^6^ cells for high TNF-α (1 ng/mL) conditions. The following day, the media was replaced with proliferation medium ± TNF-α. The cells were harvested after 48 h.

#### Differentiation

HPCs were cultured as described above in proliferation medium ± TNF-α for 48 h. Next, the media was changed to differentiation media ± 0.1 ng/ml TNF-α or 1 ng/ml TNF-α. The HPCs were allowed to differentiate for seven or fourteen days. To mimic a chronic TNF-α-mediated proinflammatory environment the differentiation media was changed every 48 h to fresh differentiation media ± 0.1 ng/ml TNF-α or 1 ng/ml TNF-α. After seven or fourteen days of differentiation the cells were harvested.

### Harvesting cells for single-cell RNA sequencing

On the day of harvest, medium was removed and cells washed with pre-warmed DPBS (Gibco, Cat. No. 14190144). Cells were detached using pre-warmed Accutase (Gibco, Cat. No. A1110501) at 37 °C, 5% CO₂ for 5–10 min, harvested with pre-warmed DMEM/F12 (Gibco, Cat. No. 21331020), and centrifuged at 100 × *g* for 5 min. Pellets were gently resuspended in 1 mL of the warm proliferation or differentiation medium. Viability was confirmed at ≥85% using Trypan Blue (Thermo Fisher Scientific, Cat. No. 15250061,) and a hemocytometer before proceeding to antibody labelling.

### Cell surface staining with hashtag oligonucleotide antibodies

For 3′ scRNA-seq multiplexing on the 10x Genomics Chromium system, samples were labelled with TotalSeq™-B anti-human hashtag oligonucleotide (HTO) antibodies targeting CD298 and β2-microglobulin (BioLegend: Hashtag 1 Cat. No. 394631; Hashtag 2 Cat. No. 394633; Hashtag 3 Cat. No. 394635). Optimal antibody concentration (1:5000; 125 ng/mL) was determined by titrating PE-conjugated anti-CD298 (BioLegend, Cat. No. 341704) and anti-B2M (BioLegend, Cat. No. 316305) at a 1:1 equimolar ratio, followed by ICC to confirm 100% labelling with minimal background. Samples were individually labelled with HTOs prior to pooling at the sequencing facility.

1 × 10^6^ cells per sample were pelleted (300 × g, 5 min, 4 °C) and resuspended in 45 µL Cell Staining Buffer (CSB) (BioLegend, Cat. No. 420201). Cells were blocked with 5 µL TruStain FcX™ (BioLegend, Cat. No. 422301) for 10 min at 4 °C. TotalSeq™-B HTO antibodies were diluted 1:2500 in CSB and mixed 1:1 with blocked cells to reach a final concentration of 1:5000. Samples were incubated for 30 min at 4 °C. Cells were washed 3× in CSB (300 × *g*, 5 min, 4 °C) resuspending using wide-bore pipette tips (Starlab, Cat. No. E1011-9618). Final concentration was adjusted to 700–1200 cells/µL, assuming ~50% cell loss. Cell suspensions were filtered through 40 µm Flowmi™ Cell Strainers (Bel-Art, Cat. No. H13680-0040), and viability (>80%) was confirmed before proceeding to the 10x Genomics workflow. All steps were performed on ice.

### Single-cell capture, cDNA library preparation, and sequencing

HTO-labelled samples were pooled and processed by the Genomics Research Platform at Guy’s & St Thomas’s Hospital (London, UK). The differentiation samples (three pools) and the proliferation samples (one pool) were delivered to the facility on separate days. Equal cell numbers were combined, and 18,000 cells per pool were loaded onto separate lanes of the 10x Chromium X instrument (10x Genomics) for droplet-based single-cell capture. cDNA libraries were prepared using the Chromium Single Cell 3′ Reagent Kit v3 (10x Genomics, Cat. No. PN-1000268,). Libraries were sequenced on an Illumina NextSeq 2000 system using 50 bp paired-end reads.

### Read alignment and sample demultiplexing

FASTQ files from gene expression and HTO libraries were processed using Cell Ranger v7.1 (10x Genomics) with the cellranger multi pipeline. Data were imported into R (v4.3.2) using Seurat (v5.3.0) via the Read10X() function, returning UMI and HTO count matrices. Cells with barcodes present in both matrices were retained, and RNA counts were used to create a Seurat object (CreateSeuratObject()), with HTO data added as an independent assay (CreateAssayObject()). HTO data were normalised using centred log-ratio transformation and demultiplexed using HTODemux(assay = ‘HTO’) with default parameters. Only singlets with confident HTO classification were retained for downstream analysis.

### Quality control

QC was performed separately on merged Seurat objects for differentiation and proliferation samples. At the cell level, cells were excluded if they had >15% mitochondrial gene expression, a novelty score (log₁₀[genes/UMIs]) <0.8, <800 or >8000 genes, or <1000 or >45,000 transcripts. This yielded 22,133 high-quality cells (15,053 derived from differentiation samples; 7080 derived from proliferation samples). At the gene level, genes not expressed in any cells or expressed in ≤5 cells were excluded. Post-filtering, 26,288 genes were retained in the differentiation dataset and 21,951 in the proliferation dataset.

### Normalisation, dimensionality reduction, and clustering

The differentiation and proliferation Seurat objects were merged and normalised using sctransform (v2) from Seurat. Dimensionality reduction was performed using principal component analysis (PCA) on the top 3000 highly variable genes. An ElbowPlot indicated that the first 50 principal components captured the majority of variance in the dataset which was used for downstream analysis. We constructed a shared nearest neighbour graph using FindNeighbors(), tested clustering across resolutions from 0.1 to 1.2 (FindClusters()), and visualised cellular heterogeneity via UMAP (RunUMAP(dims = 1:50)), displayed with DimPlot(reduction = “umap”).

### Cluster annotation

Clusters were initially over-resolved using high-resolution settings and subsequently merged if they lacked distinct marker genes, were not biologically meaningful, or shared multiple canonical markers. This iterative approach—clustering, merging, and re-clustering—was guided by marker gene expression. Cluster markers were identified using the FindAllMarkers() function in Seurat, applying Wilcoxon rank-sum testing to compare each cluster against all others. Marker genes were required to be expressed in ≥25% of cells within the cluster and show positive differential expression (log₂ fold change ≥0.25). Ultimately, 15 clusters were identified and annotated based on culture condition, treatment, cell cycle stage, and canonical marker expression.

### Functional enrichment analysis

Gene Ontology (GO) over-representation analysis was performed using clusterProfiler^117^ (v4.14.6). For each comparison, the input gene list (top 100 genes ranked by log fold change) was tested using enrichGO() with Benjamini–Hochberg multiple testing correction. GO terms were retained using a raw enrichment *p* < 0.05 and a q-value < 0.2. Reported multiple testing-corrected enrichment values reflect overrepresentation within the queried gene sets and do not represent biological replication across independent samples (scRNA-seq libraries).

### Transcription factor activity inference

Transcription factor (TF) activity was inferred using decoupleR (v2.8)^118^. CollecTRI^119^ was retrieved via OmnipathR^120^ (v3.14.0). The univariate linear model generates a TF enrichment score by fitting a linear model of gene expression based on TF–gene interaction weights. The t-value of the slope (ULM score) indicates TF activity: positive scores denote activation and negative scores indicate inactivity.

### Gene module scoring for NSC-specific type I IFN response signature

Cells were scored using a 300-gene NSC-specific type I IFN response signature derived from murine SVZ NSCs treated ex vivo with IFN-β^51^. Average gene set expression per cell was calculated using the Seurat function AddModuleScore().

### Gene module scoring for human immature dentate granule cell signature

Cells were scored against the top 20 genes from a gene list identifying human immature dentate granule cells^9^. Average gene set expression per cell was calculated using the AddModuleScore() function.

### Conditioned media experiment for immunocytochemistry

To generate conditioned media (CM) 3 ×10^5^ HPCs were seeded into six well plates coated with 20 μg/ml mouse laminin (Sigma, Cat. No. L2020). The following day, the media was replaced with fresh media ±0.1 ng/ml, 1 ng/ml, or 10 ng/ml TNF-α. After 24 h, media was switched to control media without TNF-α and cells were cultured for an additional 24 h before CM collection. This approach captured HPC-secreted factors in response to TNF-α while eliminating residual TNF-α. To assess CM effects on IFN-regulated protein ISG15 expression, HPCs were seeded into laminin-coated 96-well plates. The next day, media was replaced with 70% CM mixed with 30% fresh media to maintain cell viability. After 48 h, cells were fixed, immunostained, and imaged to quantify the percentage of ISG15⁺ HPCs.

### Protein synthesis inhibition

To investigate the requirement of de novo protein synthesis in the TNF-α-induced upregulation of candidate genes, we utilised the global protein translation inhibitor, cycloheximide. The HPCs were treated as described in the results section with 10 μg/ml cycloheximide (Merck, Cat. No. 01810).

### Blocking NF-kB signalling

Cells were seeded in six-well plates coated with 20 μg/ml mouse laminin (Sigma, Cat. No. L2020). After 24 h, the media was replaced with media containing 10 μM JSH23 (Abcam, Cat. No. ab144824) dissolved in 100% dimethyl sulfoxide (DMSO) or with DMSO as control. The final DMSO concentration in both groups was 0.1%. Following a 30-min pre-treatment, 1 ng/ml TNF-α was added as indicated in the results section, and cells were harvested for gene expression analysis.

### Probing the dependency of IFNAR in TNF-α-induced type I IFN signalling

To assess IFNAR dependency in TNF-α-induced type I IFN signalling, HPCs (1.2 × 10^4^) were seeded in laminin-coated 96-well plates. The following day, cells were treated with or without 10 μg/ml anifrolumab (Bio X Cell, Cat. No. SIM0022), an anti-IFNAR1 antibody, or isotype control (Bio X Cell, Cat. No. SIM0014), and with or without 1 ng/ml TNF-α. After 24 h, cells were fixed and stained for ISG15, STAT1, and DAPI. High-content imaging quantified ISG15+ and STAT1+ cells as described.

### Blocking JAK activity

1.2 × 10^4^ HPCs were seeded in laminin-coated 96-well plates (Thermo Fisher Scientific, Cat. No. 167008). The following day, cells were treated with or without 16 nM JAK inhibitor I (Cayman Chemical, Cat. No. CAY15146) and with or without 1 ng/ml TNF-α. After 24 h, media was removed, cells fixed and stained, and high-content imaging was used to quantify ISG15+ HPCs.

### Evaluating cell surface protein expression with flow cytometry

Cells were cultured in laminin-coated T75 flasks (Sigma, Cat. No. L2020) and treated ± 1 ng/ml TNF-α for 24 h. Cells were detached with Accutase (Sigma, Cat. No. A1110501) for five min in the incubator, harvested, centrifuged, and resuspended in PBS. Cells were stained with Zombie NIR viability dye (BioLegend, Cat. No. 423105; 1:2000 in PBS) for 15 min at room temperature. Cells were washed and stained with antibodies (Supplementary Table 6) at 1:100 in staining buffer (PBS + 10 mM HEPES, 2 mM EDTA, 0.5% FCS) for 40 min at 4 °C. After washes, cells were resuspended in staining buffer. Data were acquired on a BD FACS Canto II using FACSDiva (v9.2) and analysed with FlowJo (v10.10) (BD Biosciences).

### Isolating peripheral blood mononuclear cells from leucocyte cones

Peripheral blood mononuclear cells (PBMCs) were isolated from leucocyte cones obtained from three healthy donors via NHS Blood and Transplant (NHSBT, Tooting, UK). Donors provided generic consent for research use. No identifiable donor information was available to the authors (sex or gender). Leucocyte cones were cleaned with ethanol and processed in a biosafety cabinet. Blood was drained into a 50 ml tube which was topped up to 50 ml with PBS containing 2% FCS. After gently mixing it was centrifuged at 800 × *g* for 10 min. The pellet was resuspended 1:1 in DPBS and layered onto 15 ml Ficoll-Paque PLUS (Cytiva, Cat. No. 17144002). After centrifugation at 450 × *g* for 30 min with minimal acceleration and no brake, the buffy coat was collected, washed twice with cold DPBS, and centrifuged at 400 × *g* for 10 min at 4 °C. A final centrifugation step at 200 × *g* for 10 min at 4 °C was performed to reduce platelet contamination. Cells were counted, resuspended in freezing media (90% FBS, 10% DMSO) at 360–600 × 10^6^ cells/ml, aliquoted into cryovials, frozen overnight at −80 °C in a Mr. Frosty container, and transferred to liquid nitrogen for long-term storage. Each vial contained 9–15 × 10^6^ cells.

### Chemotaxis assay

#### T cell activation and expansion protocol

PBMC cryovials were thawed and seeded in T75 flasks at 37 °C with 5% CO₂ in complete RPMI (10% FCS (Thermo Fisher Scientific, Cat. No. 26140079), 1% penicillin-streptomycin (Thermo Fisher Scientific, Cat. No. 15140122)). After 24 h, nonadherent lymphocytes were collected, centrifuged, and cultured in complete RPMI supplemented with 100 IU/ml recombinant human IL-2 (BioLegend, Cat. No. 689104) and 2.5 μg/ml phytohemagglutinin-L (PHA-L) (Thermo Fisher Scientific, Cat. No. 00-4977-93) in fresh T75 flasks. After 48 h, cells were harvested, washed, and resuspended in complete RPMI with 100 IU/ml IL-2. The cells were cultured for a total of 10 days and were resuspended in fresh complete RPMI media supplemented with 100 IU/ml IL-2 every other day.

#### Transwell chemotaxis assay

Transwell migration assays were performed using 96-well transwell plates with 5 μm pore membranes (Corning, Cat. No. 3387). Upper wells were coated overnight with 2% BSA in PBS to prevent cell adhesion and washed thrice with PBS the following day. Activated lymphocytes were harvested, washed, and resuspended at 4–5 × 10^6^ cells/ml in migration media (complete RPMI diluted 1:10 in neat RPMI), HPC proliferation media, or HPC differentiation media as appropriate. For CXCR3 dependency assays, cells were pretreated with 1 μM AMG487 (Cayman Chemical, Cat. No. 28416) or DMSO control for 30 min at 37 °C with rotation. 75 μl cell suspension were added to upper chambers. Lower chambers were filled with 235 μl media containing 100 ng/ml recombinant human CXCL10 (R&D Systems, Cat. No. 266-IP), TNF-α-conditioned media (CM) from proliferating or differentiating HPCs, or CM from HPCs treated ± TNF-α, JAK inhibitor I, or anifrolumab. After 3 h at 37 °C, migrated cells in the lower chamber were collected into 96-well plates for analysis.

#### Flow cytometry of migrated cells

Migrated cells were collected by spinning the plate at 2000 rpm for 1 min and washed with 200 μl DPBS. Cells were stained with Zombie NIR viability dye (BioLegend, Cat. No. 423105; 1:2000 in DPBS) for 15 min at room temperature in the dark, followed by quenching with 100 μl DPBS and centrifugation. For CXCR3 surface expression, cells were incubated with anti-CXCR3 antibody (1:100 in staining buffer: DPBS + 10 mM HEPES + 2 mM EDTA + 0.5% FCS) at 37 °C for 15 min. Next cells were stained with a master mix of antibodies (Supplementary Table 7; 1:50) for 40 min at 4 °C. Cells were washed twice with staining buffer and resuspended in 100 μl staining buffer. Data were acquired on a BD FACS Canto II with high-throughput sampler using FACSDiva v9.2 (BD Biosciences) with settings: flow rate 3.0, sample volume 50 μl, mixing volume 50 μl, mixing speed 200, two washes with 200 μl wash volume. Analysis was performed using FlowJo v10.10.

#### Generating conditioned media from HPCs for chemotaxis study

HPCs (3.5 × 10⁵) were seeded into six-well plates coated with 20 μg/ml mouse laminin (Sigma, Cat. No. L2020). After 24 h, media was replaced with proliferation media ± 1 ng/ml TNF-α. After 48 h, CM was collected and centrifuged at 1500 × g for 10 min at 4 °C. Supernatants were transferred to new tubes to remove cell debris and stored at –80 °C until use.

#### Generating conditioned media from differentiating HPCs

After 48 h of treatment in proliferation conditions as described above, media was replaced with differentiation media ± 1 ng/ml TNF-α. Cells were differentiated for 7 days, with media ± 1 ng/ml TNF-α refreshed every 48 h to simulate chronic inflammation. At the end of the differentiation period, CM was collected, centrifuged at 1500 × *g* for 10 min at 4 °C, and the supernatants were transferred to new tubes and stored at –80 °C for chemotaxis assays.

#### Generating conditioned media from HPCs blocking JAK activity or IFNAR

3.5 × 10⁵ HPCs were seeded in 6-well plates coated with 20 μg/ml mouse laminin (Sigma, Cat. No. L2020). The following day, media was replaced with fresh proliferation media with either DMSO (vehicle control), 1 ng/ml TNF-α, 10 μg/ml anifrolumab (anti-IFNAR1; Bio X Cell, Cat. No. SIM0022) ± 1 ng/ml TNF-α, or 0.15 nM JAK inhibitor I (Cayman Chemical, Cat. No. CAY15146) ± 1 ng/ml TNF-α. After 24 h, CM was collected, centrifuged at 1500 × g for 10 min at 4 °C, and supernatants were transferred to fresh tubes and stored at –80 °C for chemotaxis assays.

### Evaluating STING responsiveness

HPCs were stimulated ± 1 ng/ml TNF-α or ± 100 ng/ml diABZI (InvivoGen, cat. no. tlrl-diabzi-2) for 3 h before cells were harvested for RNA extraction, cDNA synthesis, and RT-qPCR as described above.

### Reporting summary

Further information on research design is available in the Nature Portfolio Reporting Summary linked to this article.

#### Ethics declaration

The authors declare that ethical standards prevailing at their institution have been observed throughout this study.

## Supplementary information

Supplementary Information

Description of Additional Supplementary Files

Supplementary Data 1

Reporting Summary

Transparent Peer Review file

## Source data

Source Data

## Data Availability

Source data are provided with this paper. Raw sequencing data have been deposited in the NCBI Sequence Read Archive under BioProject accession PRJNA1397568. Source data are provided with this paper.

## Code availability

The code used for the ScRNA-seq analysis is available at: https://github.com/thuretlabkcl/TNF-scRNAseq-neurogenesis.
