# Supplementary material for: TNF-α induces type I IFN signalling to suppress neurogenesis and recruit T cells
Source: Nat Commun. 2026 Jul 7;17:5287. doi: 10.1038/s41467-026-74104-x (PMC13342090; doi:10.1038/s41467-026-74104-x)
Supplement: Supplementary file 1 — Supplementary Information [file 41467_2026_74104_MOESM1_ESM.pdf]

Supplementary Information

TNF-α-induced type I IFN signalling decreases neurogenesis and drives T cell chemotaxis

Tinne Amalie Damgaard Nissen[1,2], Arishma Baig[1], Sahand Farmand[2], Daniel T. Rock[2], Sandra Shibu[2], Hyunah Lee[2], Lauren A. O'Neill[1], Vikki Houghton[2,3], Susan John[3], Linda S. Klavinskis[1]*, Sandrine Thuret[2]*

[1]Department of Infectious Diseases, School of Immunology & Microbial Sciences, King's College London, London SE1 9RT, UK
[2]Department of Basic and Clinical Neuroscience, Institute of Psychiatry, Psychology and Neuroscience, King's College London, London SE5 9RT, UK
[3]Peter Gorer Department of Immunobiology, King's College London, London SE1 9RT, UK

*Equally contributed and co-corresponding authors. Linda.Klavinskis@kcl.ac.uk and Sandrine.1.thuret@kcl.ac.uk

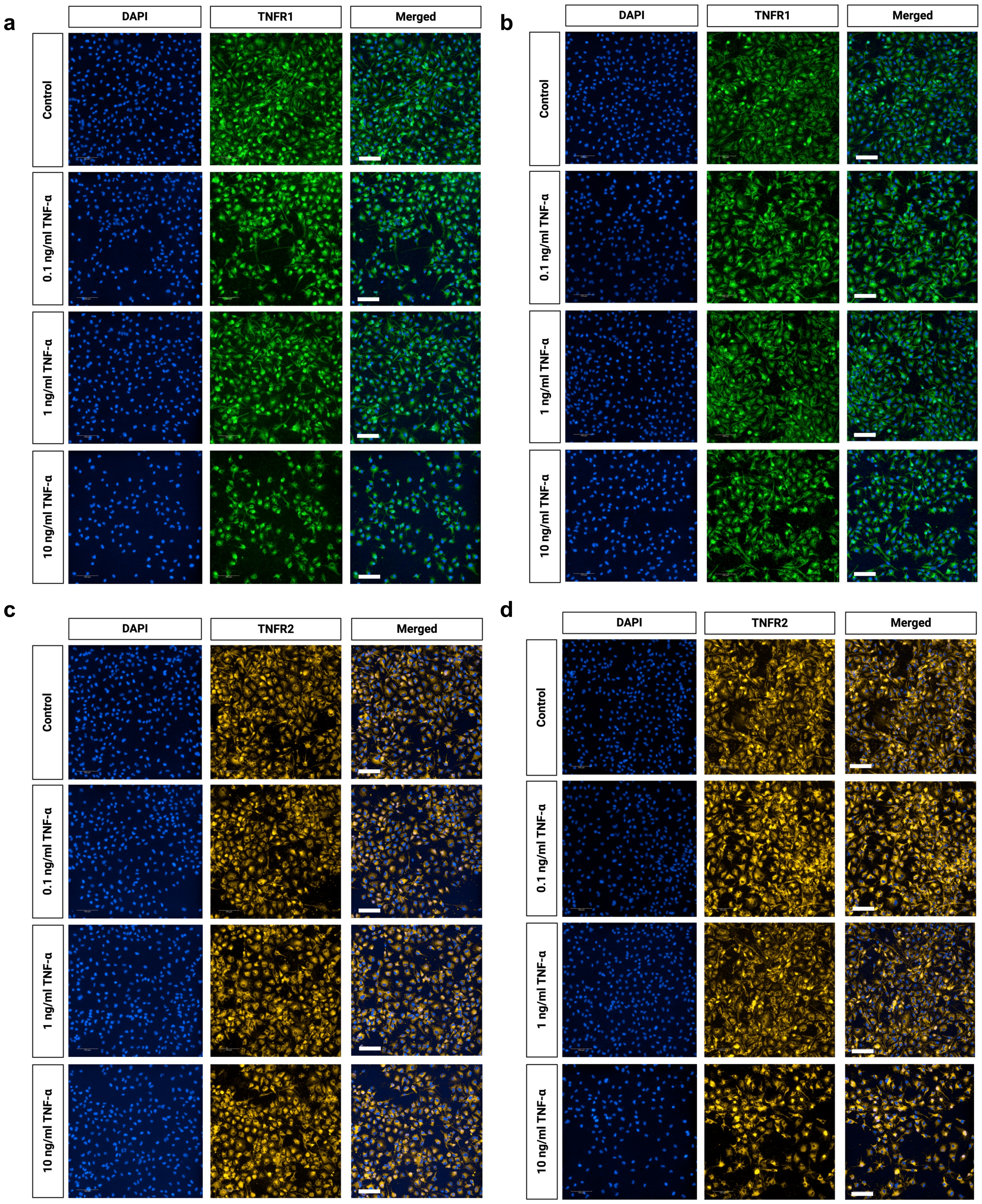

Supplementary Fig. 1 | TNFR expression on human HPCs treated ± TNF-α. a, and **b** Representative images of *n* = 3 independent experiments with similar results showing the expression of TNFR1 (green) on HPCs treated ± TNF-α for **a** 24 h or **b** 48 h. **c** and **d** Representative images of *n* = 3 independent experiments with similar results showing the expression of TNFR2 (orange) on HPCs treated ± TNF-α for **c** 24 h or **d** 48 h. Scale bar = 100 µm. HPCs, human hippocampal progenitor cells; TNF-α, tumour necrosis factor alpha;

TNFR1, tumour necrosis factor receptor 1; TNFR2, tumour necrosis factor receptor 2; DAPI, 4′,6-diamidino-2-phenylindole.

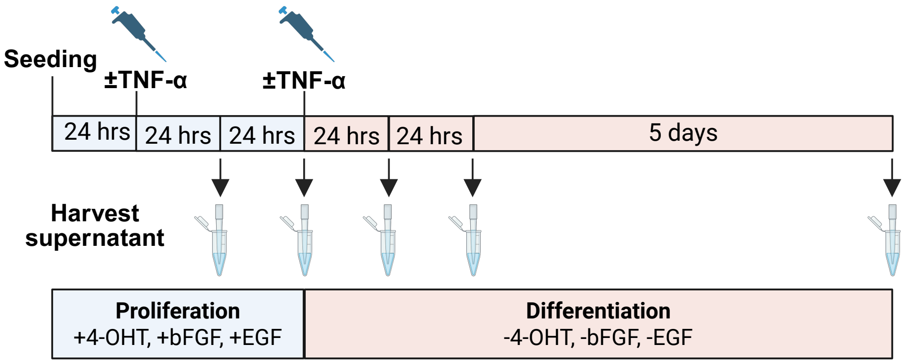

Supplementary Fig. 2 | Schematic overview of the experimental design for harvesting supernatant from HPCs and their differentiated progeny. The day following seeding, the proliferating HPCs were treated ± TNF-α (0.1 ng/ml, 1 ng/ml, or 10 ng/ml). After 24 h, the supernatant was harvested. After 48 h of treatment, the supernatant was harvested, and the media was changed to induce differentiation (removal of 4-OHT, bFGF, and EGF) ± TNF-α (0.1 ng/ml, 1 ng/ml, or 10 ng/ml). The supernatant of the differentiating cells was harvested after 24 h, 48 h, or 7 days of differentiation. Created in BioRender. Nissen, T. (2026) https://BioRender.com/56n7nj0. HPCs, human hippocampal progenitor cells; TNF-α, tumour necrosis factor alpha; 4-OHT, 4-hydroxytamoxifen; bFGF, basic fibroblast growth factor; EGF, epidermal growth factor.

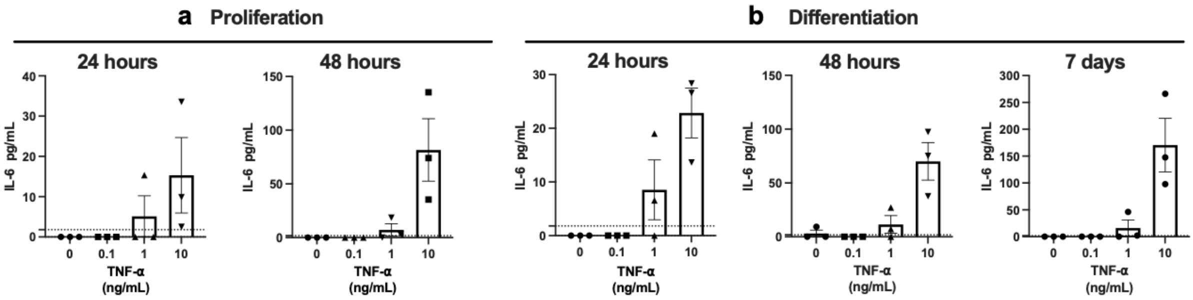

Supplementary Fig. 3 | ELISA measurement of IL-6 in the supernatant of HPCs and their differentiated progeny treated with TNF-α. a, Concentrations of IL-6 in the supernatant of proliferating HPCs 24 h and 48 h after TNF-α treatment. **b,** IL-6 in the supernatant of differentiating HPCs 24 h, 48 h, and 7 days after TNF-α treatment. Data is presented as mean ± SEM of *n* = 3 independent experiments. Source data are provided as a Source Data file. HPCs, human hippocampal progenitor cells; TNF-α, tumour necrosis factor alpha; IL-6, interleukin-6; SEM, standard error of the mean.

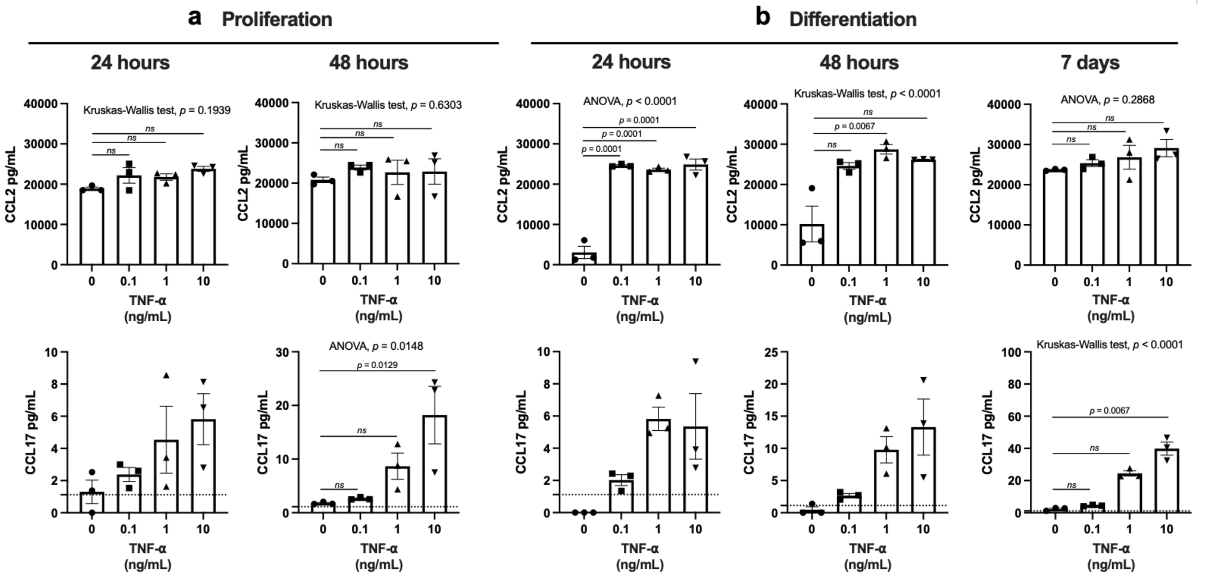

Supplementary Fig. 4 | CCL2 and CCL17 levels in the supernatant of HPCs and their differentiated progeny treated with TNF-α. a, Concentrations of CCL2 (upper panel) and CCL17 (lower panel) in the supernatant of proliferating HPCs 24 h and 48 h after TNF-α treatment. **b,** CCL2 (upper panel) and CCL17 (lower panel) in the supernatant of differentiating HPCs 24 h, 48 h, and 7 days after TNF-α treatment. Data is presented as mean ± SEM of n = 3 independent experiments. Statistical test: Kruskal-Wallis test followed by Dunn's post hoc correction, or one-way ANOVA followed by Bonferroni's multiple comparisons, as indicated above the graphs. Dashed lines mark the detection limit for CCL17 (1.12 pg/ml). Source data are provided as a Source Data file. HPCs, human hippocampal progenitor cells; TNF-α, tumour necrosis factor alpha; CCL2, C-C motif chemokine ligand 2; CCL17, C-C motif chemokine ligand 17; SEM, standard error of the mean

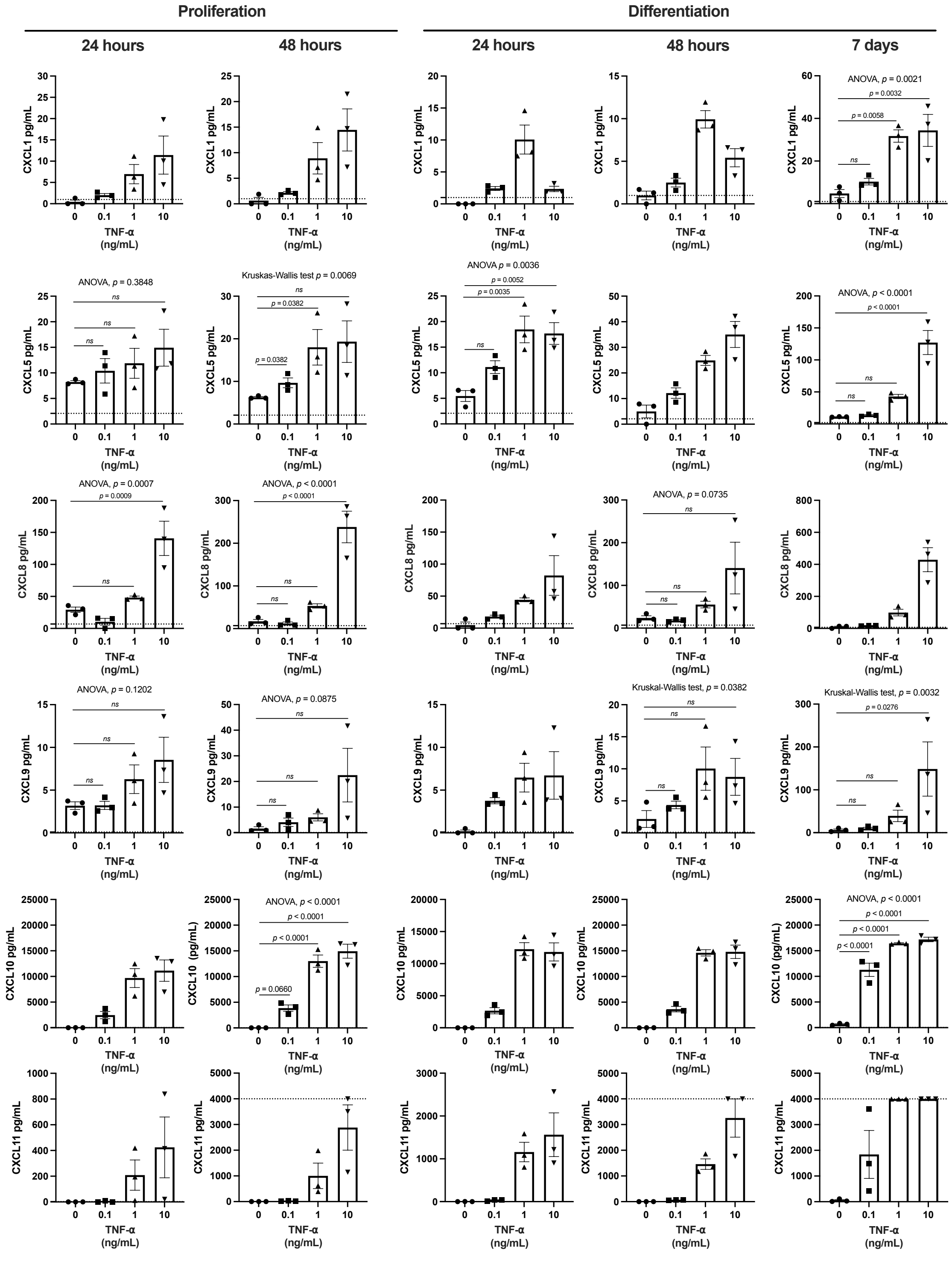

Supplementary Fig. 5 | CXC chemokine levels in the supernatant of HPCs and their differentiated progeny treated with TNF-α. a, Concentrations of CXCL1, CXCL5, CXCL8, CXCL9, CXCL10, and CXCL11 in the supernatant of proliferating HPCs 24 h and 48 h after TNF-α treatment. **b,** CXCL1, CXCL5, CXCL8, CXCL9, CXCL10, and CXCL11 levels in the supernatant of differentiating HPCs 24 h, 48 h, and 7 days after TNF-α treatment. Data is

presented as mean ± SEM of n = 3 independent experiments. Statistical test: Kruskal-Wallis test followed by Dunn's post hoc correction or one-way ANOVA followed by Bonferroni's multiple comparisons as indicated above the graphs. Dashed lines mark the detection limits (lower limit for CXCL1: <0.99 pg/ml, lower limit for CXCL5: <2.07 pg/ml, lower limit for CXCL8: <6.86 pg/ml, lower limit for CXCL9: <0.098 pg/ml, lower limit for CXCL10: <23.44 pg/ml, lower limit for CXCL11 <2.13 pg/ml, and upper limit for CXCL11 > 4000 pg/ml). For visualisation, data points below the detection limit were set to zero, and data points above the upper detection limit for CXCL11 were set to the limit of 4000 pg/ml. Source data are provided as a Source Data file. HPCs, human hippocampal progenitor cells; TNF-α, tumour necrosis factor alpha; CXC, C-X-C motif chemokine; CXCL1/5/8/9/10/11, C-X-C motif chemokine ligands 1, 5, 8, 9, 10 and 11; SEM, standard error of the mean.

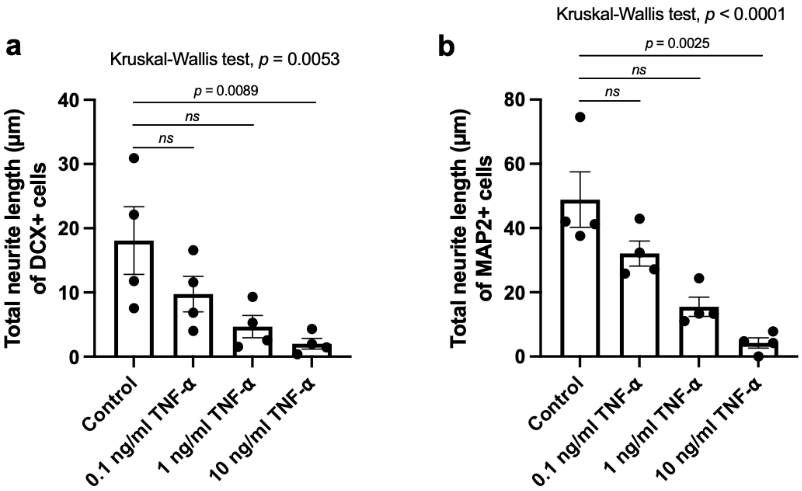

Supplementary Fig. 6 | Chronic TNF-α dose-dependently decreases the neurite length of DCX+ and MAP2+ differentiating HPCs. Quantification of the total neurite length (µm) of **a** DCX+ cells and **b** MAP2+ cells after seven days of differentiation ± chronic treatment with 0.1, 1, or 10 ng/ml TNF-α. Data represent mean ± SEM from n = 4 independent experiments. Statistical analysis: Kruskal-Wallis test followed by Dunn's post hoc correction**.** Source data are provided as a Source Data file. HPCs, human hippocampal progenitor cells; TNF-α, tumour necrosis factor alpha; DCX, doublecortin; MAP2, microtubule-associated protein 2; SEM, standard error of the mean.

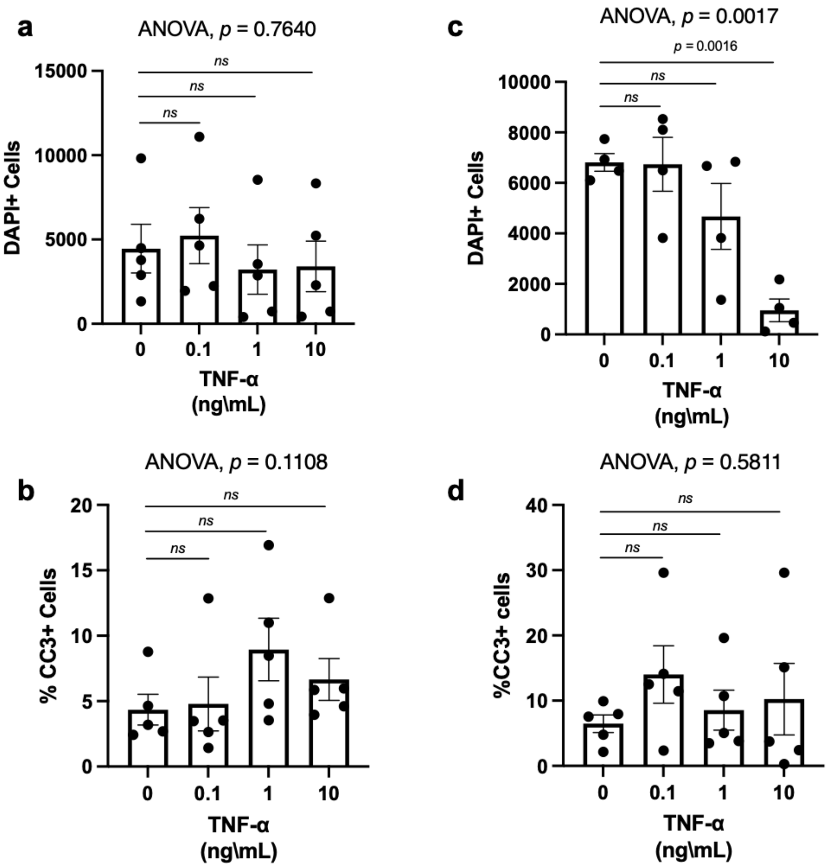

Supplementary Fig. 7 | TNF-α does not increase the percentage of CC3+ cells in proliferating and differentiating HPCs. **a** and **b** Number of DAPI+ cells and percentage of CC3+ proliferating HPCs after 24 h treatment ± 0.1, 1, or 10 ng/ml TNF-α. **c** and **d** Number of DAPI+ cells and percentage of CC3+ cells kept in proliferation conditions for 48 h ± 0.1, 1, or 10 ng/ml TNF-α followed by seven days of differentiation during chronic exposure to ± 0.1, 1, or 10 ng/ml TNF-α. Data represent mean ± SEM of n = 5 independent experiments for panel a, b and d, and n = 4 independent experiments for panel c. Statistical analysis: panels a, c, d, were analysed using one-way ANOVA followed by Bonferroni's multiple comparisons; panel b was analysed using Kruskal-Wallis test followed by Dunn's post hoc correction. Source data are provided as a Source Data file. HPCs, human hippocampal progenitor cells; TNF-α, tumour necrosis factor alpha; CC3, cleaved caspase-3; DAPI, 4′,6-diamidino-2-phenylindole; SEM, standard error of the mean.

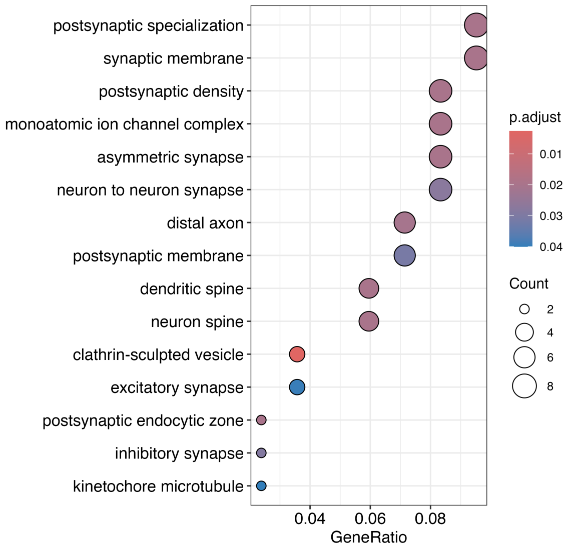

Supplementary Fig. 8 | Cellular components enriched in the immature neuron-like clusters. Dot plot showing GO cellular component enrichment based on the top 100 genes positively defining the immature neuron-like cluster. scRNA-seq was performed with *n* = 1 10x library per condition/timepoint. GO, Gene Ontology.

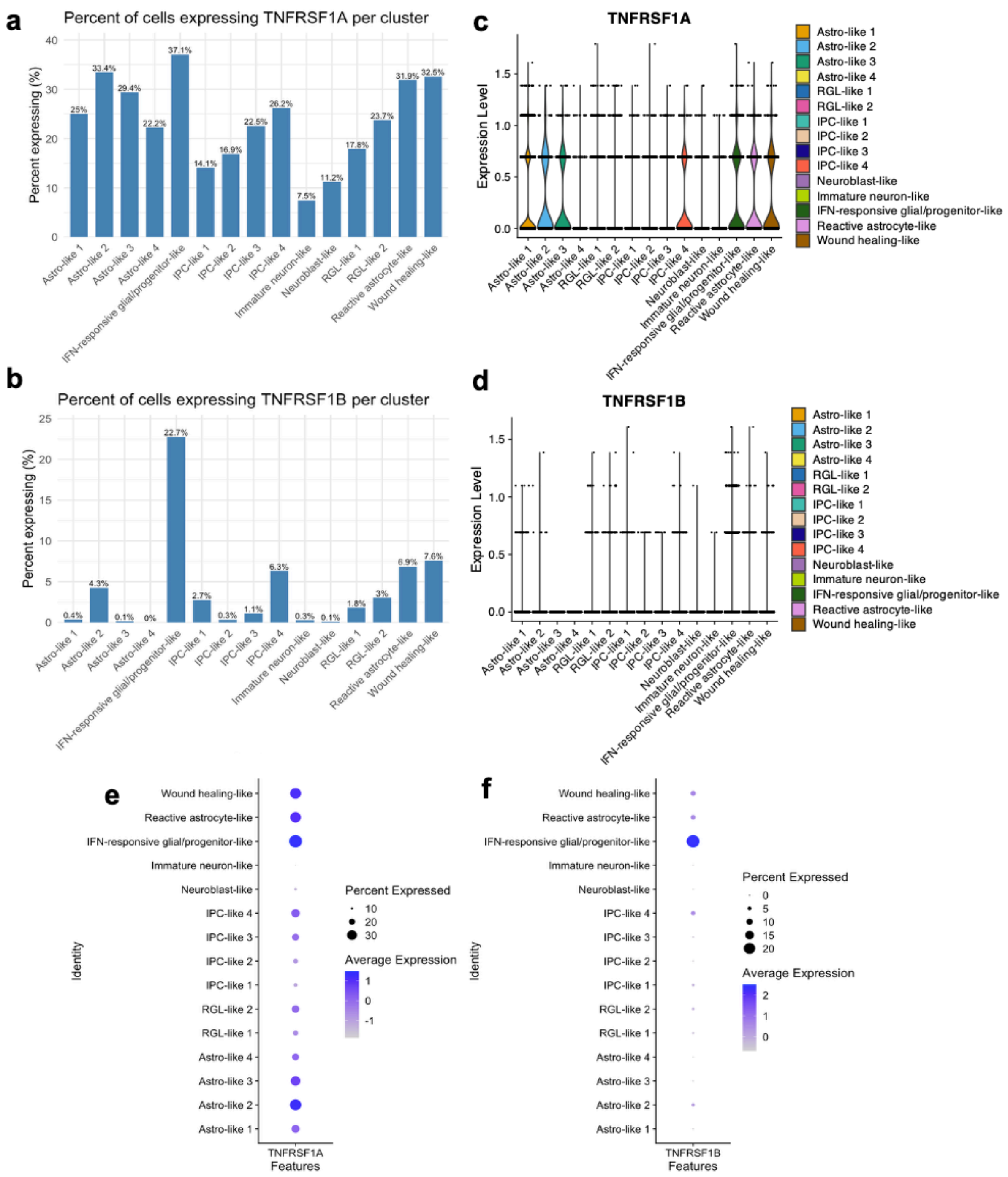

Supplementary Data Fig. 9 | *TNFRSF1A* and *TNFRSF1B* expression across clusters. a and **b** Bar plots showing the percentage of cells expressing *TNFRSF1A* (encoding TNFR1) and *TNFRSF1B* (encoding TNFR2) per cluster. Source data are provided as a Source Data file. **c** and **d** Violin plots showing the expression of *TNFRSF1A* (encoding TNFR1) and *TNFRSF1B* (encoding TNFR2) per cluster. **e** and **f** Dot plots showing the expression of *TNFRSF1A* (encoding TNFR1) and *TNFRSF1B* (encoding TNFR2) per cluster. scRNA-seq was performed with n = 1 10x library per condition/timepoint. TNFR1, tumour necrosis factor receptor 1; TNFR2, tumour necrosis factor receptor 2.

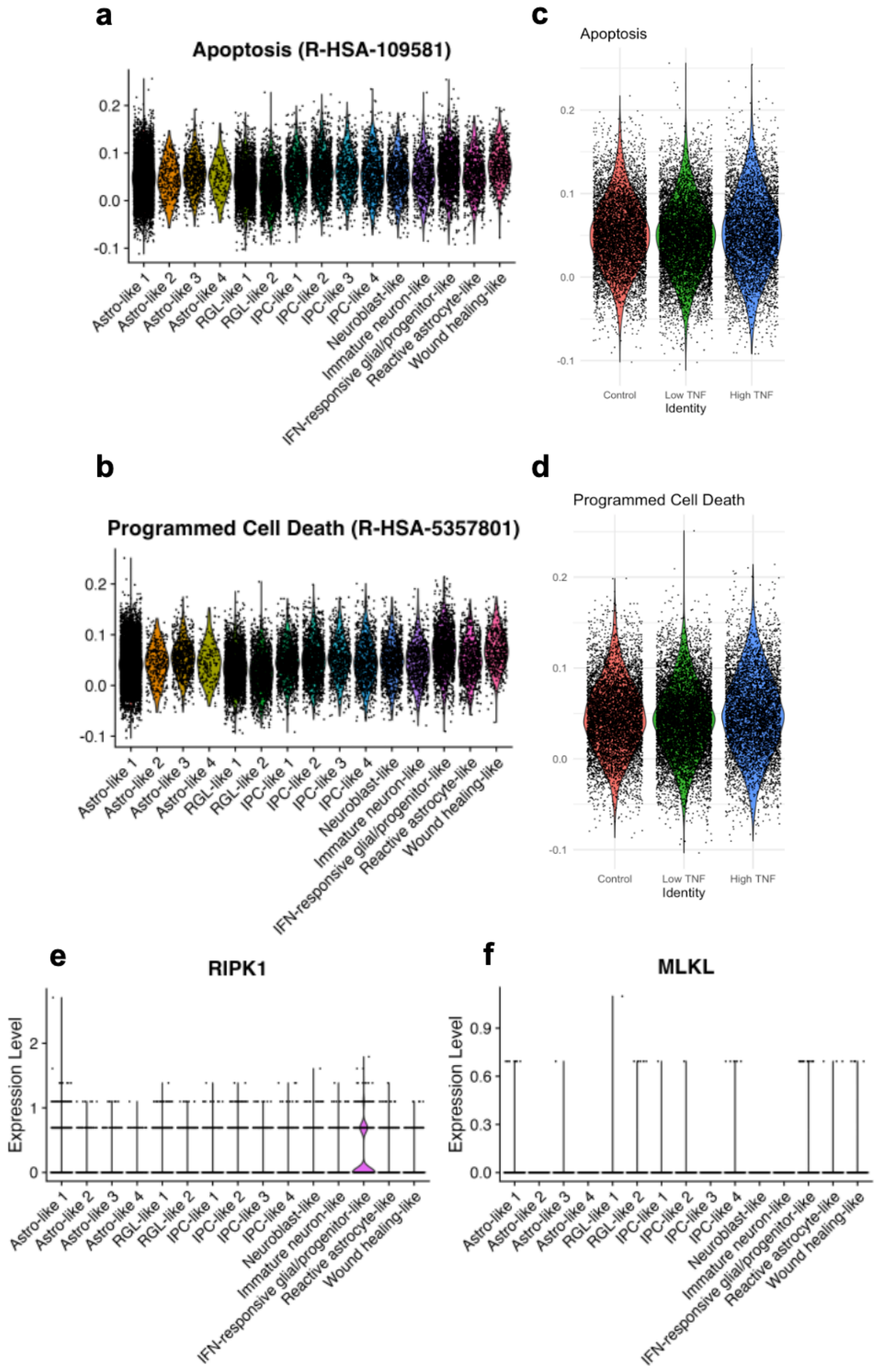

Supplementary Fig. 10 | Cell death gene signatures across clusters and treatment groups. a and **b** Violin plots showing clusters scored against an apoptosis gene signature (R-

HAS-109581) and a programmed cell death gene signature (R-HAS-5357801). **c** and **d** Violin plots showing treatment groups scored against an apoptosis gene signature (R-HAS-109581) and a programmed cell death gene signature (R-HAS-5357801) (all timepoints pooled). **e** and **f** Violin plots showing the expression of core necroptosis machinery genes *RIPK1* and *MLKL*. scRNA-seq was performed with *n* = 1 10x library per condition/timepoint. RIPK1, receptor-interacting serine/threonine-protein kinase 1; MLKL, mixed lineage kinase domain-like pseudokinase.

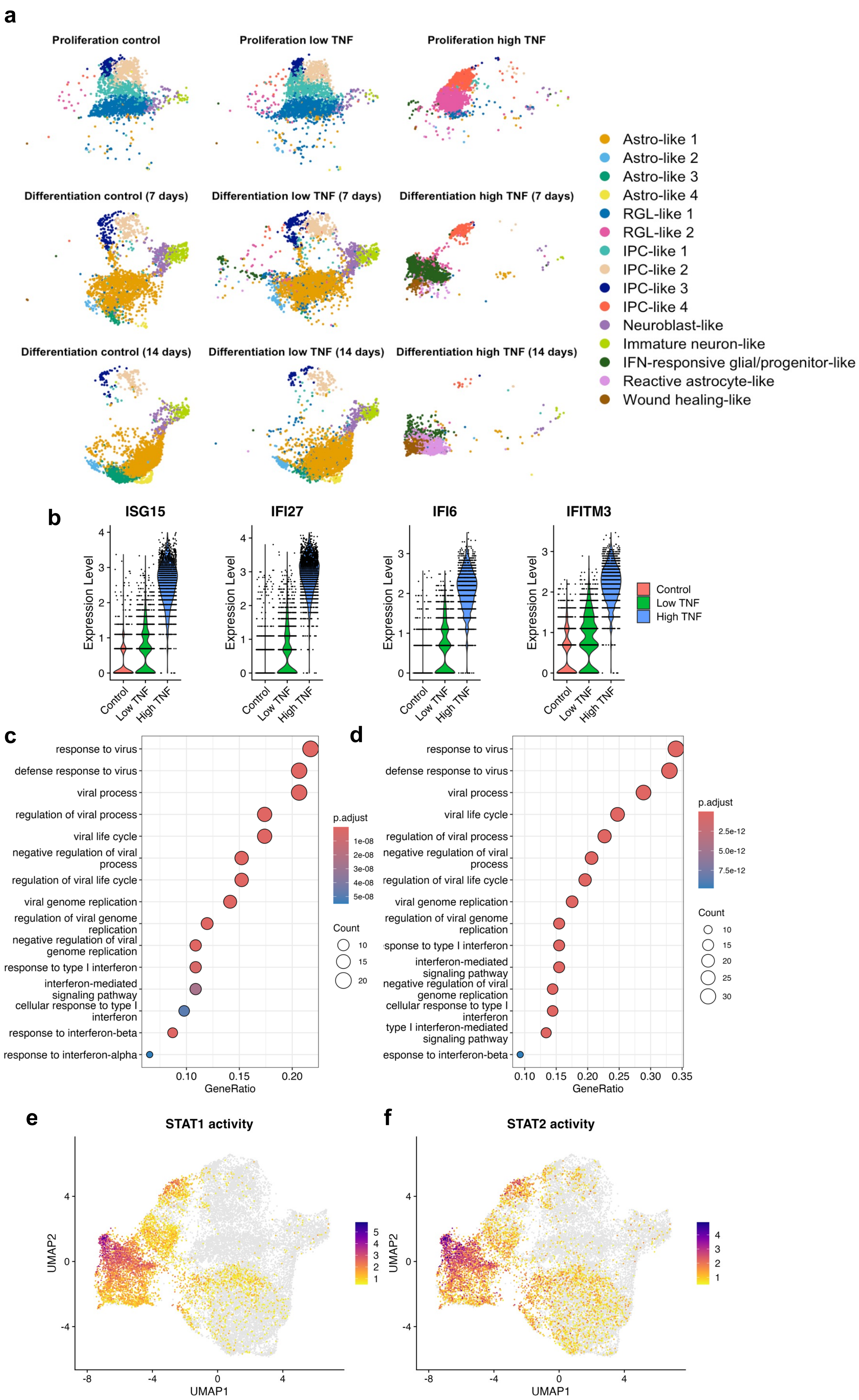
a
Proliferation control
Proliferation low TNF
Proliferation high TNF
Differentiation control (7 days)
Differentiation low TNF (7 days)
Differentiation high TNF (7 days)
Differentiation control (14 days)
Differentiation low TNF (14 days)
Differentiation high TNF (14 days)
Astro-like 1
Astro-like 2
Astro-like 3
Astro-like 4
RGL-like 1
RGL-like 2
IPC-like 1
IPC-like 2
IPC-like 3
IPC-like 4
Neuroblast-like
Immature neuron-like
IFN-responsive glial/progenitor-like
Reactive astrocyte-like
Wound healing-like
b
ISG15
IFI27
IFI6
IFITM3
Expression Level
Control
Low TNF
High TNF
c
response to virus
defense response to virus
viral process
regulation of viral process
viral life cycle
negative regulation of viral process
regulation of viral life cycle
viral genome replication
regulation of viral genome replication
negative regulation of viral genome replication
response to type I interferon
interferon-mediated signaling pathway
cellular response to type I interferon
response to interferon-beta
response to interferon-alpha
GeneRatio
p.adjust
1e-08
2e-08
3e-08
4e-08
5e-08
Count
10
15
20
d
response to virus
defense response to virus
viral process
viral life cycle
regulation of viral process
negative regulation of viral process
regulation of viral life cycle
viral genome replication
regulation of viral genome replication
sponse to type I interferon
interferon-mediated signaling pathway
negative regulation of viral genome replication
cellular response to type I interferon
type I interferon-mediated signaling pathway
esponse to interferon-beta
GeneRatio
p.adjust
2.5e-12
5.0e-12
7.5e-12
Count
10
15
20
25
30
e
STAT1 activity
f
STAT2 activity
UMAP1
UMAP2

Supplementary Fig. 11 | TNF-α drives the upregulation of type I IFN genes in HPCs. a, UMAP plot showing transcriptional clustering of single cells split by samples. Colours indicate the 15 cell clusters identified. **b**, Violin plots showing dose-dependent expression of the type I IFN stimulated genes *ISG15*, *IFI27*, *IFI6*, and *IFITM3* in the HPCs treated for 48 hrs with vehicle (red), 0.1 ng/ml (low, green), or 1 ng/ml (high, blue) TNF-α. **c**, Dot plot showing gene ontology (GO) enrichment analysis of biological processes terms based on the top 100 genes upregulated genes in the RGL-like 1 cluster as compared to the RGL-like 2 cluster. **d**, Dot plot showing GO enrichment analysis of biological processes terms based on the top 100 genes upregulated genes in the IPC-like 4 cluster as compared to the IPC-like 1, IPC-like 2, and IPC-like 3 clusters. **e**, UMAP plot showing the predicted transcription factor activity of STAT1. **f**, UMAP plot showing the predicted transcription factor activity of STAT2. scRNA-seq was performed with *n* = 1 10x library per condition/timepoint. HPCs, human hippocampal progenitor cells; TNF-α, tumour necrosis factor alpha; IFN, interferon; UMAP, uniform manifold approximation and projection; GO, Gene Ontology; RGL, radial glia-like; IPC, intermediate progenitor cell; ISG15, interferon-stimulated gene 15; IFI27, interferon-inducible protein 27; IFI6, interferon-inducible protein 6; IFITM3, interferon-induced transmembrane protein 3; STAT1, signal transducer and activator of transcription 1; STAT2, signal transducer and activator of transcription 2.

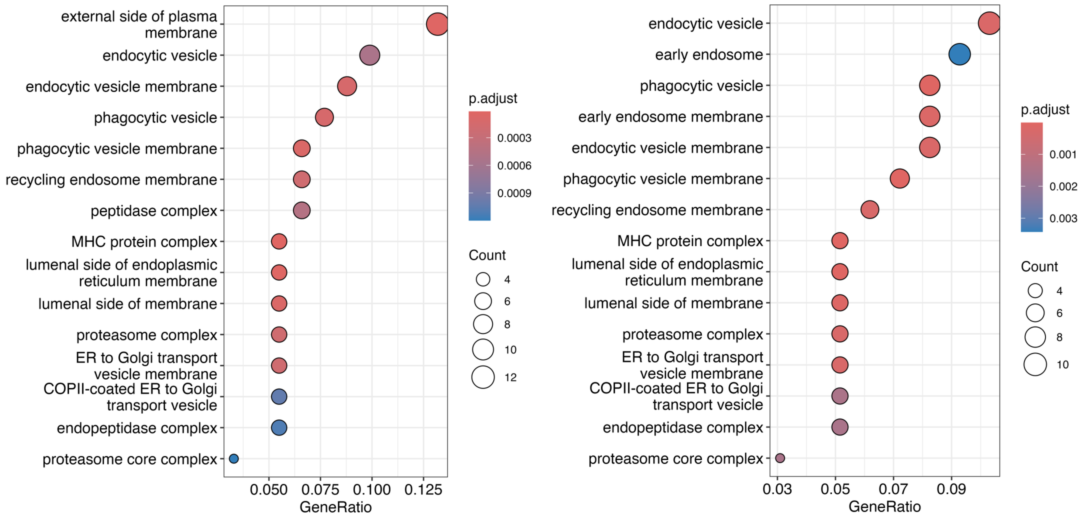

Supplementary Fig. 12 | Immune-related cellular components enriched in high-dose TNF-α-specific RGL-like and IPC-like clusters. Dot plot showing Gene Ontology (Cellular Component) enrichment based on the top 100 genes upregulated in RGL-like 2 cluster as compared to the RGL-like 1 cluster (left). Dot plot showing Gene Ontology (Cellular Component) enrichment based on the top 100 upregulated genes in the IPC-like 4 cluster as compared to the IPC-like 1, IPC-like 2, and IPC-like 3 clusters (right). scRNA-seq was

performed with n = 1 10x library per condition/timepoint. RGL, radial glia-like; IPC, intermediate progenitor cell.

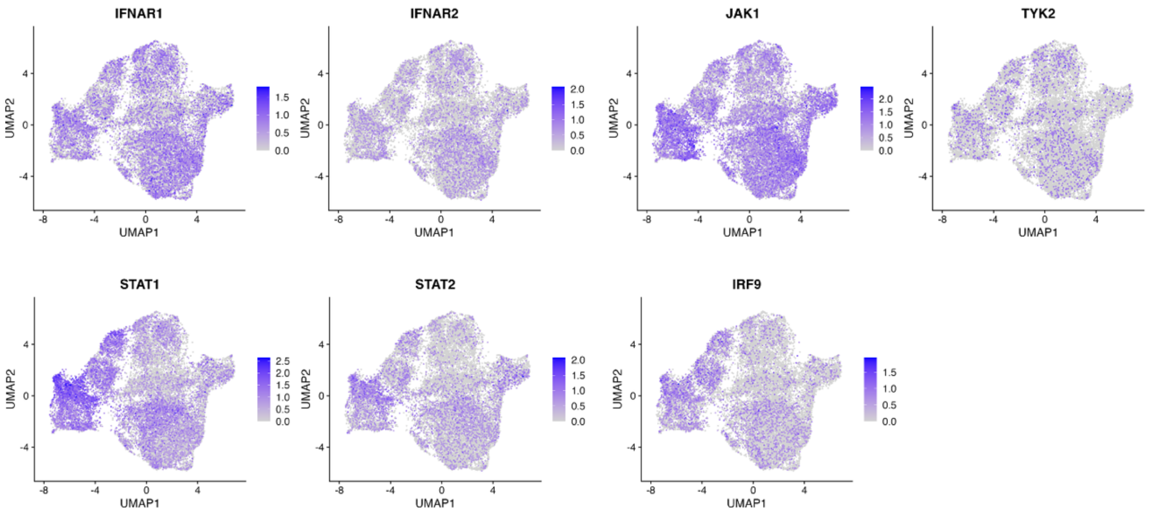

Supplementary Fig. 13 | Molecular machinery for type I interferon signalling. UMAP plots showing the expression of key genes required for type I interferon signalling: *IFNAR1*, *IFNAR2*, *JAK1*, *TYK2*, *STAT1*, *STAT2*, and *IRF9*. scRNA-seq was performed with n = 1 10x library per condition/timepoint. UMAP, uniform manifold approximation and projection; IFNAR1, interferon alpha and beta receptor subunit 1; IFNAR2, interferon alpha and beta receptor subunit 2; JAK1, Janus kinase 1; TYK2, tyrosine kinase 2; STAT1, signal transducer and activator of transcription 1; STAT2, signal transducer and activator of transcription 2; IRF9, interferon regulatory factor 9.

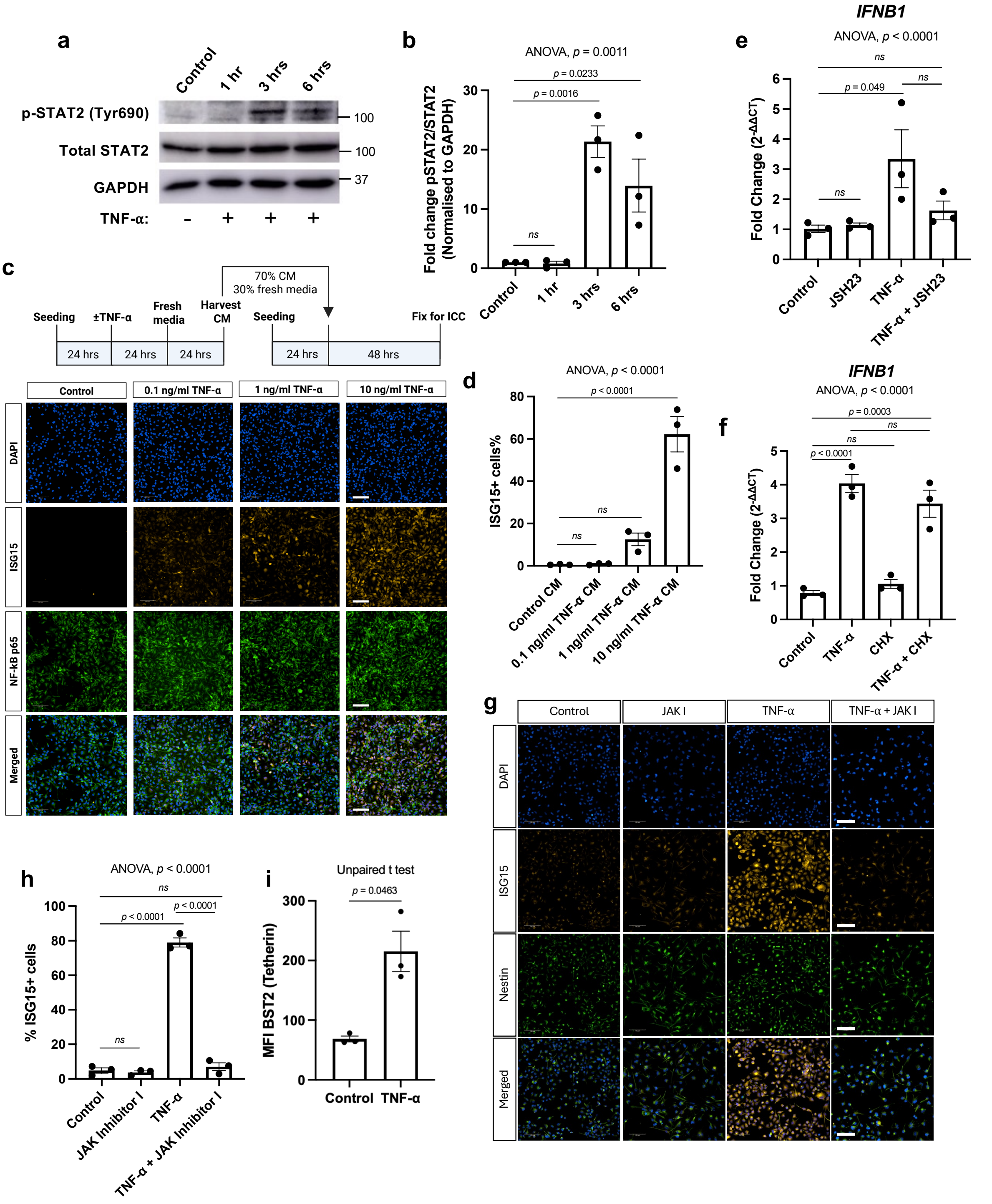

Supplementary Fig 14 | TNF-α drives type I IFN signalling in human HPCs via autocrine/paracrine signalling via IFNAR. **a**, Representative western blots of *n* = 3 independent experiments with similar results showing the activation of STAT2 in HPCs treated with 1 ng/ml TNF-α for 1 h, 3 h, 6 h or 12 h. Cell lysates were probed for phosphorylated STAT2 (p-STAT2, Tyr690), total STAT2, and GAPDH as a loading control. **b**, Quantification of STAT2 activation in human hippocampal progenitor cells from **a**. p-STAT2 and STAT2 intensities were normalised to GAPDH intensity before calculating the fold change p-

STAT2/STAT2 relative to control. Data represent mean ± SEM of *n* = 3 independent experiments. Statistical analysis: one-way ANOVA followed by Bonferroni's multiple comparisons test. **c**, Representative images of *n* = 3 independent experiments with similar results showing the expression of ISG15 (orange) on HPCs. Scale bar = 100 µm. HPCs treated for 48 hrs with conditioned media (CM) from other HPCs treated ± TNF-α for 24 hrs followed by 24 hrs treatment with media. Created in BioRender. Nissen, T. (2026) https://BioRender.com/l4z4pum. **d**, Quantification of the percentage of ISG15+ cells based on **c**. Data represent mean ± SEM from *n* = 3 independent experiments. Statistical analysis: one-way ANOVA followed by Bonferroni's multiple comparisons test. **e**, RT-qPCR data showing the relative gene expression of *IFNB1* in HPCs pre-treated with 10 µM NF-kB inhibitor JSH23 or DMSO, followed by three hours of treatment ± 1 ng/ml TNF-α. Data represent as mean ± SEM of *n* = 3 independent experiments. Statistical analysis: one-way ANOVA followed by Bonferroni's multiple comparisons test. **f**, RT-qPCR data showing the relative gene expression of *IFNB1* in HPCs pre-treated with 10 µg/ml cycloheximide (CHX) or DMSO followed by three hours of treatment ± 1 ng/ml TNF-α. Data represent as mean ± SEM of *n* = 3 independent experiments. Statistical analysis: one-way ANOVA followed by Bonferroni's multiple comparisons test. **g**, Representative images of *n* = 3 independent experiments with similar results showing the expression of ISG15 (orange) in HPCs. Scale bar = 100 µm. HPCs were treated for 24 hrs with 16 nM JAK inhibitor I or DMSO, ± 1 ng/ml TNF-α. **h**, Quantification of the percentage of ISG15+ cells based on **g**. Data represent mean ± SEM from *n* = 3 independent experiments. Statistical analysis: one-way ANOVA followed by Bonferroni's multiple comparisons test. **i**, Cell surface expression of IFN-regulated BST2 (tetherin) quantified by measuring the mean fluorescence intensity (MFI) on HPCs treated 24 hrs ± 1 ng/ml TNF-α using flow cytometry. Data represent mean ± SEM from *n* = 3 independent experiments. Statistical analysis: unpaired, two-tailed, t-test with Welch's correction. Source data are provided as a Source Data file. HPCs, human hippocampal progenitor cells; TNF-α, tumour necrosis factor alpha; IFN, interferon; IFNAR, interferon alpha/beta receptor; STAT2, signal transducer and activator of transcription 2; GAPDH, glyceraldehyde 3-phosphate dehydrogenase; CM, conditioned media; RT–qPCR, reverse transcription quantitative PCR; CHX, cycloheximide; MFI, mean fluorescence intensity; SEM, standard error of the mean.

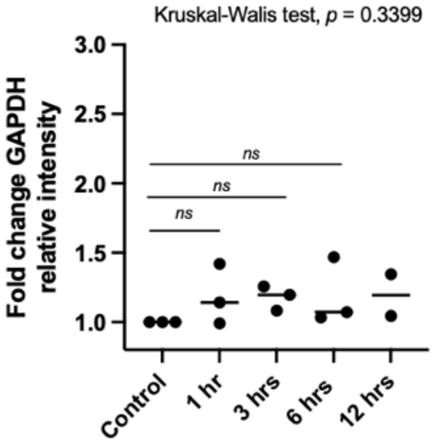

Supplementary Fig. 15 | TNF-α does not regulate GAPDH expression. Quantification of GAPDH band intensities from three independent experiments. HPCs were treated with 1 ng/ml TNF-α for 1, 3, 6, or 12 h. Data represent mean ± SEM of n = 3 independent experiments (control, 1, 3, and 6 h) and n = 2 independent experiments for the 12 h timepoint. Statistical analysis: Kruskal-Wallis test followed by Dunn's post hoc correction**.** Source data are provided as a Source Data file. HPCs, human hippocampal progenitor cells; TNF-α, tumour necrosis factor alpha; GAPDH, glyceraldehyde 3-phosphate dehydrogenase; SEM, standard error of the mean.

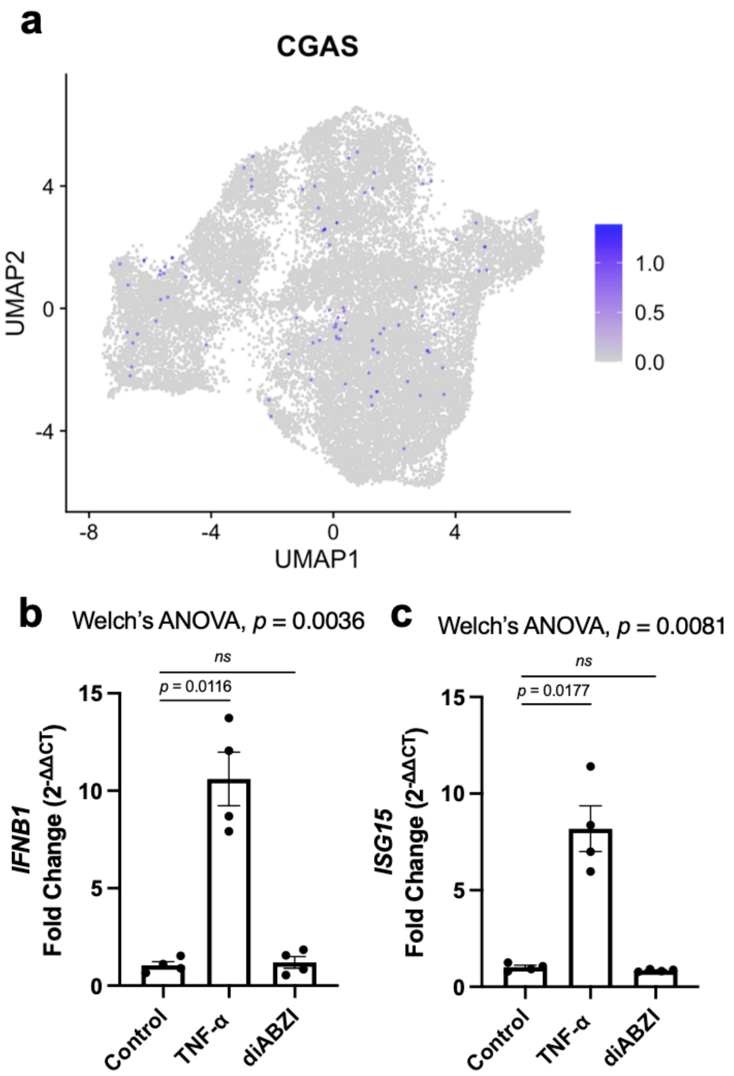

Supplementary Fig. 16 | STING agonist diABZI does not induce *IFNB1* or *ISG15* expression in HPCs. a UMAP plot showing the expression of *CGAS.* scRNA-seq was performed with n = 1 10x library per condition/timepoint. **b** and **c** RT-qPCR analysis of *ISG15* and *IFNB1* expression in HPCs stimulated ± 1 ng/ml TNF-α or ± 100 ng/ml diABZI for 3 hrs. Data is represented as mean ± SEM of n = 4 independent experiments. Statistical analysis: Welch's ANOVA with Games-Howell's multiple comparisons test. Source data are provided as a Source Data file. cGAS, cyclic GMP-AMP synthase; HPCs, human hippocampal progenitor cells; TNF-α, tumour necrosis factor alpha; RT–qPCR, reverse transcription quantitative PCR; UMAP, uniform manifold approximation and projection; SEM, standard error of the mean.

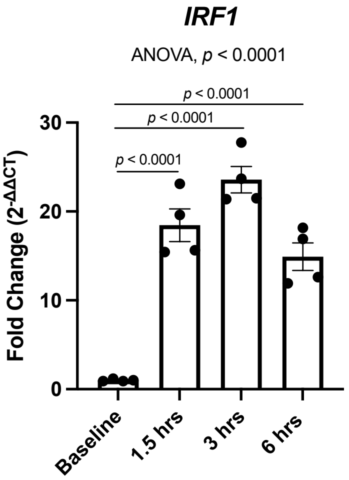

Supplementary Fig. 17 | HPCs upregulate transcription factor *IRF1* in response to TNF-α. RT-qPCR data showing relative *IRF1* gene expression in HPCs treated ± 1 ng/ml TNF-α for 1.5, 3, or 6 hours. Data is presented as mean ± SEM from $n = 4$ independent experiments. Statistical analysis: one-way ANOVA followed by Bonferroni's multiple comparisons test. Source data are provided as a Source Data file. IRF1, interferon regulatory factor 1; HPCs, human hippocampal progenitor cells; TNF-α, tumour necrosis factor alpha; RT–qPCR, reverse transcription quantitative PCR; SEM, standard error of the mean.

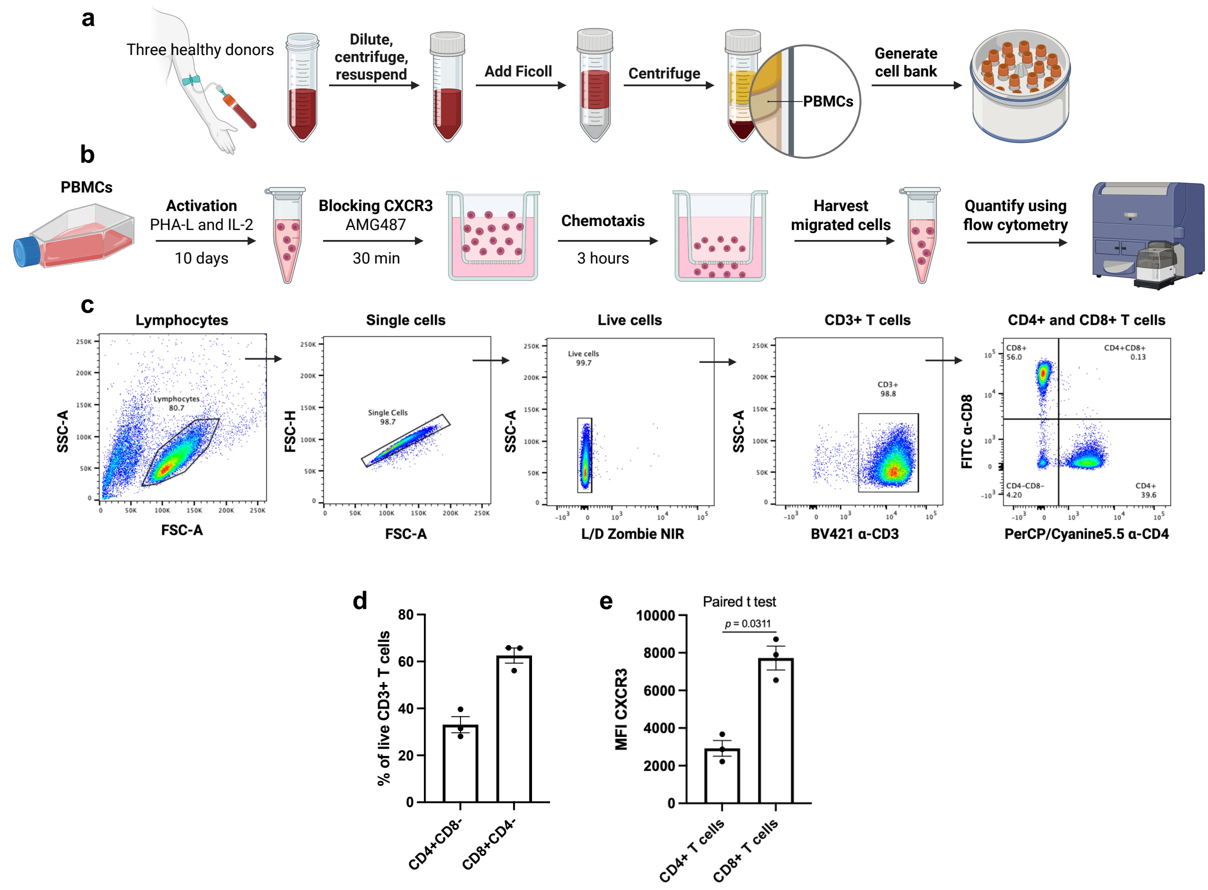

Supplementary Fig. 18 | Transwell chemotaxis assay using primary human T cells to study CXCR3-dependent migration. a, PBMCs were isolated from leukocyte cones from three healthy donors using a Ficoll-Paque density gradient, and a cell bank was generated for each donor. Created in BioRender. Nissen, T. (2026) https://BioRender.com/mwyyikk. **b,** Schematic overview of the T cell chemotaxis assay. Created in BioRender. Nissen, T. (2026) https://BioRender.com/ru0pis2. **c,** Representative gating strategy to identify CD4+ and CD8+ T cells. **d,** Percentage of T cell subsets following the activation protocol. Data is presented as mean ± SEM of n = 3 healthy donors. **e,** Quantification of CXCR3 MFI (median) between CD4+ T cells and CD8+ T cells. Data is presented as mean ± SEM of n = 3 healthy donors. Statistical analysis: paired, two-tailed, parametric t-test. Source data are provided as a Source Data file. PBMCs, peripheral blood mononuclear cells; CXCR3, C-X-C motif chemokine receptor 3; MFI, mean fluorescence intensity; SEM, standard error of the mean.

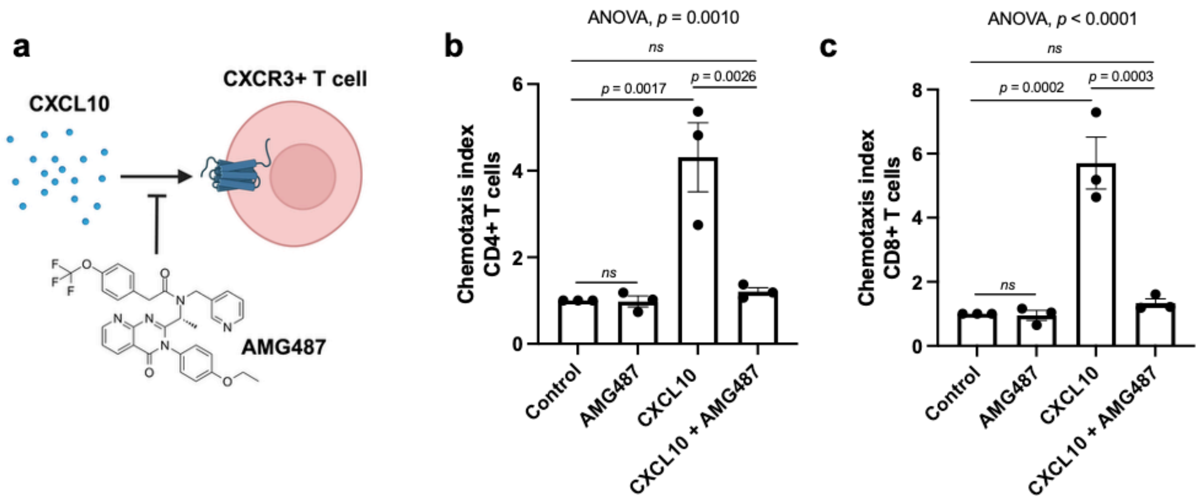

Supplementary Fig. 19 | Recombinant CXCL10 promotes CXCR3-dependent T cell recruitment. a, CXCL10 drives T cell chemotaxis by activating CXCR3. AMG487 is a selective CXCR3 antagonist. Created in BioRender. Nissen, T. (2026) https://BioRender.com/vc9pd3t. **b**, and **c**, Quantification of the chemotactic response (chemotaxis index) of **b** CD4+ T cells and **c** CD8+ T cells treated ± 1 μM AMG487 or DMSO in response to control media or media containing 100 ng/ml recombinant hCXCL10. The chemotactic index is the ratio of the number of cells migrated in response to a stimulus, as compared with controls. Data is represented as mean ± SEM of *n* = 3 healthy donors. Statistical analysis: one-way ANOVA followed by Bonferroni's multiple comparisons test. Source data are provided as a Source Data file. CXCL10, C-X-C motif chemokine ligand 10; CXCR3, C-X-C motif chemokine receptor 3; SEM, standard error of the mean.

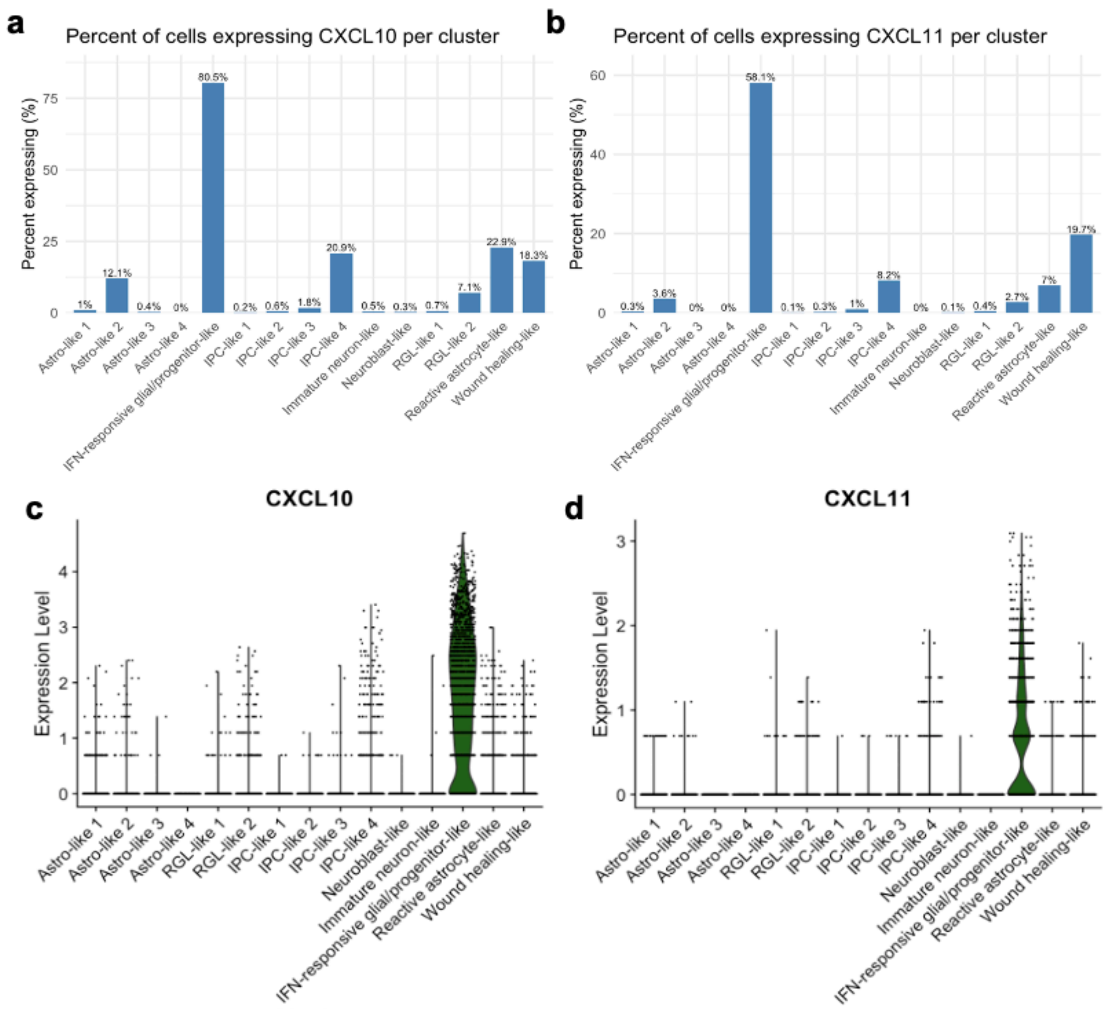

Supplementary Fig. 20 | Cellular source of *CXCL10* and *CXCL11*. a and **b** Bar plots showing the percentage of cells per cluster expressing *CXCL10* and *CXCL11*, respectively. Source data are provided as a Source Data file. **c** and **d** Violin plots displaying the expression levels of *CXCL10* and *CXCL11*, respectively. scRNA-seq was performed with *n* = 1 10x library per condition/timepoint. CXCL10, C-X-C motif chemokine ligand 10; CXCL11, C-X-C motif chemokine ligand 11.

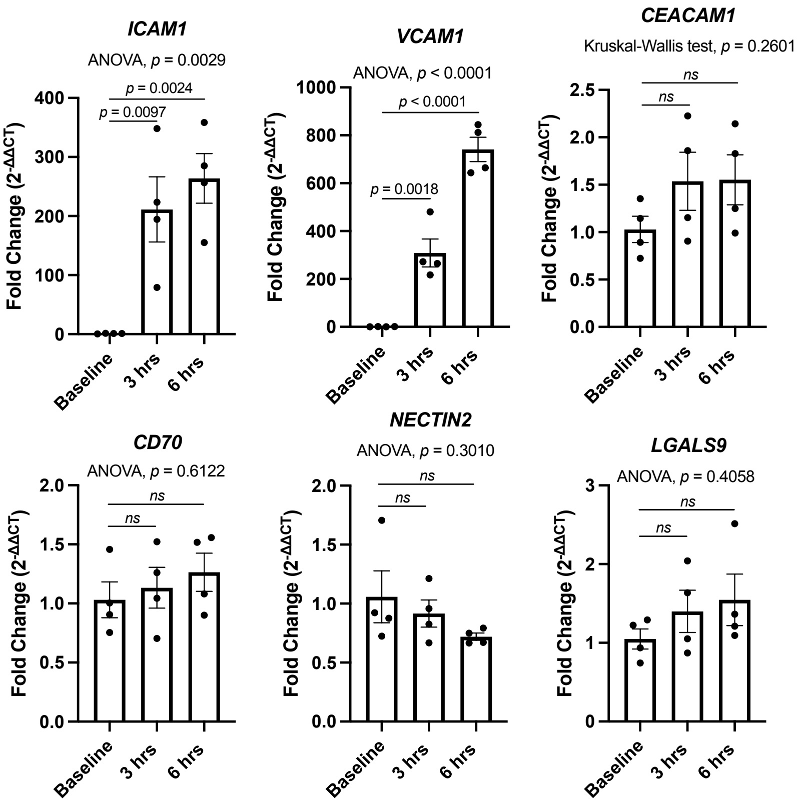

Supplementary Fig. 21 | HPCs upregulate molecules involved in T cell crosstalk in response to TNF-α. RT-qPCR data showing relative gene expression of *ICAM1, VCAM1, CEACAM1, CD70, NECTIN2, and LGALS9* in HPCs treated ± 1 ng/ml TNF-α for 1.5, 3, or 6 hours. Data is presented as mean ± SEM of *n* = 4 independent experiments. Statistical analysis: one-way ANOVA followed by Bonferroni's multiple comparisons test or Kruskal-Wallis test followed by Dunn's post hoc correction as indicated. Source data are provided as a Source Data file. HPCs, human hippocampal progenitor cells; TNF-α, tumour necrosis factor alpha; RT–qPCR, reverse transcription quantitative PCR; SEM, standard error of the mean; ICAM1, intercellular adhesion molecule 1; VCAM1, vascular cell adhesion molecule 1; CEACAM1, carcinoembryonic antigen-related cell adhesion molecule 1; CD70, CD70 molecule; NECTIN2, nectin cell adhesion molecule 2; LGALS9, galectin 9.

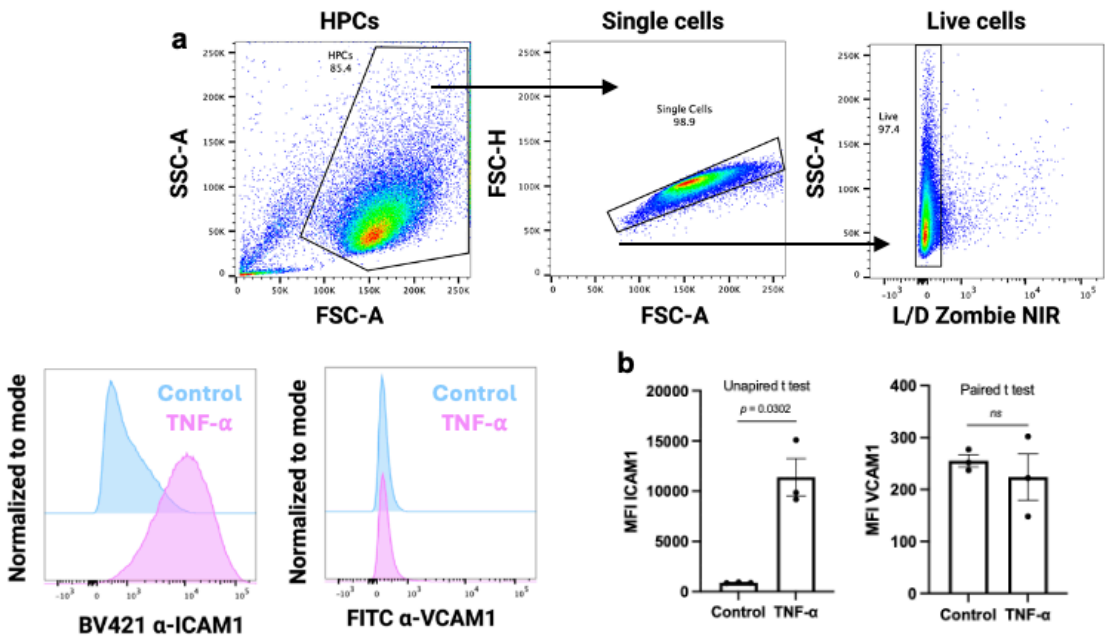

Supplementary Fig. 22 | Cell surface expression of ICAM1 and VCAM1 on HPCs in response to TNF-α. a, Gating strategy for assessing cell surface protein expression on live, single HPCs. Cells were first gated by forward scatter (FSC) and side scatter (SSC) to exclude debris and identify the main population. Singlets were selected using FSC-A versus FSC-H, and live cells were identified by negative selection using the Zombie NIR viability dye. Representative histograms show ICAM1 and VCAM1 on the control-treated (blue) and TNF-α-treated (pink) HPCs. **b**, Quantification of ICAM1 and VCAM1 surface expression by mean fluorescence intensity (MFI) on HPCs treated ± 1 ng/ml TNF-α for 24 h, measured by flow cytometry. Data is presented as mean ± SEM of n = 3 independent experiments. Statistical analysis for ICAM1: two-tailed, unpaired t test with Welch's correction. Statistical analysis for VCAM1: two-tailed, unpaired t test. Source data are provided as a Source Data file. HPCs, human hippocampal progenitor cells; TNF-α, tumour necrosis factor alpha; FSC, forward scatter; SSC, side scatter; MFI, mean fluorescence intensity; SEM, standard error of the mean; ICAM1, intercellular adhesion molecule 1; VCAM1, vascular cell adhesion molecule 1.

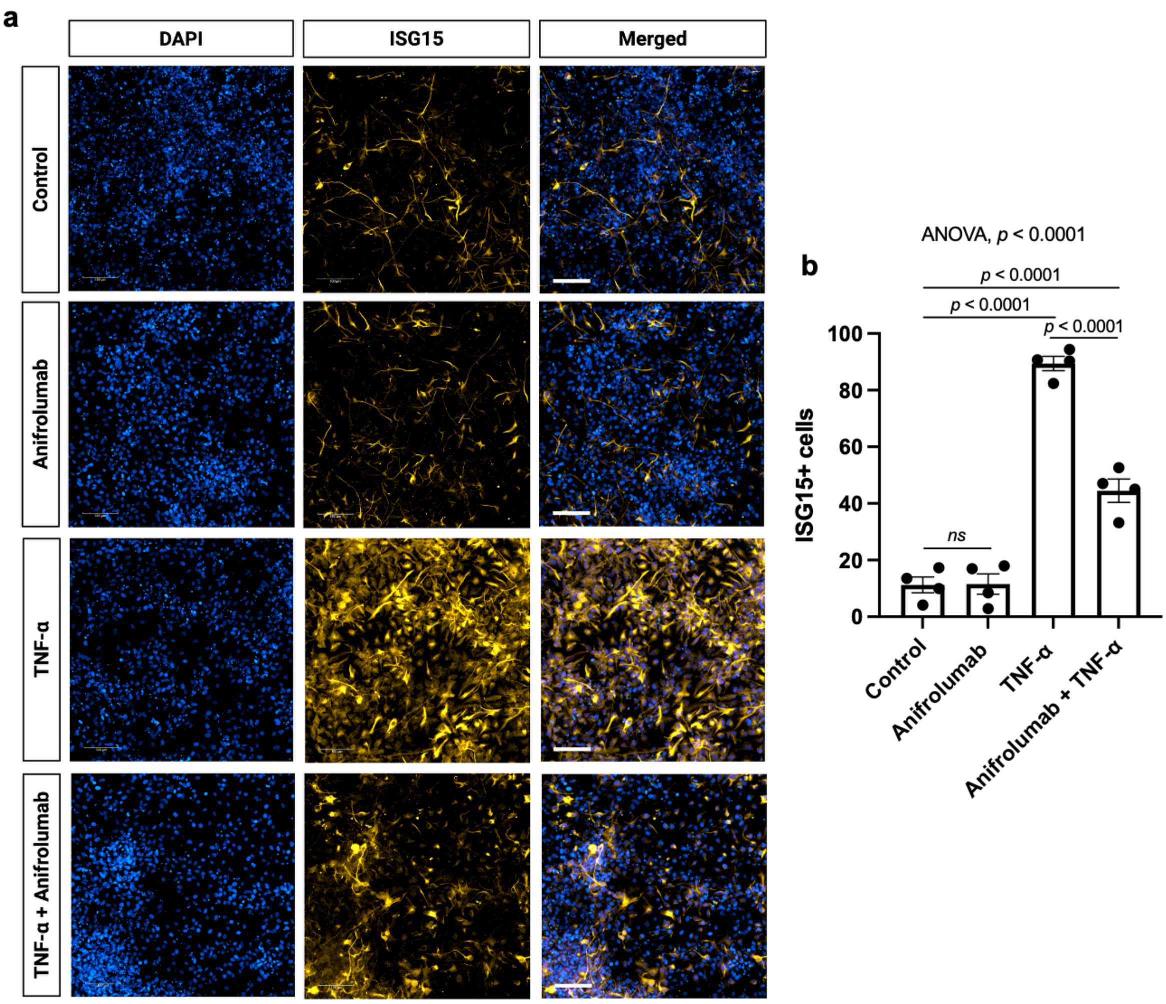

Supplementary Fig. 23 | Blocking IFNAR decreases the upregulation of ISG15+ differentiating HPCs in response to chronic TNF-α. a, Representative images of n = 4 independent experiments with similar results showing showing the expression of ISG15 (orange) in HPCs differentiated for seven days treated chronically with ± 1 ng/ml TNF-α ± 10 µg/ml anifrolumab. Scale bar, 100 µm. **b,** Quantification of the percentage of ISG15+ cells based on panel **a**. Data represent mean ± SEM of n = 4 independent experiments. Statistical analysis: one-way ANOVA followed by Bonferroni's multiple comparisons test. Source data are provided as a Source Data file. HPCs, human hippocampal progenitor cells; TNF-α, tumour necrosis factor alpha; IFNAR, interferon alpha receptor; ISG15, interferon-stimulated gene 15; SEM, standard error of the mean.

**Target**	**Host**	**Clone**	**Manufacturer**	**Catalogue number**	**Dilution**
Nestin	Mouse	10C2	Merck Millipore (Chemicon)	MAB5326	1:1000
MAP2	Mouse	HM-2	Abcam	ab11267	1:500
DCX	Rabbit	Polyclonal	Abcam	ab18723	1:500

STAT1	Mouse	9H2	Cell Signaling Technology	9176	1:1000
ISG15	Rabbit	Polyclonal	Proteintech	15981-1-AP	1:500
NF-κB p65	Mouse	F-6	Santa Cruz Biotechnology	sc-8008	1:500
Cleaved caspase-3 (Asp175)	Rabbit	5A1E	Cell Signaling Technology	9664	1:500
TNFR1	Rabbit	Polyclonal	Proteintech	21574-1-AP	1:400
TNFR2	Rabbit	Polyclonal	Proteintech	19272-1-AP	1:400
Anti-rabbit Alexa Fluor 555	Donkey	Polyclonal	Invitrogen	A-31571	1:500
Anti-rabbit Alexa Fluor 488	Donkey	Polyclonal	Invitrogen	A-21202	1:500

Supplementary Table 1 | Primary and secondary antibodies used for immunocytochemistry

Step	Temperature (°C)	Time (minutes)
1	25	10
2	37	120
3	85	5
4	4	∞

Supplementary Table 2 | Thermal cycler program employed for cDNA synthesis

Step	Temperature (°C)	Time
1	50	2 minutes
2	95	10 minutes
3 (40 cycles)	95	15 seconds
4 (40 cycles)	60	1 minute
5	95	15 seconds

Supplementary Table 3 | Thermal cycling conditions employed for RT-qPCR

Target	Host	Clone	Manufacturer	Catalogue number	Dilution
p-STAT1 (Tyr701)	Rabbit	D4A7	Cell Signaling Technology	7649	1:1000
p-STAT2 (Tyr690)	Rabbit	D3P2P	Cell Signaling Technology	88410	1:1000
GAPDH	Mouse	1E6D9	Proteintech	60004-1-Ig	1:10000

STAT1	Mouse	9H2	Cell Signaling Technology	9176	1:1000
STAT2	Rabbit	D9J7L	Cell Signaling Technology	72604	1:1000
Anti-rabbit IgG, HRP	Donkey	Polyclonal	GE Healthcare (Amersham/Cytiva)	NA9340V	1:3000
Anti-mouse IgG, HRP	Goat	Polyclonal	Santa Cruz Biotechnology	D1321	1:9000

Supplementary Table 4 | Antibodies used for western blot experiments

Chemokine	Full name	Alias
CCL2	Chemokine (CC motif) ligand 2	MCP-1
CCL3	Chemokine (CC motif) ligand 3	MIP-1α
CCL4	Chemokine (CC motif) ligand 4	MIP-1β
CXCL1	Chemokine (CXC motif) ligand 1	GROα
CXCL5	Chemokine (CXC motif) ligand 5	ENA-78
CXCL8	Chemokine (CXC motif) ligand 8	IL-8
CXCL9	Chemokine (CXC motif) ligand 9	MIG
CXCL10	Chemokine (CXC motif) ligand 10	IP-10
CXCL11	Chemokine (CXC motif) ligand 11	I-TAC
CCL11	Chemokine (CC motif) ligand 11	Eotaxin
CCL17	Chemokine (CC motif) ligand 17	TARC
CCL20	Chemokine (CC motif) ligand 20	MIP-3α

Supplementary Table 5 | Chemokines included in the human pro-inflammatory chemokine panel

Target	Host	Clone	Fluorophore	Manufacturer	Catalogue number	Dilution
ICAM1	Mouse	HA58	BV421	BioLegend	353132	1:100
VCAM1	Mouse	BBIG-V3 (IE10)	FITC	R&D Systems	BBA22	1:100
Tetherin (BST2)	Mouse	RS38E	APC	BioLegend	348410	1:100

Supplementary Table 6 | Antibodies used to analyse cell surface protein expression on HPCs

Target	Host	Clone	Fluorophore	Manufacturer	Catalogue number	Dilution
CXCR3	Mouse	G025H7	PE	BioLegend	353706	1:100
CD3	Mouse	OKT3	BV421	BioLegend	317344	1:100

CD4	Mouse	OKT4	PerCP/Cy5.5	BioLegend	317428	1:100
CD8	Mouse	SK1	FITC	BioLegend	980908	1:100

Supplementary Table 7 | Antibodies used to analyse cell surface protein expression on immune cells
