# Supplementary material for: TNF-α induces type I IFN signalling to suppress neurogenesis and recruit T cells
Source: Nat Commun. 2026 Jul 7;17:5287. doi: 10.1038/s41467-026-74104-x (PMC13342090; doi:10.1038/s41467-026-74104-x)
Supplement: Supplementary file 2 — Description of Additional Supplementary Files [file 41467_2026_74104_MOESM2_ESM.pdf]

Description of Additional Supplementary Files

File name: Supplementary Data 1
Description: Excel spreadsheet listing all nucleotide primers used in the study.
