# Supplementary material for: TNF-α induces type I IFN signalling to suppress neurogenesis and recruit T cells
Source: Nat Commun. 2026 Jul 7;17:5287. doi: 10.1038/s41467-026-74104-x (PMC13342090; doi:10.1038/s41467-026-74104-x)
Supplement: Supplementary file 4 — Reporting Summary [file 41467_2026_74104_MOESM4_ESM.pdf]

# nature portfolio

Corresponding author(s): Sandrine Thuret and Linda Klavinskis

Last updated by author(s): 19/4/2026



# Reporting Summary

Nature Portfolio wishes to improve the reproducibility of the work that we publish. This form provides structure for consistency and transparency in reporting. For further information on Nature Portfolio policies, see our Editorial Policies and the Editorial Policy Checklist.

## Statistics

For all statistical analyses, confirm that the following items are present in the figure legend, table legend, main text, or Methods section.

| n/a | Confirmed | |
|---|---|---|
| ☐ | ☒ | The exact sample size (*n*) for each experimental group/condition, given as a discrete number and unit of measurement |
| ☐ | ☒ | A statement on whether measurements were taken from distinct samples or whether the same sample was measured repeatedly |
| ☐ | ☒ | The statistical test(s) used AND whether they are one- or two-sided<br>*Only common tests should be described solely by name; describe more complex techniques in the Methods section.* |
| ☒ | ☐ | A description of all covariates tested |
| ☐ | ☒ | A description of any assumptions or corrections, such as tests of normality and adjustment for multiple comparisons |
| ☐ | ☒ | A full description of the statistical parameters including central tendency (e.g. means) or other basic estimates (e.g. regression coefficient) AND variation (e.g. standard deviation) or associated estimates of uncertainty (e.g. confidence intervals) |
| ☐ | ☒ | For null hypothesis testing, the test statistic (e.g. *F*, *t*, *r*) with confidence intervals, effect sizes, degrees of freedom and *P* value noted<br>*Give P values as exact values whenever suitable.* |
| ☒ | ☐ | For Bayesian analysis, information on the choice of priors and Markov chain Monte Carlo settings |
| ☒ | ☐ | For hierarchical and complex designs, identification of the appropriate level for tests and full reporting of outcomes |
| ☒ | ☐ | Estimates of effect sizes (e.g. Cohen's *d*, Pearson's *r*), indicating how they were calculated |

*Our web collection on statistics for biologists contains articles on many of the points above.*

## Software and code

Policy information about availability of computer code

**Data collection**

• High-content imaging was performed using the Opera Phenix Plus High-Content Screening System (PerkinElmer).
• RNA concentrations were measured using a DS-11FX+ (DeNovix), and reverse transcription was performed using the Biometra Trio (Analytik Jena).
• RT-qPCR was performed on a QuantStudio 5 Real-Time PCR System (Applied Biosystems).
• Membranes were imaged using an ImageQuant 800 Imager (Amersham).
• Flow cytometry data were acquired using a FACSCanto II flow cytometer (BD Biosciences) with FACSDiva (v9.2), including high-throughput sampler acquisition with the following settings: flow rate 3.0, sample volume 50 µl, mixing volume 50 µl, mixing speed 200, and two washes with 200 µl wash volume
• For single-cell RNA sequencing, 18,000 cells per pool were loaded onto a Chromium X instrument (10x Genomics) for droplet-based capture.
• cDNA libraries were prepared using the Chromium Single Cell 3′ Reagent Kit v3 (10x Genomics, Cat. No. PN 1000268).
• Libraries were sequenced on an Illumina NextSeq 2000 system using 50 bp paired-end reads.

**Data analysis**

• High-content imaging data were analysed using Harmony (v4.9) (PerkinElmer).
• RT-qPCR data were analysed using QuantStudio Design & Analysis Software (v1.5.1, Applied Biosystems).
• Western blot band intensities were quantified using Image Studio (v6.0.28, LI-COR BioSciences).
• Flow cytometry data were analysed using FlowJo (v10.10) (BD Biosciences).
• Cytokine bead array data were analysed using the LEGENDplex data analysis software suite from BioLegend (v8.0).
• FASTQ files from gene expression and HTO libraries were processed using Cell Ranger (v7.1) (10x Genomics) with the cellranger multi

pipeline.
• Data were imported into R (v4.3.2) and analysed using Seurat (v5.3.0).
• Statistical analyses were performed using GraphPad Prism (v10.4.1) and R (v4.3.2) with the rstatix package (v0.7.2).
• Gene Ontology over-representation analysis was performed using clusterProfiler (v4.14.6).
• Transcription factor activity was inferred using decoupleR (v2.8).
• Regulatory network data (CollecTRI) were retrieved using OmnipathR (v3.14.0)
• The full scRNA analysis pipeline is available on Github: https://github.com/thuretlabkcl/TNF-scRNAseq-neurogenesis

For manuscripts utilizing custom algorithms or software that are central to the research but not yet described in published literature, software must be made available to editors and reviewers. We strongly encourage code deposition in a community repository (e.g. GitHub). See the Nature Portfolio guidelines for submitting code & software for further information.

## Data

Policy information about availability of data

All manuscripts must include a data availability statement. This statement should provide the following information, where applicable:

- Accession codes, unique identifiers, or web links for publicly available datasets
- A description of any restrictions on data availability
- For clinical datasets or third party data, please ensure that the statement adheres to our policy

Source data are provided with this paper. Raw sequencing data have been deposited in the NCBI Sequence Read Archive under BioProject accession PRJNA1397568.

## Research involving human participants, their data, or biological material

Policy information about studies with human participants or human data. See also policy information about sex, gender (identity/presentation), and sexual orientation and race, ethnicity and racism.

| | |
|---|---|
| Reporting on sex and gender | Experiments using the human HPC0A07/03A hippocampal progenitor cell line were performed in a female-derived line (the only available version of this cell line). As a result, sex and gender were not considered as variables in the study design for these experiments and sex- or gender-stratified analyses were not performed. Our findings using this cell line only applies to the female. PBMCs were obtained from three healthy human donors; donor sex and gender identity information were not available/not collected for these de-identified samples. Therefore, data could not be disaggregated by sex or gender. |
| Reporting on race, ethnicity, or other socially relevant groupings | NA |
| Population characteristics | NA |
| Recruitment | NA |
| Ethics oversight | NA |

Note that full information on the approval of the study protocol must also be provided in the manuscript.

# Field-specific reporting

Please select the one below that is the best fit for your research. If you are not sure, read the appropriate sections before making your selection.

☒ Life sciences ☐ Behavioural & social sciences ☐ Ecological, evolutionary & environmental sciences

For a reference copy of the document with all sections, see nature.com/documents/nr-reporting-summary-flat.pdf

# Life sciences study design

All studies must disclose on these points even when the disclosure is negative.

| | |
|---|---|
| Sample size | Sample sizes were not determined by formal prospective power calculations. For cell-based assays with HPCs, n refers to independent experiments performed on distinct culture preparations/differentiations (or biological replicates as defined in the figure legends). For PBMC-based experiments, n refers to independent human donors. These sample sizes are consistent with those widely used in comparable published studies of human primary cells and cell line-based systems, in which effect sizes of interest are typically large enough to be detected with the replicate numbers used. The specific sample size for each experiment is stated in the corresponding figure legend and Source Data. |
| Data exclusions | For cell culture experiments, samples were excluded only in cases of technical failure (e.g., culture contamination). For scRNA-seq, individual cells were excluded during preprocessing if they failed predefined quality-control thresholds (e.g., high mitochondrial transcript fraction and/or low feature/UMI counts). |
| Replication | All attempts at replication were successful. A minimum of three independent experiments were performed, except for the exploratory scRNA-seq experiment, which was performed with n = 1 10x library per condition/time point. However, key findings from the scRNA-seq experiment were validated in subsequent experiments with at least three independent biological replicates. |
| Randomization | Randomization was not applied because samples were defined by experimental condition; all conditions were handled in parallel |

| | |
|---|---|
| Blinding | Investigators were not blinded to experimental group allocation during data collection and analysis because treatments were applied as part of the experimental design |

# Reporting for specific materials, systems and methods

We require information from authors about some types of materials, experimental systems and methods used in many studies. Here, indicate whether each material, system or method listed is relevant to your study. If you are not sure if a list item applies to your research, read the appropriate section before selecting a response.

## Materials & experimental systems

| n/a | Involved in the study |
|---|---|
| ☐ | ☒ Antibodies |
| ☐ | ☒ Eukaryotic cell lines |
| ☒ | ☐ Palaeontology and archaeology |
| ☒ | ☐ Animals and other organisms |
| ☒ | ☐ Clinical data |
| ☒ | ☐ Dual use research of concern |
| ☒ | ☐ Plants |

## Methods

| n/a | Involved in the study |
|---|---|
| ☒ | ☐ ChIP-seq |
| ☐ | ☒ Flow cytometry |
| ☒ | ☐ MRI-based neuroimaging |

## Antibodies

Antibodies used

Primary and secondary antibodies used for immunocytochemistry:
Target Host Clone Manufacturer Catalogue number Dilution
Nestin Mouse 10C2 Merck Millipore (Chemicon) MAB5326 1:1000
MAP2 Mouse HM-2 Abcam ab11267 1:500
DCX Rabbit Polyclonal Abcam ab18723 1:500
STAT1 Mouse 9H2 Cell Signaling Technology 9176 1:1000
ISG15 Rabbit Polyclonal Proteintech 15981-1-AP 1:500
NF-κB p65 Mouse F-6 Santa Cruz Biotechnology sc-8008 1:500
Cleaved caspase-3 (Asp175) Rabbit 5A1E Cell Signaling Technology 9664 1:500
TNFR1 Rabbit Polyclonal Proteintech 21574-1-AP 1:400
TNFR2 Rabbit Polyclonal Proteintech 19272-1-AP 1:400
Anti-rabbit Alexa Fluor 555 Donkey Polyclonal Invitrogen A-31571 1:500
Anti-rabbit Alexa Fluor 488 Donkey Polyclonal Invitrogen A-21202 1:500

Antibodies used for western blot experiments:
Target Host Clone Manufacturer Catalogue number Dilution
p-STAT1 (Tyr701) Rabbit D4A7 Cell Signaling Technology 7649 1:1000
p-STAT2
(Tyr690) Rabbit D3P2P Cell Signaling Technology 88410 1:1000
GAPDH Mouse 1E6D9 Proteintech 60004-1-Ig 1:10000
STAT1 Mouse 9H2 Cell Signaling Technology 9176 1:1000
STAT2 Rabbit D9J7L Cell Signaling Technology 72604 1:1000
Anti-rabbit IgG, HRP Donkey Polyclonal GE Healthcare (Amersham/Cytiva) NA9340V 1:3000
Anti-mouse IgG, HRP Goat Polyclonal Santa Cruz Biotechnology D1321 1:9000

Antibodies used to analyse cell surface protein expression on HPCs:
Target Host Clone Fluorophore Manufacturer Catalogue number Dilution
ICAM1 Mouse HA58 BV421 BioLegend 353132 1:100
VCAM1 Mouse BBIG-V3 (IE10) FITC R&D Systems BBA22 1:100
Tetherin (BST2) Mouse RS38E APC BioLegend 348410 1:100

Antibodies used to analyse cell surface protein expression on immune cells:
Target Host Clone Fluorophore Manufacturer Catalogue number Dilution
CXCR3 Mouse G025H7 PE BioLegend 353706 1:100
CD3 Mouse OKT3 BV421 BioLegend 317344 1:100
CD4 Mouse OKT4 PerCP/Cy5.5 BioLegend 317428 1:100
CD8 Mouse SK1 FITC BioLegend 980908 1:100

Lot numbers were not recorded for the antibodies used in this study.

| | |
|---|---|
| Validation | Primary antibodies were selected based on manufacturer validation data (species reactivity and application validation stated on supplier datasheets and/or antibody registry profiles) and prior published use. All antibodies were used according to the suppliers' recommended applications and dilutions for human targets where applicable.<br><br>Immunocytochemistry (ICC): Specificity was assessed by omission controls (secondary-only/no primary) and by verifying expected cellular/subcellular localization and cell-type–appropriate staining patterns (e.g., Nestin in progenitors; MAP2/DCX in neuronal lineage; cleaved caspase-3 in apoptotic cells). Where relevant, pathway activation produced expected localization changes (e.g., NF-κB p65 nuclear translocation) and/or inducible expression patterns (e.g., ISG15).<br><br>Western blot: Specificity was assessed by the presence of a predominant band at the expected molecular weight and, for phospho-specific antibodies, stimulus-dependent signal consistent with phosphorylation. Loading was controlled using GAPDH.<br>Flow cytometry: Directly conjugated antibodies were used as specified by the manufacturers. Staining was validated using standard flow controls (single-stain compensation controls and fluorescence-minus-one and/or isotype controls as appropriate), and by confirming expected expression patterns using established gating strategies (e.g., CD3/CD4/CD8 subsets).<br><br>Immunocytochemistry and immunofluorescence antibodies<br>The anti-Nestin antibody (clone 10C2, Merck Millipore, MAB5326) has been validated by the manufacturer for ICC, IHC, and western blotting, with expected staining of neural progenitor cells as a positive control. The anti-MAP2 antibody (clone HM-2, Abcam, ab11267) has been validated by the manufacturer for ICC and western blotting, detecting a band at the expected molecular weight of 200 kDa; in this study, the antibody produced the characteristic neuronal somatodendritic staining pattern consistent with the known subcellular localisation of MAP2, and the clone has been used in over 200 peer-reviewed publications. The anti-DCX antibody (polyclonal, Abcam, ab18723) has been validated by the manufacturer for ICC/IF in human samples and has been used in over 600 peer-reviewed publications, including studies demonstrating staining of neuroblasts in the dentate gyrus and hIPSC-derived neurons. The anti-NF-κB p65 antibody (clone F-6, Santa Cruz Biotechnology, sc-8008) has been validated by the manufacturer by western blotting, detecting the correct size band, and has been independently knockout-validated in the peer-reviewed literature (Labome VAD; PMID: 27270613). The antibody is cited in over 2,000 publications. The anti-cleaved caspase-3 antibody (clone 5A1E, Cell Signaling Technology, 9664) has been validated by the manufacturer by western blotting with a correct size band in Jurkat cells and by ICC demonstrating cytoplasmic staining in HT-29 cells following treatment with the apoptosis-inducing agent staurosporine; the antibody is cited in over 7,000 peer-reviewed publications. The anti-TNFR1 (polyclonal, Proteintech, 21574-1-AP) and anti-TNFR2 (polyclonal, Proteintech, 19272-1-AP) antibodies have both been knockout/knockdown validated by the manufacturer and detect bands at the expected molecular weights by western blotting.<br><br>Western blotting antibodies<br>The anti-p-STAT1 (Tyr701, clone D4A7, Cell Signaling Technology, 7649) and anti-p-STAT2 (Tyr690, clone D3P2P, Cell Signaling Technology, 88410) antibodies have been validated by the manufacturer by western blotting in multiple cell lines, with correct size bands appearing specifically in response to human interferon-α treatment, confirming phosphorylation-state specificity. The anti-STAT1 antibody (clone 9H2, Cell Signaling Technology, 9176) has been knockout-validated by the manufacturer. The anti-STAT2 antibody (clone D9J7L, Cell Signaling Technology, 72604) has been validated by western blotting with the expected size band and by ICC demonstrating nuclear translocation following IFN-α stimulation consistent with known STAT2 biology. The anti-ISG15 antibody (polyclonal, Proteintech, 15981-1-AP) has been knockout/knockdown validated by the manufacturer. The anti-GAPDH antibody (clone 1E6D9, Proteintech, 60004-1-Ig) has been knockout/knockdown validated by the manufacturer and is cited in over 14,000 peer-reviewed publications.<br>Flow cytometry antibodies<br>The anti-ICAM1 antibody(clone HA58, BioLegend, 353132) has been validated by the manufacturer for flow cytometry and has been independently knockout-validated in the peer-reviewed literature using CRISPR/Cas9-mediated ICAM-1 deletion confirmed by flow cytometry with this clone (Rajani et al., Nature Communications, 2025). The anti-VCAM1 antibody (clone BBIG-V3/IE10, R&D Systems, BBA22) has been validated by the manufacturer for flow cytometry in human samples; specificity is further supported by consistent staining patterns across multiple independent publications and the use of matched isotype controls. The anti-BST2/Tetherin antibody (clone RS38E, BioLegend, 348410) specificity is supported by the clone's widespread use in peer-reviewed flow cytometry studies of BST2 biology, and the well-characterised biology of BST2 as an interferon-stimulated gene in line with its upregulation in this study. The anti-CXCR3 antibody (clone G025H7, BioLegend, 353706) specificity is supported by the manufacturer-provided functional validation demonstrating that G025H7 neutralises CXCL10/11-induced chemotaxis of BaF3 cells expressing human CXCR3, confirming specific engagement of the target receptor. The CD3 (clone OKT3, BioLegend, 317344), CD4 (clone OKT4, BioLegend, 317428), and CD8α (clone SK1, BioLegend, 980908) antibodies are among the most extensively characterised antibodies in immunology, each with decades of validated use. |

# Eukaryotic cell lines

Policy information about cell lines and Sex and Gender in Research

| | |
|---|---|
| Cell line source(s) | The human, female-derive hippocampal progenitor cell line (HPC0A07/03A) was sourced from ReNeuron Ltd., Surrey, U.K.Human PBMCs were sourced from the NHS Blood and Transplant (leukocyte cones). |
| Authentication | Karyotyping and multiple gene expression analyses across parallel studies in the labs using this cell line. |
| Mycoplasma contamination | The cell line is routinely tested for mycoplasma and we can confirm it has never been tested positive |
| Commonly misidentified lines<br>(See ICLAC register) | NA |

# Plants

Seed stocks

*Report on the source of all seed stocks or other plant material used. If applicable, state the seed stock centre and catalogue number. If plant specimens were collected from the field, describe the collection location, date and sampling procedures.*

Novel plant genotypes

*Describe the methods by which all novel plant genotypes were produced. This includes those generated by transgenic approaches, gene editing, chemical/radiation-based mutagenesis and hybridization. For transgenic lines, describe the transformation method, the number of independent lines analyzed and the generation upon which experiments were performed. For gene-edited lines, describe the editor used, the endogenous sequence targeted for editing, the targeting guide RNA sequence (if applicable) and how the editor was applied.*

Authentication

*Describe any authentication procedures for each seed stock used or novel genotype generated. Describe any experiments used to assess the effect of a mutation and, where applicable, how potential secondary effects (e.g. second site T-DNA insertions, mosiacism, off-target gene editing) were examined.*

# Flow Cytometry

## Plots

Confirm that:

- [x] The axis labels state the marker and fluorochrome used (e.g. CD4-FITC).
- [x] The axis scales are clearly visible. Include numbers along axes only for bottom left plot of group (a 'group' is an analysis of identical markers).
- [x] All plots are contour plots with outliers or pseudocolor plots.
- [x] A numerical value for number of cells or percentage (with statistics) is provided.

## Methodology

| | |
|---|---|
| Sample preparation | As desbribed in the respectie methods sections |
| Instrument | FACSCanto II |
| Software | BD FACSDiva, FlowJo (10.10.0) , and Prism (Version 10.6.1) |
| Cell population abundance | NA |
| Gating strategy | As described in the manuscript |

- [x] Tick this box to confirm that a figure exemplifying the gating strategy is provided in the Supplementary Information.
