# Supplementary material for: TNF-α induces type I IFN signalling to suppress neurogenesis and recruit T cells
Source: Nat Commun. 2026 Jul 7;17:5287. doi: 10.1038/s41467-026-74104-x (PMC13342090; doi:10.1038/s41467-026-74104-x)
Supplement: Supplementary file 5 — Transparent Peer Review file [file 41467_2026_74104_MOESM5_ESM.pdf]

nature portfolio

Peer Review File

TNF-α induces type I IFN signalling to suppress neurogenesis and recruit T cells

Corresponding Author: Professor Sandrine Thuret

This file contains all reviewer reports in order by version, followed by all author rebuttals in order by version.

Version 0:

Reviewer comments:

Reviewer #1

(Remarks to the Author)
Nissen et al report on studies evaluating the effect of TNF on human hippocampal progenitor cells (HPCs). They used two doses of the cytokine, and found that chronic exposure, especially at high dose, led to loss of neuroblasts and neurons, which is validated via immunocytochemistry. They present scRNAseq data derived from cell clusters of HPCs treated with TNF at different time-points and doses. The authors highlight changes in IFNAR signaling pathways, including cytokine and chemokine mRNAs and conclude that TNF suppresses neurogenesis and promotes expression of inflammatory molecules. A major problem with this conclusion is that the authors fail to evaluate TNF receptors in their clusters and do not explore the vulnerability of different cells to TNF-mediated cell death. Indeed, much of the high dose TNF data indicates loss of clusters (or cells) without increases in progenitors or alternative lineages. Without examination of this alternative hypothesis, the data cannot support the conclusions of the authors. Finally, the authors perform a number of experiments that are tangential to their original goals and depict known pathways that regulate type I IFN expression and detection of bioactive, IFNAR-inducible CXCL10 in the media of TNF-treated HPCs, the latter of which was not traced to any specific cell type. The authors finally perform an experiment in which they block IFNAR and demonstrate less TNF-mediated decrease in percentages of DCX+ cells. However, the suppression of neurogenesis by type I IFN has previously been published by others (see 10.1016/j.stemcr.2014.05.015).
Below are concerns by section or Figure:
Introduction lacks inclusion of literature demonstrating effects of viruses on adult neurogenesis in humans and mice. See:
1. (2024) Nat Immunol. Jul;25(7):1158-1171.
2. (2022) Brain Aug 25:awac270.
3. (2018) Nat. Immunol., Feb;19(2):151-161
4. (2019) Stem Cells. Nov;37(11):1467-1480
5. (2016) Cell Stem Cell. Nov 3;19(5):593-598.

Figure 1. The decrease in DAPI staining with pyknotic nuclei in panel f suggests dose-dependent cell death in response to TNF. As TNF can induce apoptosis, the authors should evaluate this in their model. Also, there are no data demonstrating TNF receptors. It is important to show which clusters express these receptors, which induce different signaling pathways upon TNF binding with TNFR1 promoting death pathways via caspase activation. Thus, the conclusion that TNF reprograms HPCs is not supported by this data.

Figure 2. From which time-points were the UMAP and panels d-h created? Please include the identities of the 15 clusters in the figure, particularly their color coding in panel c. Panel d Y axis is too jumbled to interpret. What are clusters 13-15? Panel h and Supplemental data support the notion that TNF promotes cell death programs, which should be evaluated.

Figure 3. The differences in innate immune molecules could be due to loss of clusters or differential responses to TNF depending on which receptors are expressed. Again, experiments examining TNFR1-mediated cell death could explain loss of clusters. In addition, type I IFN responses can enhance TNF-mediated apoptosis in cells that express TNFR1. Also, as the authors do not provide the identify of clusters 13-15, it is difficult to interpret the RNAseq data presented for these clusters. Finally, TNF activate IRF-1, which promotes type I IFN expression. This is likely the mechanism of the increase in IFNs.

Figure 4. The experiments and results in this figure have been previously published using many cell types and do not explain the changes in cell clusters in HPC treated with TNF, especially since this was not evaluated in the setting of

inhibitors.

Figure 5. The data depicted demonstrate that CXCL10 protein can be found in the media of TNF-treated HPCs. This also does not explain the effect of TNF on HPC survival or differentiation and does not identify the cellular source of the chemokine, which is likely to be astrocytes (see 10.1016/j.it.2020.07.007).

Figure 6. Here the authors show that type I IFN signaling is required for the expression of CXCL10 by TNF-treated HPCs via a previously published pathway (see 10.1128/JVI.02007-13).

Figure 7. The effect of TNF in this experiment does not match data shown in Figure 1. What dose of TNF was used? Why were different statistical tests used to examine MAP2 versus DCX? Of note, the suppression of neurogenesis by type I IFN has previously been published (see 10.1016/j.stemcr.2014.05.015).

Reviewer #2

(Remarks to the Author)
In this study, Nissen et al. explored how chronic TNF- signaling suppresses human hippocampal neurogenesis in an established in vitro model system. They report here that chronic TNF- exposure limits neurogenesis and induces robust type I IFN expression as well as the secretion of the chemokine CXCL10, which is involved in T cell recruitment. They further showed that IFNAR and JAK signaling are critically involved in directing T cell chemotaxis and DCX+ neuroblast loss in response to chronic TNF- treatment in their human hippocampal progenitor cell in vitro model system. Overall, I am enthusiastic about this work; however, there are a few specific areas that should be addressed to further bolster the impact, scope, and rigor of their work.

Specific comments:
1. Additional information is needed to introduce what rationale was used to arrive on the TNF- concentrations that were used in their studies and to describe if these amounts are physiologically relevant to levels that could be encountered in the brain.
2. Zoomed-in images or insets depicting differences in nuclear translocation of NF- B p65 would be helpful in Figure 1a, as it is currently challenging to differentiate between the total green staining versus the green staining found specifically in the nucleus.
3. It is a little unclear how exactly chronic TNF- signaling leads to type I IFN production at the mechanistic level. It would be interesting to at least take a stab at defining this. One potential scenario is that chronic TNF- stimulation leads to mitochondrial compromise and the release of mitochondrial DNA into the cytoplasm, which could then trigger cGAS/STING-dependent type I IFN production. At the very least, it would be of interest, and also help to bolster the impact and significance of this work, if they ascertain whether the type I IFN production/signaling that is key to their model is dependent on cGAS/STING, especially given the availability of cGAS/STING inhibitors in the field. Even if TNF- -driven induction of type I IFN is not dependent on cGAS/STING that would still be interesting and of value in the field.
4. To increase the scope and impact of their work, it would be valuable to evaluate whether the increased CXCL10 production that they observe downstream of TNF- treatment is dependent on IFNAR and JAK signaling as this part of their model does not appear to have been thoroughly probed in their studies and would be helpful to connect to their T cell chemotaxis and CXCR3 findings, especially given that CXCR3 is known to respond to multiple chemokines (i.e., CXCL9, CXCL10, and CXCL11).

Reviewer #3

(Remarks to the Author)
This study shows that chronic TNFa shifts human hippocampal progenitor cells from generating neurons to adopting immune-related functions. It uncovers the molecular mechanism driving this switch and explains the emergence of type I interferon signaling in aging and neurodegeneration, where TNF-α is elevated. The findings also suggest that HPCs may directly contribute to chronic inflammation.
The study is very well performed, highly significant to the field, and results are well discussed. There are still some issues that should be addressed:

- TNFa has been shown to trigger cell death. In the imaging figures, the higher TNFa dose seems to lead to a decrease in the number of nuclei (eg. Fig1F and Sup Fig1). A quantitative analysis of the total number of cells in each dose of TNFa, and also in the chronic model of TNF administration, should be presented and discussed.
- NK-kB translocation is shown only for 10ng/mL of TNFa (Fig1A and B), but there is expression of NF-kB-regulated chemokines and cytokines at lower doses (Fig1C and D). It would be interesting to show the same NF-kB data for the other TNFa doses.
- Does any of the conditions tested induces cell death?
- The authors performed scRNA sequencing of HPCs exposed for 48 hours to low (0.1 ng/ml) or high (1 ng/ml) TNFa. Why they did not perform with the highest dose of 10 ng/ml, as they showed a more potent effect for example on the percentage of cells expressing MAP2 and DCX (and neurite length)?
- Still regarding the scRNA data, the astrocyte-like clusters also show a remarkable reduction at high-dose TNFa. How do you explain this and what can be the consequences/contribution to the results obtained in this study? Please discuss these

results further.
- When testing the IFNAR inhibition in the transwell assays, an isotype control of Anifrolumab should be included (similarly to what was presented in Figure 4G). Also, in Sup Fig. 19, the isotype control should be included;
- Please discuss to what extent the TNFa doses tested (both in the acute and chronic protocols) may mimic what might be observed in vivo
- Please discuss the situations when the accumulation of TNFa might happen in vivo

Minor reviews:
- Line 172, should be fig 4c and not 4b
- Line 189, the Sup Fig. 14 is not well referenced - it does not refer to BST-2 expression
- Sup Fig18b, y-axis should be MFI and not FMO

Reviewer #4

(Remarks to the Author)
I co-reviewed this manuscript with one of the reviewers who provided the listed reports. This is part of the Nature Communications initiative to facilitate training in peer review and to provide appropriate recognition for Early Career Researchers who co-review manuscripts.

Version 1:

Reviewer comments:

Reviewer #1

(Remarks to the Author)
The authors have adequately address most of my comments. However, I still have a few questions based on their new data.

1. The finding that pyrogens induce glial cells to adopt a proinflammatory reactive state has been shown by others and should also be cited (10.1016/j.bbi.2021.10.010). This process requires upregulation of transcription factor nuclear factor IA and activation of STAT3. Do the authors find this is true in their models?

2. In the revised manuscript, the authors examine caspase 3 (C3) to determine if TNF causes apoptosis. Although they find that TNF has not effect on the %C3+ cells, this does not mean that the TNF is not causing cell death in their model, as TNF can also trigger caspase-independent necrosis or necroptosis via RIPK1/RIPK3. Thus, it is still possible that the loss of clusters (or cells) without increases in progenitors or alternative lineages are due to cell death. In addition, if the neurogenic cells express the lowest amount of TNFR1, how is TNF signaling responsible for these effects?

(Remarks on code availability)

Reviewer #2

(Remarks to the Author)
The authors have done a fine job of addressing my comments and critiques.

(Remarks on code availability)

Reviewer #3

(Remarks to the Author)
This study demonstrates that chronic TNF-α exposure redirects human hippocampal progenitor cells (HPCs) from a neurogenic fate toward immune-related functions. The authors identify the molecular mechanisms underlying this shift and provide a compelling explanation for the emergence of type I interferon signaling observed in aging and neurodegenerative conditions, where TNF-α levels are elevated. Importantly, the findings suggest that HPCs may not merely be passive targets of inflammation but could actively contribute to the maintenance of chronic inflammatory states.

The study is technically rigorous, conceptually innovative, and highly significant for the field of neuroinflammation and neurogenesis. The experiments are carefully performed, and the results are clearly presented and thoughtfully discussed within the broader context of aging and neurodegeneration.

The revised manuscript has substantially improved and appropriately addresses the main concerns raised in the previous review. The clarifications and additional analyses strengthen both the mechanistic conclusions and the overall impact of the

study. I find the revisions satisfactory and believe the manuscript is now suitable for publication.

(Remarks on code availability)

Reviewer #4

(Remarks to the Author)
I co-reviewed this manuscript with one of the reviewers who provided the listed reports. This is part of the Nature Communications initiative to facilitate training in peer review and to provide appropriate recognition for Early Career Researchers who co-review manuscripts.

(Remarks on code availability)

Version 2:

Reviewer comments:

Reviewer #1

(Remarks to the Author)
The authors have adequately addressed all my concerns.

(Remarks on code availability)

Open Access This Peer Review File is licensed under a Creative Commons Attribution 4.0 International License, which permits use, sharing, adaptation, distribution and reproduction in any medium or format, as long as you give appropriate credit to the original author(s) and the source, provide a link to the Creative Commons license, and indicate if changes were made.
In cases where reviewers are anonymous, credit should be given to 'Anonymous Referee' and the source.
The images or other third party material in this Peer Review File are included in the article's Creative Commons license, unless indicated otherwise in a credit line to the material. If material is not included in the article's Creative Commons license and your intended use is not permitted by statutory regulation or exceeds the permitted use, you will need to obtain permission directly from the copyright holder.
To view a copy of this license, visit https://creativecommons.org/licenses/by/4.0/

Reply to Reviewers' comments for the manuscript "TNF-α induces type I IFN signalling to suppress neurogenesis and recruit T cells" by Nissen et al.

We thank the Reviewers for their careful evaluation of our manuscript and for their constructive comments and suggestions, which have further strengthened the study.

We appreciate the positive overall assessment of the work; as noted by Reviewer 2, "Overall, I am enthusiastic about this work," and by Reviewer 3, who stated that "The study is very well performed, highly significant to the field, and results are well discussed."

In response to the Reviewers' thoughtful feedback, we have thoroughly revised the manuscript with new experiments and analyses. In total, we have produced 23 new/updated figure panels distributed throughout the main manuscript and the supplementary information. We believe these changes fully address all comments to further enhance clarity, rigor, and impact of our manuscript. These modifications are clearly indicated using tracked changes in the revised manuscript.

Below, we provide a detailed, point-by-point response to each comment.

- Reviewer comments are shown in **black bold font**, and our responses are shown in blue font.
- New text inserted in the manuscript is shown in black font in the rebuttal (with line numbers based on manuscript 'All Mark-up' display)
- References below are cited in author–year format to avoid confusion with the manuscript's numbered reference list.

Reviewer #1:

General comments:

Nissen et al report on studies evaluating the effect of TNF on human hippocampal progenitor cells (HPCs). They used two doses of the cytokine, and found that chronic exposure, especially at high dose, led to loss of neuroblasts and neurons, which is validated via immunocytochemistry. They present scRNAseq data derived from cell

clusters of HPCs treated with TNF at different time-points and doses. The authors highlight changes in IFNAR signaling pathways, including cytokine and chemokine mRNAs and conclude that TNF suppresses neurogenesis and promotes expression of inflammatory molecules. *A major problem with this conclusion is that the authors fail to evaluate TNF receptors in their clusters and do not explore the vulnerability of different cells to TNF-mediated cell death. Indeed, much of the high dose TNF data indicates loss of clusters (or cells) without increases in progenitors or alternative lineages. Without examination of this alternative hypothesis, the data cannot support the conclusions of the authors.* Finally, the authors perform a number of experiments that are tangential to their original goals and depict known pathways that regulate type I IFN expression and detection of bioactive, IFNAR-inducible CXCL10 in the media of TNF-treated HPCs, the latter of which was not traced to any specific cell type. *The authors finally perform an experiment in which they block IFNAR and demonstrate less TNF-mediated decrease in percentages of DCX+ cells. However, the suppression of neurogenesis by type I IFN has previously been published by others (see 10.1016/j.stemcr.2014.05.015).*

We thank the reviewer for these important comments. We agree that TNF-α can trigger TNFR1-dependent cell death in certain contexts; therefore, we have 1) reanalysed our scRNA-seq data demonstrating TNF receptor expression across clusters and 2) generated new experimental data investigating whether TNF-α alters cell numbers and/or apoptosis. Briefly, the cluster-resolved TNF receptor expression data (Supplementary Fig. 9) showed *TNFRSF1A* was expressed across clusters with the neurogenic cluster having the lowest percentage of TNFRSF1A+ cells. This argues against selective loss of a TNFR1-high, apoptosis-prone neurogenic population (see point 2.2 below). In the new experimental data, we did not detect a decrease in cell numbers or increase in apoptosis in response to TNF-α (Supplemental Fig. 7). with the concentrations and treatment regimens used for the scRNA-seq data (see point 2.1 below). Moreover, our scRNA-seq data indicates that chronic TNF-α exposure biases differentiating HPCs away from homeostatic neurogenic and astrocytic programs and towards reactive, IFN-responsive astroglial states (see Reviewer 3, point 5). This is reflected in the emergence of clusters 13-15, which we renamed IFN-responsive glial/progenitor-like, reactive astrocyte-like, and wound healing-like (see

point 3). Together, these new data support our conclusion that TNF-α predominantly alters HPC state and differentiation through transcriptional reprogramming as compared to selective TNFR1-mediated apoptosis.

As noted in the original manuscript (lines 557-564), prior studies have demonstrated that type I IFNs impair adult hippocampal neurogenesis, a conclusion that is fully consistent with and reinforced by our findings. Importantly, the novelty of our study does not lie in re-establishing this phenotypic effect, but rather in the identification and experimental validation of type I IFN signalling as the central mechanistic pathway mediating the antineurogenic effects of TNF-α and driving TNF-α-induced CXCR3-dependent T cell chemotaxis. Thus, our work provides a unifying mechanistic framework that explains how TNF-α exerts its deleterious effects on neurogenesis through type I IFN signalling.

Below we now address each specific comment:

Specific comments:

1. Introduction lacks inclusion of literature demonstrating effects of viruses on adult neurogenesis in humans and mice. See:

1. (2024) Nat Immunol. Jul;25(7):1158-1171. (mouse)
2. (2022) Brain Aug 25:awac270.
3. (2018) Nat. Immunol., Feb;19(2):151-161
4. (2019) Stem Cells. Nov;37(11):1467-1480
5. (2016) Cell Stem Cell. Nov 3;19(5):593-598.

We thank the reviewer for this helpful suggestion. We have now incorporated the recommended literature demonstrating the effects of viral infection on adult hippocampal neurogenesis (Garber et al., 2018; Li et al., 2016; Li Puma et al., 2019; Soung et al., 2022; Vanderheiden et al., 2024), which contextualises our study within prior work linking infection and impaired neurogenesis. Specifically, the term “viral infection” has been inserted in lines 47 and 56, and a new sentence has been inserted in line 52 – 55 stating that:

"Viral infections, including Zika virus, Herpes Simplex Virus type 1 (HSV-1), and SARS-CoV-2, have been shown to impair AHN in humans and mice by directly infecting NSCs or by inducing neuroinflammatory responses[16,17,20-22]".

2.1 Figure 1. The decrease in DAPI staining with pyknotic nuclei in panel f suggests dose-dependent cell death in response to TNF. As TNF can induce apoptosis, the authors should evaluate this in their model.

We thank the reviewer for this important comment. To directly address whether TNF-α induced apoptosis in this system, we generated *new data* using immunocytochemistry combined with high-content imaging of nuclear cleaved caspase-3 (CC3) (See new Supplementary Fig. 7).

At 24 h, TNF-α treatment (0.1, 1, or 10 ng/ml) did not alter the DAPI+ cell number or the percentage of CC3+ cells. In the chronic differentiation paradigm (48 h TNF-α treatment during proliferation followed by chronic exposure for seven days of differentiation), 10 ng/ml TNF-α reduced the DAPI+ cell number, but TNF-α had no effect on the percentage of CC3+ cells. Together, these data indicate that TNF-α does not induce CC3-dependent apoptosis in HPCs and their differentiated progeny at the analysed time points. The observed reduction in cell number may therefore reflect reduced proliferation, caspase-independent cell death, or early loss of apoptotic cells before fixation. Importantly, at the concentrations used in our key experiments including the scRNA-seq analysis (Figure 2-3), the mechanistic work (Figure 4), the chemotaxis assay (Figure 5-6), and the anifrolumab rescue experiment (Figure 7), we did not detect reduced cell numbers or increased apoptosis following acute or chronic TNF-α exposure. This finding has now been reported in line 112 – 120:

"As TNFR1 signalling can promote apoptosis, we examined whether TNF-α induced apoptotic cell death in HPCs. Acute TNF-α treatment (24 h) did not affect the number of DAPI$^+$ proliferating HPCs, whereas chronic exposure to 10 ng/ml TNF-α during differentiation reduced the number of DAPI$^+$ cells. However, neither acute TNF-α treatment of proliferating HPCs nor chronic TNF-α exposure during differentiation

increased the proportion of cells positive for nuclear cleaved caspase-3 (CC3) (Supplementary Fig. 7b,d). Thus, the reduced number of DAPI$^+$ cells following chronic exposure to 10 ng/ml TNF-α during differentiation may reflect reduced proliferation, caspase-independent cell death, or early loss of apoptotic cells before fixation."

In parallel to the imaging data, we reanalysed the scRNA-seq dataset scoring all clusters against an apoptosis gene signature and a programmed cell death gene signature (Supplementary Fig. 10a-b). Neither signature was elevated in the high-dose TNF-α-specific clusters. Likewise, grouping cells by treatment showed no increase in apoptosis or programmed cell death gene signature in response to TNF-α (Supplementary Fig. 10c-d). Because scRNA-seq quality control removes cells with high mitochondrial read fractions, potentially excluding dying cells, we examined mitochondrial read percentages prior to filtering. We observed no dose-dependent increase in cells with >15% mitochondrial reads at any time point in response to TNF-α, indicating no hidden increase in stressed or apoptotic cells before quality control.

2.2 Also, there are no data demonstrating TNF receptors. It is important to show which clusters express these receptors, which induce different signaling pathways upon TNF binding with TNFR1 promoting death pathways via caspase activation. Thus, the conclusion that TNF reprograms HPCs is not supported by this data.

The original manuscript demonstrated TNFR1 and TNFR2 protein expression in control and TNF-α-treated HPCs (Supplementary Fig. 1), consistent with prior work (Johansson et al., 2008). In response to the reviewer, we now provide cluster-resolved analysis of *TNFRSF1A* (encoding TNFR1) and *TNFRSF1B* (encoding TNFR2) expression in the scRNA-seq dataset (Supplementary Fig. 9). *TNFRSF1A* was expressed across all clusters, with the lowest proportions in the neuroblast-like and immature neuronal-like clusters (11.2% and 7.5% respectively), arguing against selective TNFR1-mediated apoptotic vulnerability in neuronal lineage clusters. We added the following text to the results section on line 178-184:

“Given the potential for TNFR1-mediated cell death, we assessed TNFR1 receptor expression and apoptosis/programmed cell death signatures. *TNFRSF1A* was detected across all clusters but least frequently detected in the neuroblast-like and immature neuron-like clusters (11.2% and 7.5%) (Supplementary Fig. 9), and apoptosis/programmed cell death signature scores were not increased with high-dose TNF-α-treated cells or in high-dose TNF-α-specific clusters (Supplementary Fig. 10).”

3. Figure 2. From which time-points were the UMAP and panels d-h created? Please include the identities of the 15 clusters in the figure, particularly their color coding in panel c. Panel d Y axis is too jumbled to interpret. What are clusters 13-15?

Thank you for requesting these clarifications.

Time-points: The UMAP plots in Figures 2b and 2e represent cells from all three time points (48 hours of proliferation, seven days of differentiation, and fourteen days of differentiation). Extended Figure 1a shows the UMAP plot split by treatment and time point.

Cluster identities: Cluster 13, **14**, and **15** consisted primarily of cells differentiated in the presence of high-dose TNF-α (1 ng/ml). **Cluster 13** was mainly composed of cells differentiated for 7 days, whereas **cluster 14 and 15** were predominantly composed of cells differentiated for 14 days (Figure 2c). The cells in these clusters did not express canonical neuroblast or neuronal markers (Figure 2d).

Cluster 13 expressed *CLU*, *ID4*, *PTN*, and *CD44,* suggesting a glial/progenitor-like transcriptional phenotype. In addition, this cluster displayed strong upregulation of type I IFN-response genes (*ISG15*, *MX1*, *STAT1)* as well as genes upregulated in reactive astrocytes (*CXCL10*, *HLA-F*, *C3) (Matusova et al., 2023),* indicating an IFN-responsive glial/progenitor like phenotype.

Similarly, **cluster 14** expressed *CLU*, *ID4*, *PTN*, and *CD44,* together with astrocytic lineage markers (*GFAP*, *S100B, AQP4*) and inflammatory/reactive astrocyte-

associated genes (*TIMP1*, *CP, CRYAB) (Matusova et al., 2023)* suggesting a reactive astrocyte-like phenotype.

Finally, **cluster 15** did not express canonical astrocytic markers but did express genes implicated in wound healing (*MYLK*, *HBEGF*, *CD151*) which have recently been found in dedifferentiated and transcriptionally reprogrammed astrocytes following CNS injury (O'Shea et al., 2024).

Accordingly, we designated **cluster 13** 'IFN-responsive glial/progenitor-like', **cluster 14** 'reactive astrocyte-like', and **cluster 15** 'wound healing like'.

To address the reviewers' comments, we have:

1) Inserted the following paragraph describing the transcriptional identities of cluster 13-15 on line 195– 213:

"HPCs differentiated in the presence of high-dose TNF-α clustered separately from HPCs differentiated under control and low dose TNF-α treatment. They split into three clusters: an IFN-responsive glial-progenitor-like cluster, a reactive astrocyte-like cluster, and a wound healing-like cluster. The IFN-responsive glial-progenitor-like cluster expressed *CLU*, *ID4*, *PTN*, and *CD44* suggesting a glial/progenitor-like transcriptional phenotype and displayed strong upregulation of type I IFN-response genes (*ISG15*, *MX1*, *STAT1).* Similarly, the reactive astrocyte-like cluster expressed *CLU*, *ID4*, *PTN*, and *CD44,* together with astrocytic lineage markers (*GFAP*, *S100B, AQP4*) and inflammatory/reactive astrocyte-associated genes (*TIMP1*, *CP, CRYAB)*[48] suggesting a reactive astrocyte-like phenotype. Finally, the wound-healing-like cluster did not express canonical astrocytic markers but expressed genes implicated in wound healing (*MYLK*, *HBEGF*, *CD151*) which have recently been found in dedifferentiated and transcriptionally reprogrammed astrocytes following CNS injury[49]."

2) Inserted the following sentence to the revised manuscript, line 215 – 217:

“These findings indicate that high-dose TNF-α drives differentiating HPCs away from neurogenic and homeostatic astrocytic transcriptional identities and toward reactive, IFN-responsive glial-like states.”

3) Updated Figure 2d to make it easier to interpret. The colouring is consistent across Figure 2b, Figure 2c, and Figure 2h. Furthermore, we have updated all figures, legends, and results with the new cell cluster names.

Panel h and Supplemental data support the notion that TNF promotes cell death programs, which should be evaluated.

Figure 2h shows a reduction in the proportion of cells assigned to the neuroblast-like and immature neuron-like clusters among HPCs differentiated in the presence of chronic 1 ng/ml TNF-α compared to control-treated cells, consistent with the ICC data presented in Figure 1. Several mechanisms may contribute to this shift; however, the new apoptosis analysis (Supplementary Fig. 7), indicating that apoptosis is unlikely to be the primary driver.

4. Figure 3. The differences in innate immune molecules could be due to loss of clusters or differential responses to TNF depending on which receptors are expressed. Again, experiments examining TNFR1-mediated cell death could explain loss of clusters. In addition, type I IFN responses can enhance TNF-mediated apoptosis in cells that express TNFR1.

We thank the reviewer for these important comments. We acknowledge that in principle, both mechanisms could contribute.

However, our newly generated CC3 imaging data (presented above in response to point #2.1, Figure 1) demonstrate that TNF-α does not increase apoptosis or decrease cell numbers at the concentrations used in the scRNA-seq data. Thus, the cluster composition changes in response to TNF-α are unlikely to be driven by TNFR1-mediated apoptosis.

Furthermore, we performed cluster-resolved analysis of *TNFRSF1A* and *TNFRSF1B* expression (Supplementary Fig. 9). *TNFRSF1A* was expressed across all clusters,

and notably the lowest fraction of *TNFRSF1A*+ cells occurred in the neurogenic clusters. This argues against selective loss of neurogenic clusters through a TNFR1-high, apoptosis-prone subpopulation. Furthermore, the differential induction of innate immune programs is unlikely to be attributed to restricted or cluster-specific TNFR1 expression. Taken together, these data strongly support the conclusion that TNF-α alters cluster composition predominantly through transcriptional reprogramming rather than selective TNFR1-dependent cell loss. We have discussed this on line 525-532:

"Consistent with this, scRNA-seq analysis showed that *TNFRSF1A* was detected across clusters, with the lowest fraction of *TNFRSF1A*+ cells in the neurogenic clusters (Supplementary Fig. 9). Moreover, apoptosis and programmed cell death signatures were not enriched in high-dose TNF-α-treated cells (Supplementary Fig. 10). Together, these observations argue against selective loss of neurogenic clusters via a TNFR1-high, apoptosis-prone subpopulation and support the conclusion that TNF-α reduces neurogenesis predominantly through transcriptional reprogramming."

Although type I IFNs can potentiate TNF-α-mediated apoptosis, we found no evidence of an elevated apoptosis/programmed cell death gene signature in the cell clusters that displayed elevated type I IFN response signature (Figure 3c and Supplementary Fig. 10a-b). We added the following new sentence to the manuscript in line 192-194:

"Although type I IFN signalling can sensitise cells to apoptosis[48], we did not observe enrichment of apoptosis/programmed cell death signatures in clusters with a type I IFN signature (Supplementary Fig. 10).

Also, as the authors do not provide the identify of clusters 13-15, it is difficult to interpret the RNAseq data presented for these clusters.

The transcriptional phenotype of cluster 13 – 15 was described in our response the reviewer's comment (point #3, Figure 2).

Finally, TNF activate IRF-1, which promotes type I IFN expression. This is likely the mechanism of the increase in IFNs.

We agree with the reviewer that TNF-α-induced IRF1 likely drives the *IFNB1* upregulation and subsequent type I IFN-stimulated gene expression which we stated in the original manuscript on line 281-285:

"As IRF1 is required for TNF-α–induced *IFNB1* expression in other cell types[53,53], we examined its regulation in HPCs. TNF-α markedly upregulated *IRF1*, peaking at 3 hours (Supplementary Fig. 15), preceding *IFNB1* induction and suggesting a potential regulatory role for IRF1 in HPC *IFNB1* induction."

5. Figure 4. The experiments and results in this figure have been previously published using many cell types and do not explain the changes in cell clusters in HPC treated with TNF, especially since this was not evaluated in the setting of inhibitors.

We agree with the reviewer that activation of type I IFN signalling by TNF-α has been described in several peripheral cell types, including macrophages, fibroblasts, and endothelial cells, and we have cited these studies in the manuscript (refs 53-55,94). Importantly, however, our study extends this paradigm to the CNS by demonstrating this signalling axis for the first time in hippocampal progenitor cells (HPCs). To our knowledge, this represents the first evidence of TNF-α–driven type I IFN responses in CNS-resident progenitor cells, and more broadly in stem cell populations. To clarify that our work identified this pathway in a new cell type and its functional consequences we have revised line 543 - 545 in the discussion:

"Our findings identify a TNF-α-IFN-β-IFNAR axis in human HPCs, establishing TNF-driven type I IFN activation in a CNS-resident progenitor cell."

Furthermore, we have revised line 491 – 493 in the discussion adding "in HPCs" to clarify that this is not a novel signalling axis, but we are the first to report it is functional in CNS and/or progenitor cells:

"Our data uncovered a previously uncharacterised TNF-α-IFN-β-IFNAR-CXCL10 axis in HPCs that may exist in other CNS-resident cells and its role in reprogramming HPCs

from a neurogenic to an immune-defensive state under chronic inflammatory conditions."

We agree that Figure 4 does not directly address the changes in cell cluster composition observed in response to chronic TNF-α, however, this was *never* the goal of these experiments. The primary purpose of Figure 4 was to validate TNF-α-induced STAT1 activation and to elucidate the underlying signalling mechanism. Given the well-established anti-neurogenic role of STAT1, the scRNA-seq results predicting TNF-α-driven STAT1 activation provided a mechanistic hypothesis for the observed loss of neurogenic clusters. Consequently, it was critical to experimentally confirm STAT1 activation in response to TNF-α and to dissect the responsible signalling cascade. To clarify the intent of Figure 4 we revised the context of the text - see lines 251 – 253 in the results section:

"As STAT1 negatively regulates human hippocampal neurogenesis[51] and STAT1/STAT2 regulates the expression of type I IFN-regulated genes, we next assessed their activation in HPCs following TNF-α stimulation and investigated the underlying mechanism."

The functional relevance of this pathway is demonstrated in Figure 6 and 7, where IFNAR blockade prevents TNF-α-induced T-cell chemotaxis and neuroblast loss. Together, these findings provide a mechanistic explanation for the transcriptional phenotype observed in the scRNA-seq data, including upregulation of CXCL10 (see below) and reduction in neurogenic clusters.

6. Figure 5. The data depicted demonstrate that CXCL10 protein can be found in the media of TNF-treated HPCs. This also does not explain the effect of TNF on HPC survival or differentiation and does not identify the cellular source of the chemokine, which is likely to be astrocytes (see 10.1016/j.it.2020.07.007).

We thank the reviewer for this insightful comment. We agree with the reviewer that CXCL10 is likely a major contributor to the chemotactic T cell response based on the concentrations identified in the LEGENDplex data (Supplementary Fig. 5).

We agree that Figure 5 does not address the mechanisms underlying HPC survival or differentiation. Its purpose was to determine whether proliferating and differentiating HPCs secrete bioactive CXCR3 ligands at sufficient concentrations to drive T cell chemotaxis in response to TNF-α.

To identify the cellular source of these chemokines, we reanalysed the scRNA-seq dataset for *CXCL9*, *CXCL10*, and *CXCL11*. The data reveal *CXCL9* expression was not detected, whereas *CXCL10* and *CXCL11* were highly expressed in the IFN-responsive glial/progenitor-like cluster. These new data are shown in Supplementary Figure 19 and are consistent with the reviewer's suggestion, as well as with prior evidence that reactive astrocytes are major producers of CXCL10 (Giovannoni and Quintana, 2020). Thus, the scRNA-seq analysis supports the interpretation that TNF-α induces CXCR3 ligand production primarily in astrocyte-like reactive states arising under chronic inflammatory conditions. We have revised the results section relating to Figure 5 on line 379 – 386 addressing the cellular source of the CXCR3-ligands:

"These findings indicate that TNF-α–treated HPC cultures (during proliferation and differentiation) release CXCR3 ligands at concentrations sufficient to promote T cell chemotaxis. To infer the cellular source of these CXCR3 ligands, we re-analysed the scRNA-seq dataset and found that CXCL9 was not detected, whereas CXCL10 and CXCL11 were enriched in an IFN-responsive glial/progenitor-like cluster (Supplementary Fig. 19), consistent with prior evidence that reactive astrocyte states are major producers of CXCL10 in the inflamed CNS[64]"

7. Figure 6. Here the authors show that type I IFN signaling is required for the expression of CXCL10 by TNF-treated HPCs via a previously published pathway (see 10.1128/JVI.02007-13).

We thank the reviewer for drawing our attention to this reference. We would like to clarify, however, that the cited study (Brownell et al., 2014; 10.1128/JVI.02007-13; PMID: 24257594) does not demonstrate a requirement for type I IFN signalling in TNF-α–induced CXCL10 expression. In that study, CXCL10 regulation was examined in immortalized hepatoma cells using a constitutively active IRF3 mutant, and the authors

demonstrated IRF3-driven CXCL10 transcription that was independent of type I IFN signalling.

This mechanism therefore differs fundamentally from the pathway described in our manuscript, in which TNF-α drives CXCL10 secretion via a type I IFN–dependent mechanism in HPCs. Moreover, whereas Brownell et al. investigated CXCL10 induction downstream of direct IRF3 activation, our study specifically delineates CXCL10 secretion in response to TNF-α stimulation. These represent distinct upstream modes of pathway activation leading to CXCL10 expression.

While type I IFN induction downstream of TNF-α has been reported in certain peripheral cell types, including endothelial cells (Venkatesh et al., 2013), fibroblast-like synoviocytes (Bonelli et al., 2019), and macrophages (Yarilina et al., 2008), this pathway has not been demonstrated (to the best of our knowledge) in CNS-resident cells or neural progenitor populations. Our data therefore extend these prior observations to human HPCs and further demonstrate that this signalling axis has functional consequences by promoting T cell chemotaxis and regulating the generation of neuroblasts.

8. Figure 7. The effect of TNF in this experiment does not match data shown in Figure 1. What dose of TNF was used? Why were different statistical tests used to examine MAP2 versus DCX? Of note, the suppression of neurogenesis by type I IFN has previously been published (see 10.1016/j.stemcr.2014.05.015).

We thank the reviewer for catching the missing unit in the legend for Figure 7. The TNF-α concentration used was 1 ng/ml, and we have corrected the legend accordingly:

"Representative images showing the expression of MAP2 (green) and DCX (orange) in seven-day differentiated HPCs treated chronically ± 1 ng/ml TNF-α ± 10 μg/ml anifrolumab. Scale bar represents 100 μm."

The observed effect of chronic exposure to 1 ng/ml TNF-α in Figure 7 is consistent with the findings in Figure 1: notably, a significant reduction in the percentage of DCX+

cells and no effect on the percentage of MAP2+ cells. Furthermore, the overall percentages of MAP2+ and DCX+ cells under control and TNF-α conditions are comparable across the two figures, supporting the reproducibility of the effect.

The difference in statistical tests between Figured 7b and 7c reflects the outcomes of their respective assumption tests. The data in Figure 7b violated normality and showed unequal variances. Therefore, as stated in the Statistical Analysis section, we applied the non-parametric Kruskal-Wallis test followed by Dunn's multiple comparisons. In contrast, the data in Figure 7c met parametric assumptions and were therefore analysed using a one-way ANOVA with Bonferroni's post hoc correction.

We thank the reviewer for pointing out this reference (Zheng et al., 2014), which was already cited in the original manuscript. As discussed in the original manuscript on line 568-569, prior work has shown that type I IFNs impair adult hippocampal neurogenesis, a finding that is further supported by our data. The *novelty of our study* lies in identifying and experimentally validating type I IFN signalling as the key mechanistic pathway mediating the antineurogenic effects of TNF-α. We have added the reference (Zheng et al., 2014) paper to our discussion on line 568-569:

"Elevated type I IFNs impair AHN[51,67,68] and promote cognitive deficits[68,100,102] both of which can be reversed through IFNAR antagonism[68,100]."

Reviewer #2:

In this study, Nissen et al. explored how chronic TNF-α signaling suppresses human hippocampal neurogenesis in an established in vitro model system. They report here that chronic TNF-α exposure limits neurogenesis and induces robust type I IFN expression as well as the secretion of the chemokine CXCL10, which is involved in T cell recruitment. They further showed that IFNAR and JAK signaling are critically involved in directing T cell chemotaxis and DCX+ neuroblast loss in response to chronic TNF-α treatment in their human hippocampal progenitor cell in vitro model system. Overall, I am enthusiastic about this work; however, there are a few specific areas that should be addressed to further bolster the impact, scope, and rigor of their work.

1. Additional information is needed to introduce what rationale was used to arrive on the TNF-a concentrations that were used in their studies and to describe if these amounts are physiologically relevant to levels that could be encountered in the brain.

We thank the reviewer for this important comment. We have addressed the rationale for choosing the TNF-α concentrations in the revised manuscript by introducing the below paragraph in the results section line 88-92:

"To model chronic neuroinflammatory TNF-α exposure, we treated proliferating and differentiating HPCs with 0.1-10 ng/ml TNF-α, which is within or below the concentration range used in prior studies evaluating the effect of TNF-α on neural stem/progenitor cells[31,42-46]"

We have now discussed their physiological relevance on line 511-520:

"Direct measurements of TNF-α in the human hippocampal interstitial space during neuroinflammatory conditions are lacking, which is a limitation when extrapolating *in vitro* dosing to the *in vivo* context. CSF TNF-α levels in neurodegenerative disease range from low to high pg/ml[75,76]. However, CSF represents a global compartment and may not reflect focal parenchymal concentrations in inflammatory niches. Activated microglia are a major source of TNF-α in the inflamed hippocampus[77,78]. *In vitro* studies of activated primary human microglia and human iPSC-derived microglia report TNF-α secretion ranging from high pg/ml to double-digit ng/ml depending on stimulus[79-87]. This suggests that ng/ml concentrations are pathophysiologically plausible in focal inflammatory microenvironments."

2. Zoomed-in images or insets depicting differences in nuclear translocation of NF-kB p65 would be helpful in Figure 1a, as it is currently challenging to differentiate between the total green staining versus the green staining found specifically in the nucleus.

We thank the reviewer for this helpful suggestion. We have updated Fig. 1a-b with the new data requested by reviewer 3, point #2. The updated figure includes zoomed-in versions to better visualise the nuclear translocation of NF-κB p65 in HPCs treated ±

TNF-α. The revised figure more clearly illustrate the translocation of NF-κB p65 from the cytoplasm to the nucleus.

3. It is a little unclear how exactly chronic TNF-a signaling leads to type I IFN production at the mechanistic level. It would be interesting to at least take a stab at defining this. One potential scenario is that chronic TNF-a stimulation leads to mitochondrial compromise and the release of mitochondrial DNA into the cytoplasm, which could then trigger cGAS/STING-dependent type I IFN production. At the very least, it would be of interest, and also help to bolster the impact and significance of this work, if they ascertain whether the type I IFN production/signaling that is key to their model is dependent on cGAS/STING, especially given the availability of cGAS/STING inhibitors in the field. Even if TNF-a-driven induction of type I IFN is not dependent on cGAS/STING that would still be interesting and of value in the field.

We agree with the reviewer that exploring the mechanism underlying TNF-α-induced *IFNB1* expression is important. To assess whether the cGAS-STING pathway contributes to this response in HPCs, we first examined the expression of *CGAS* and *STING1* (encoding STING) in our scRNA-seq dataset. Fewer than 1% of control or TNF-α-treated HPCs expressed *CGAS* (Supplementary Figure 14a), moreover, we detected no expression of *STING1,* suggesting that the cGAS-STING pathway is transcriptionally absent in the HPCs. This low expression may reflect their c-Myc-driven immortalisation, as Myc has been shown to repress STING (Lee et al., 2022; Linstra et al., 2025).

We next investigated whether HPCs are functionally responsive to STING activation and whether TNF-α–induced IFNB1 and ISG15 expression is STING dependent. HPCs were stimulated for 3 h with TNF-α (1 ng/mL) or the STING agonist diABZI (100 ng/mL). TNF-α robustly induced *IFNB1* and *ISG15* expression; however, despite the batch of diABZI being validated as functional in THP1 cells, it had no effect on either gene in HPCs (new data in Supplementary Fig. 14b-c). Together with the absence of detectable CGAS and STING1 expression, the lack of responsiveness to diABZI indicates that HPCs do not possess a functional cGAS–STING pathway. Accordingly, TNF-α–induced *IFNB1* expression in this system is independent of cGAS–STING signalling.

These new findings are consistent with our results reported in the manuscript, showing that TNF-α-induced *IFNB1* expression is NF-κB dependant, but independent of *de novo* protein synthesis, and may involve IRF1, which is required for TNF-α-induced *IFNB1* induction in several other cell types (Venkatesh et al., 2013; Yarilina et al., 2008). Future studies using IRF1-deficient HPCs could clarify whether TNF-α–induced *IFNB1* expression in HPCs proceeds via a similar IRF1-dependent mechanism.

We have added the following paragraph to the results section on line 262-281:

"While the cGAS-STING pathway has been implicated in TNF-α-induced *IFNB1* expression[52] in other cell types, the HPCs did not demonstrate *STING1* expression and expressed only minimal levels of *CGAS* (Supplementary Fig. 14a). Furthermore, they were functionally unresponsive to STING activation (Supplementary Fig. 14b-c), indicating that TNF-α-induced *IFNB1* expression occurs through a cGAS-STING-independent mechanism."

4. To increase the scope and impact of their work, it would be valuable to evaluate whether the increased CXCL10 production that they observe downstream of TNF-a treatment is dependent on IFNAR and JAK signaling as this part of their model does not appear to have been thoroughly probed in their studies and would be helpful to connect to their T cell chemotaxis and CXCR3 findings, especially given that CXCR3 is known to respond to multiple chemokines (i.e., CXCL9, CXCL10, and CXCL11).

We thank the reviewer for the suggestion. We demonstrated a delayed upregulation of *CXCL10* in response to TNF-α (Figure 4d) that was dependent on *de novo* protein synthesis (Figure 4e) consistent with a TNF-α-induced type I IFN loop driving CXCL10 induction via IFNAR and downstream JAK signalling. To further characterise this pathway, we quantified the concentrations of the three CXCR3 ligands CXCL9, CXCL10, and CXCL11 in the supernatant of TNF-α-treated HPCs. TNF-α robustly induced the secretion of CXCL10 and CXCL11 but not CXCL9 (Supplementary Figure 5). Importantly, the genes encoding *CXCL10* and *CXCL11*, but not *CXCL9*, contain interferon-stimulated response elements (ISREs) in their promoter regions, which drive transcription via ISGF3 in response to type I IFNs (Metzemaekers et al., 2018).

Thus, the selective induction of CXCL10 and CXCL11 is consistent with type I IFN-dependent induction mediated through IFNAR and JAK signalling.

Importantly, we also demonstrated that conditioned media from HPCs co-treated with TNF-α and either an IFNAR-blocking antibody or a JAK inhibitor failed to induce CXCR3-dependent T cell chemotaxis (Figure 6). Together, these data demonstrate that TNF-α–induced production of functional CXCR3 ligands, including CXCL10, in HPCs requires IFNAR and JAK signalling. We have emphasised the consistency between the mechanistic work in figure 4 and the figure 6 on line 498 - 401:

"These results indicated that the secretion of CXCR3 ligands by HPCs in response to TNF-α was dependent on the autocrine/paracrine type I IFN signalling loop, consistent with the delayed and *de novo* protein synthesis-dependent upregulation of CXCL10 (Fig. 4d-e)."

Reviewer #3:

This study shows that chronic TNFa shifts human hippocampal progenitor cells from generating neurons to adopting immune-related functions. It uncovers the molecular mechanism driving this switch and explains the emergence of type I interferon signaling in aging and neurodegeneration, where TNF-α is elevated. The findings also suggest that HPCs may directly contribute to chronic inflammation. The study is very well performed, highly significant to the field, and results are well discussed. There are still some issues that should be addressed:

1. TNFa has been shown to trigger cell death. In the imaging figures, the higher TNFa dose seems to lead to a decrease in the number of nuclei (eg. Fig1F and Sup Fig1). A quantitative analysis of the total number of cells in each dose of TNFa, and also in the chronic model of TNF administration, should be presented and discussed.

We thank the reviewer for this comment. We have since undertaken a quantitative analysis of total DAPI+ cell numbers across all TNF-α doses including for the chronic differentiation exposure paradigm (new data, Supplementary Fig. 7a,c). After 24 hours of treatment, we did not observe a decrease in DAPI+ cell numbers in response to any

of the TNF-α concentrations. However, after differentiating the HPCs under chronic exposure to 10 ng/ml we observed a decrease in the DAPI+ cell numbers. Therefore, we did not observe reduced cell numbers in response to 0.1 or 1 ng/ml TNF-α, which were used for the key experiments: scRNA-seq analysis (Figure 2-3), the chemotaxis assay (Figure 5-6), and the anifrolumab rescue experiment (Figure 7). We have described these data in the newly introduced results paragraph on line 112 – 120 (see comment from Reviewer 1, point 2.1). We added a point in the discussion on line 522-525:

"Of note, the TNF-α concentrations (0.1 and 1 ng/ml) used for the key experiments (Figs. 2-7) did not reduce total cell numbers or increase CC3+ apoptotic cells (Supplementary Fig. 7), suggesting that decreased neurogenesis and increased inflammatory signalling were unlikely to be driven by increased cell death"

2. NK-kB translocation is shown only for 10ng/mL of TNFa (Fig1A and B), but there is expression of NF-kB-regulated chemokines and cytokines at lower doses (Fig1C and D). It would be interesting to show the same NF-kB data for the other TNFa doses.

We thank the reviewer for this helpful suggestion. We have performed new experiments quantifying the NF-κB p65 nuclear translocation across TNF-α concentrations (0.1, 1, and 10 ng/ml) and time points (0, 15, 30 min, and 3 h). These new data, presented in Figure 1a-b demonstrate dose-dependent NF-κB activation, with significant nuclear translocation at 0.1 ng/ml and 1 ng/ml TNF-α, consistent with the induction of NF-κB-regulated chemokines at these concentrations.

3. Does any of the conditions tested induces cell death?

We have now addressed this point with new experimental data (see response to Reviewer #1, point 2.1). In brief, TNF-α treatment did not increase apoptosis, as measured by the percentage of CC3-positive nuclei.

4. The authors performed scRNA sequencing of HPCs exposed for 48 hours to

low (0.1 ng/ml) or high (1 ng/ml) TNFa. Why they did not perform with the highest dose of 10 ng/ml, as they showed a more potent effect for example on the percentage of cells expressing MAP2 and DCX (and neurite length)?

We selected 0.1 and 1 ng/ml TNF-α for the scRNA-seq experiment because these doses are the most likely to be pathophysiologically relevant and therefore best suited to reveal transcriptional mechanisms operating under biologically meaningful inflammatory conditions. Importantly, both doses produced effects on key readouts central to our hypothesis: CXCL10 secretion and neurogenesis.

Specifically, 0.1 ng/ml TNF-α was sufficient to increase CXCL10 secretion (Figure 1c), whereas 1 ng/ml TNF-α significantly reduced the proportion of DCX+ neuroblasts (Figure 1g). These functional effects were recapitulated in the scRNA-seq dataset: 0.1 ng/ml TNF-α increased the expression of inflammatory genes (Extended data Figure 1b), while 1 ng/ml TNF-α led to a decrease in the proportion of cells within neurogenic clusters (Figure 2h). We have added a comment about this decision in the results section on line 153 – 155:

"These concentrations were selected because they were the lowest doses tested, and therefore the most likely to be pathophysiologically relevant, while still producing effects on key readouts central to our hypothesis: 0.1 ng/ml induced inflammatory output (Fig. 1c), and 1 ng/ml impaired neurogenesis (Fig. 1g)."

5. Still regarding the scRNA data, the astrocyte-like clusters also show a remarkable reduction at high-dose TNFa. How do you explain this and what can be the consequences/contribution to the results obtained in this study? Please discuss these results further.

We thank the reviewer for this important comment. We agree that HPCs differentiated under chronic high-dose TNF-α (1 ng/ml) clustered separately from the astrocyte-like clusters 1-4 composed of control and low-dose TNF-α (0.1 ng/ml) treated cells in the scRNA-seq dataset (Figure 2b-c). Rather than reflecting a loss of astrocytic-lineage cells, our data indicate that high-dose TNF-α drives differentiating HPCs away from

homeostatic astrocytic identities towards reactive, IFN-responsive states. This is reflected in the emergence of clusters 13-15, which we renamed IFN-responsive glial/progenitor-like, reactive astrocyte-like, and wound healing-like.

The potential consequences of such transcriptional astrocytic reprogramming could be a loss of homeostatic astroglial support in functions that normally promote AHN (Cassé et al., 2018). This could contribute to the impaired neurogenesis observed in response to high-dose TNF-α. Second, the reactive, IFN-responsive astrocyte-like states may amplify local inflammatory signalling (including type I IFNs and chemokine secretion), which could further impair neurogenesis and enhance T cell recruitment.

We have now clarified this point in the results section by adding the below to line 215-217:

"These findings indicate that high-dose TNF-α drives differentiating HPCs away from homeostatic astrocytic identities and toward reactive, IFN-responsive glial states."

Furthermore, we have explained and discussed what can be the consequences/contributions by the following text inserted in line 557 - 564:

"To that end**,** our scRNA-seq data indicate that chronic TNF-α promotes a shift from homeostatic astrocyte-like states toward inflammatory/reactive astroglial-like states, which may further amplify the niche inflammatory milieu through increased chemokine production (e.g., CXCL10), thereby reinforcing immune cell recruitment. This shift likely reflects transcriptional reprogramming, consistent with the extensive evidence that inflammatory cues such as TNF-α drive reactive astrocyte programmes[97]. Functionally, such a shift could further impair AHN by reducing homeostatic astroglial support of neurogenesis."

6. When testing the IFNAR inhibition in the transwell assays, an isotype control of Anifrolumab should be included (similarly to what was presented in Figure 4G). Also, in Sup Fig. 19, the isotype control should be included;

An isotype control antibody was included in Figure 4f-h to validate the specificity of anifrolumab in blocking TNF-α-induced type I IFN signalling. These data clearly show that the isotype control antibody had no effect on the proportion of ISG15+ and STAT1+ cells, either alone or in combination with TNF-α. Having established antibody specificity in our experimental system, the isotype control was not included in subsequent experiments to avoid redundancy. In the transwell experiments, anifrolumab and the JAK I inhibitor inhibited T cell chemotaxis to a similar extent, consistent with the specificity of anifrolumab for type I IFN signalling.

7. Please discuss to what extent the TNFa doses tested (both in the acute and chronic protocols) may mimic what might be observed in vivo.

We thank the reviewer for this important point that was also raised by Reviewer 2 in their first comment (#2.1), please see our response to their comment above.

8. Please discuss the situations when the accumulation of TNFa might happen in vivo

We thank the reviewer for this insightful comment. We have now introduced a new paragraph discussing biologically relevant scenarios in which TNF-α accumulation occurs *in vivo,* including ageing, viral infections, neurodegenerative disease, and peripheral inflammatory conditions that drive TNF-α elevation in the CNS. Please see new text, lines 498 – 409of the revised manuscript:

"TNF-α accumulation occurs in multiple pathophysiological contexts in which AHN is disrupted. During ageing, microglia adopt a chronic, low-grade inflammatory phenotype characterised by increased TNF-α expression[69,70]. CNS viral infections can similarly elevate TNF-α; for example, increased hippocampal TNF-α persists following West Nile virus infection, associated with reduced neurogenesis[22]. Activated microglia secrete TNF-α in chronic neurodegenerative diseases including AD[71], Parkinson's disease[72], and multiple sclerosis[73]. Moreover, chronic peripheral inflammation can propagate to the brain, as demonstrated by increased hippocampal TNF-α and impaired neurogenesis during chronic intestinal inflammation[74]. Together, these

findings highlight that CNS TNF-α accumulation is a common feature of diverse chronic inflammatory states, supporting the pathophysiological relevance of our chronic exposure model and identifying TNF-α as a potential convergent regulator of AHN across disease contexts."

Minor comments:

1. Line 172, should be fig 4c and not 4b.

We thank the reviewer for catching this error which is corrected in the new version of the manuscript.

2. Line 189, the Sup Fig. 14 is not well referenced - it does not refer to BST-2 expression.

We thank the reviewer for highlighting this issue. The text has been updated to:

"Finally, TNF-α upregulated the type I IFN-induced surface marker tetherin/BST-2 on HPCs (Extended Fig. 2i), which was recently shown to be elevated on dysfunctional NSCs in ageing neurogenic niches."

3. Sup Fig18b, y-axis should be MFI and not FMO.

We thank the reviewer for highlighting this issue. The y-axis labels have been updated to "MFI ICAM1" and "MFI ICAM1" in the revised version of Supplementary Fig. 21b.

References cited in this rebuttal (only):

1. Brownell, J. *et al.* Direct, Interferon-Independent Activation of the CXCL10 Promoter by NF-κB and Interferon Regulatory Factor 3 during Hepatitis C Virus Infection. *J Virol* 88, 1582–1590 (2014).
2. Venkatesh, D. *et al.* Endothelial TNF Receptor 2 Induces IRF1 Transcription Factor-Dependent Interferon-β Autocrine Signaling to Promote Monocyte Recruitment. *Immunity* 38, 1025–1037 (2013).
3. Bonelli, M. *et al.* IRF1 is critical for the TNF-driven interferon response in rheumatoid fibroblast-like synoviocytes. *Exp Mol Med* 51, 1–11 (2019).

4. Yarilina, A., Park-Min, K.-H., Antoniv, T., Hu, X. & Ivashkiv, L. B. TNF activates an IRF1-dependent autocrine loop leading to sustained expression of chemokines and STAT1-dependent type I interferon–response genes. *Nat Immunol* 9, 378–387 (2008).

Reply to Reviewers' comments for the manuscript "TNF-α induces type I IFN signalling to suppress neurogenesis and recruit T cells" by Nissen et al.

We thank the Reviewers for their time and careful evaluation of our revised manuscript.

We are encouraged that **Reviewer 3** characterized the study as "*technically rigorous, conceptually innovative, and highly significant for the field of neuroinflammation and neurogenesis*," emphasizing that the experiments are "*carefully performed, clearly presented, and thoughtfully contextualized*" and concluding that the revised manuscript "*appropriately addressed the major concerns, strengthened the mechanistic conclusions, and is suitable for publication*". Consistent with this assessment, **Reviewer 2** commented that "*the authors have done a fine job of addressing my comments and critiques*."

Taken together, these independent evaluations indicate that the revisions have substantially strengthened the manuscript and that the study meets the standards of rigor, clarity, and impact expected for publication.

In response to **Reviewer 1's** additional feedback and questions, we have further revised the manuscript and incorporated *new* bioinformatic analyses into the Supplementary Information. We believe these additions, together with our prior revisions, fully address the remaining concerns. All modifications requested by Reviewer 1 are clearly indicated using tracked changes in the revised manuscript.

Below, we provide a detailed, point-by-point response to each comment.

- Reviewer comments are shown in **black bold font**, and our responses are shown in blue font.
- New text inserted in the manuscript is shown in black font in the rebuttal (with line numbers based on manuscript 'All Mark-up' display)
- References below are cited in author–year format to avoid confusion with the manuscript's numbered reference list.

Reviewer #1:

General comments:

The authors have adequately address most of my comments. However, I still have a few questions based on their new data.

1. The finding that pyrogens induce glial cells to adopt a proinflammatory reactive state has been shown by others and should also be cited (10.1016/j.bbi.2021.10.010).

We thank the reviewer for drawing our attention to this study. We have now incorporated this reference into the manuscript and added the following text in the revised discussion (lines 486–490), where we contextualize our findings in relation to these prior observations:

"*Similarly, IL-1β has been reported to reprogram murine glial cells to adopt a proinflammatory, reactive state in the context of viral-driven inflammation, a process involving STAT3 signalling*[99]*. Consistent with this, our transcription factor analysis predicted STAT3 activity in high TNF-α-specific cell clusters (Fig. 3h), suggesting STAT3 may represent a shared regulatory node underlying cytokine-induced proinflammatory astrocyte states*."

This process requires upregulation of transcription factor nuclear factor IA and activation of STAT3. Do the authors find this is true in their models?

As discussed above, our transcription factor analysis predicted STAT3 activity in the cell clusters specific to treatment with high TNF-α (Fig. 3h). Therefore, it is possible that STAT3 is implicated in the TNF-α-induced proinflammatory glial state.

NF-IA was not among the top 50 most variable or enriched transcription factors predicted in our scRNA-seq dataset. Furthermore, transcription factor activity inference analysis indicated only a modest elevation of predicted NF-IA activity within

the IFN-responsive glial/progenitor-like cluster (see below). Importantly, this level was comparable to that observed in the homeostatic astro-like 1 cluster, arguing against selective or functionally significant upregulation in the inflammatory context examined here.

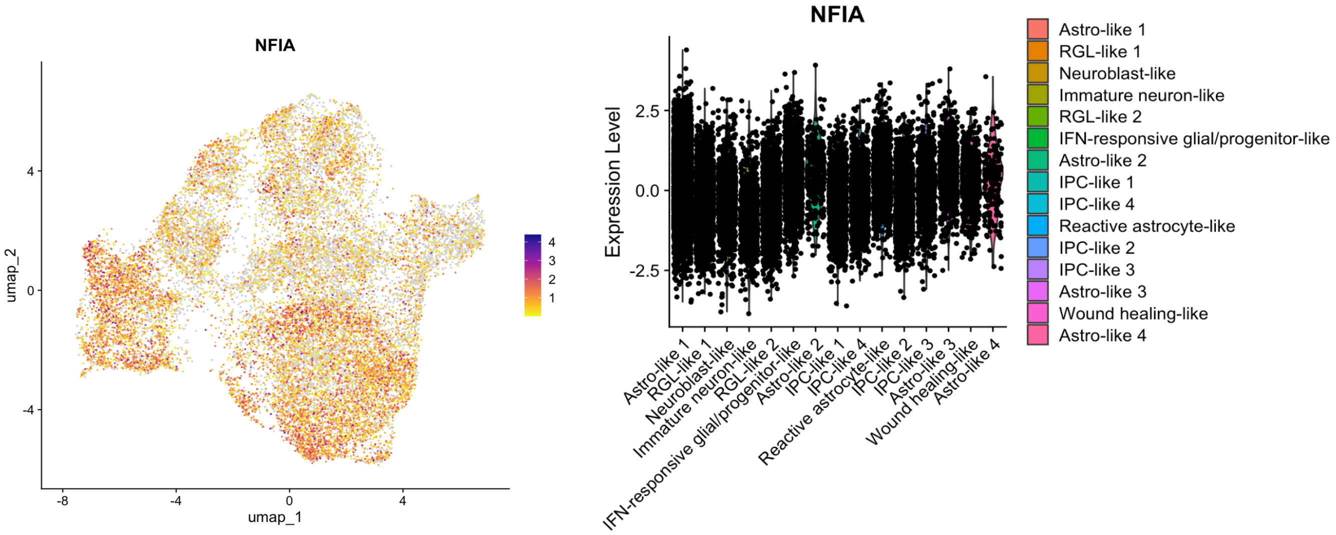

Figure 1:Predicted NF-1A activity demonstrated in UMAP (left) and violin plot (right).

2. In the revised manuscript, the authors examine caspase 3 (C3) to determine if TNF causes apoptosis. Although they find that TNF has not effect on the %C3+ cells, this does not mean that the TNF is not causing cell death in their model, as TNF can also trigger caspase-independent necrosis or necroptosis via RIPK1/RIPK3. Thus, it is still possible that the loss of clusters (or cells) without increases in progenitors or alternative lineages are due to cell death.

We appreciate the reviewer's point that CC3-independent cell death should be considered. To directly address this possibility, we interrogated our scRNA-seq dataset for expression of the core necroptosis machinery genes (*RIPK1*, *RIPK3*, and *MLKL*) (**new** panels e and f in Supplementary Fig. 10). *RIPK1* was broadly expressed across clusters, *MLKL* was detected only in a small minority of cells, and notably, *RIPK3* was not detected in any cluster/cells. Given that RIPK3 is indispensable for TNF-α–induced necroptosis (He et al., 2009), the absence of RIPK3 expression strongly suggests that RIPK1/RIPK3-dependent necroptosis is not a meaningful contributor to cluster loss in our model. These results have been incorporated into the revised manuscript (lines 160–164):

"As TNF-α-TNFR1 signalling can also induce regulated necroptosis via the RIPK1/RIPK3/MLKL pathway, we examined expression of these core necroptotic mediator genes (Supplementary Fig. 10e-f). RIPK1 was broadly expressed, whereas MLKL was detected in few cells and RIPK3 was absent across clusters, arguing against canonical RIPK1/RIPK3-dependent necroptosis."

Furthermore, while our data strongly excludes a CC3-dependent pathway, it remains possible that other pathways may be involved. We have therefore included the following text in the discussion at lines 455 - 457:

"*However, it remains possible that other CC3-independent mechanisms of cell death of differentiating neurogenic cells may be involved."*

In addition, if the neurogenic cells express the lowest amount of TNFR1, how is TNF signaling responsible for these effects?

The low levels of *TNFRSF1A*+ cells in the neurogenic clusters under control conditions suggests that TNF-α exerts its antineurogenic effect on the HPCs before differentiation into neuroblasts/immature neurons. Alternatively, the effect could be driven by paracrine type I IFN signalling from reactive astrocytes. We have clarified this by incorporating the following sentence (lines 447 – 450):

"*This pattern is consistent with TNF-α acting primarily on progenitor/early differentiation states, thereby biasing subsequent lineage decisions, and/or with paracrine type I IFN signalling from reactive glial cells driving the decreased neurogenesis*."

References

He, S., Wang, L., Miao, L., Wang, T., Du, F., Zhao, L., Wang, X., 2009. Receptor Interacting Protein Kinase-3 Determines Cellular Necrotic Response to TNF-α. Cell 137, 1100–1111. https://doi.org/10.1016/j.cell.2009.05.021
